# Towards Clinical Translation of Intravoxel Incoherent Motion MRI: Acquisition and Analysis Consensus Recommendations

**DOI:** 10.1002/jmri.70278

**Published:** 2026-03-19

**Authors:** Eric E. Sigmund, Susanne S. Rauh, Mami Iima, Christian Federau, Diego Hernando, Oscar Jalnefjord, Jacobus F. A. Jansen, Jonas Jasse, Neil Peter Jerome, Misha P. T. Kaandorp, Sila Kurugol, Frederik B. Laun, Mira M. Liu, Alexandra Ljimani, Thoralf Niendorf, David A. Reiter, Mohammed Salman Shazeeb, Amita Shukla‐Dave, Julia Stabinska, Andreas Wetscherek, Peter T. While, Dan Wu, Denis Le Bihan, Oliver J. Gurney‐Champion

**Affiliations:** ^1^ Department of Radiology NYU Langone Health New York New York USA; ^2^ Bernard and Irene Schwartz Center for Biomedical Imaging New York New York USA; ^3^ Center for Advanced Imaging Innovation and Research (CAI²R) New York New York USA; ^4^ Department of Radiology, C.J. Gorter MRI Center Leiden University Medical Center Leiden the Netherlands; ^5^ Department of Fundamental Development for Advanced Low Invasive Diagnostic Imaging Nagoya University Graduate School of Medicine Nagoya Japan; ^6^ Department of Neuroradiology University of Zürich Zürich Switzerland; ^7^ AI Medical AG Zollikon Switzerland; ^8^ Kyoto Prefectural University of Medicine Kyoto Japan; ^9^ Department of Radiology University of Wisconsin‐Madison Madison Wisconsin USA; ^10^ Department of Medical Physics University of Wisconsin‐Madison Madison Wisconsin USA; ^11^ Department of Medical Radiation Sciences, Institute of Clinical Sciences, Sahlgrenska Academy University of Gothenburg Gothenburg Sweden; ^12^ Department of Medical Physics and Biomedical Engineering Sahlgrenska University Hospital Gothenburg Sweden; ^13^ Department of Radiology and Nuclear Medicine Maastricht University Medical Center Maastricht the Netherlands; ^14^ Mental Health en Neuroscience Research Institute (MHeNs) Maastricht University Maastricht the Netherlands; ^15^ Department of Electrical Engineering Eindhoven University of Technology Eindhoven the Netherlands; ^16^ Department of Diagnostic and Interventional Radiology, Medical Faculty and University Hospital Düsseldorf Heinrich‐Heine‐University Düsseldorf Düsseldorf Germany; ^17^ Department of Circulation and Medical Imaging NTNU Norwegian University of Science and Technology Trondheim Norway; ^18^ Center for MR Research University Children's Hospital Zurich Zurich Switzerland; ^19^ Department of Radiology, Quantitative Intelligent Imaging (QUIN) Lab Boston Children's Hospital and Harvard Medical School Boston Massachusetts USA; ^20^ Institute of Radiology, Uniklinikum Erlangen Friedrich‐Alexander‐Universität Erlangen‐Nürnberg Erlangen Germany; ^21^ Department of Diagnostic, Molecular and Interventional Radiology, BioMedical Engineering and Imaging Institute Icahn School of Medicine at Mount Sinai New York New York USA; ^22^ Berlin Ultrahigh Field Facility (B.U.F.F.) Max Delbrueck Center for Molecular Medicine in the Helmholtz Association Berlin Germany; ^23^ Department of Radiology and Imaging Sciences Emory University Atlanta Georgia USA; ^24^ Department of Orthopedics Emory University Atlanta Georgia USA; ^25^ Department of Biomedical Engineering Georgia Technical Institute and Emory University Atlanta Georgia USA; ^26^ Department of Radiology University of Massachusetts Chan Medical School Worcester Massachusetts USA; ^27^ Department of Medical Physics Memorial Sloan Kettering Cancer Center New York New York USA; ^28^ Department of Radiology Memorial Sloan Kettering Cancer Center New York New York USA; ^29^ F.M. Kirby Research Center for Functional Brain Imaging Kennedy Krieger Institute Baltimore Maryland USA; ^30^ Division of MR Research, The Russell H. Morgan Department of Radiology and Radiological Science The Johns Hopkins University School of Medicine Baltimore Maryland USA; ^31^ Joint Department of Physics The Institute of Cancer Research and The Royal Marsden NHS Foundation Trust London UK; ^32^ Department of Radiology and Nuclear Medicine St. Olav's University Hospital Trondheim Norway; ^33^ Department of Biomedical Engineering, College of Biomedical Engineering and Instrument Science Zhejiang University Hangzhou China; ^34^ NeuroSpin, CEA‐Saclay Center Paris‐Saclay University Gif‐sur‐Yvette France; ^35^ Human Brain Research Center Kyoto University Graduate School of Medicine Kyoto Japan; ^36^ Department of System Neuroscience National Institutes for Philosophical Sciences Okazaki Japan; ^37^ Department of Radiology and Nuclear Medicine Amsterdam UMC Location University of Amsterdam Amsterdam the Netherlands; ^38^ Cancer Center Amsterdam Imaging and Biomarkers Amsterdam the Netherlands

**Keywords:** biomarkers, consensus, harmonization, intravoxel incoherent motion (IVIM), open science, recommendations

## Abstract

Intravoxel incoherent motion (IVIM) MRI allows for simultaneous assessment of tissue microcirculation (perfusion) and diffusion of water. In single‐center studies, IVIM has shown great potential for diagnosis, treatment outcome prediction, and treatment monitoring for many different diseases and organs. However, heterogeneity in data acquisition protocols, pre‐processing pipelines, and post‐processing routines yields differences in reported IVIM parameters, which has constrained large‐scale deployment of IVIM. Moreover, deploying IVIM protocols and analysis typically requires technical expertise, further challenging wider use, especially for clinicians. In this consensus paper, to accelerate the deployment of IVIM, we provide recommendations and harmonize protocols for brain, breast, kidney, liver, muscle, and pancreas IVIM studies. For this goal we organized multiple questionnaires and held a dedicated workshop. To ensure a level of standardized, reproducible results, without restricting innovation, we suggest a small subset of *b*‐values to always be measured and analyzed separately, and to which more extensive *b*‐value sampling can be added for advanced investigations. We further introduce detailed recommendations on acquisition protocols and analysis pipelines. To increase consistency, repeatability, and reproducibility, we highly recommend that these protocols and pipelines be deployed by scientists and clinicians for IVIM studies. For advanced users who desire different protocols or analysis approaches, we suggest adding results from our suggested protocols and analysis pipeline in the supplemental part of their paper to enable retrospective studies.

## 1 Motivation

Intravoxel incoherent motion (IVIM) is a diffusion‐weighted magnetic resonance imaging (MRI) concept that allows for simultaneous assessment of incoherent motion of water molecules, typically attributed to perfusion, and pure water diffusion due to Brownian motion, attributed to tissue microstructure, without exogenous contrast agents [1, 2, 3]. Perfusion and diffusion occur at different temporal and spatial scales. Diffusion‐weighted MRI (DWI) involving a range of different diffusion‐weightings (*b*‐values) permits these two processes to be distinguished within the IVIM model by fitting the two‐component signal decay. Particularly, the signal from medium to high *b*‐values (within the Gaussian diffusion regime 200 ≤ *b* < 1000 s/mm^2^) is sensitive mainly to tissue water diffusion, whereas signal from lower *b*‐values (*b* < 200 s/mm^2^) is sensitive to both diffusion and faster incoherent motion. By fitting the IVIM equation,

(1) $$S\left(b\right)/S\left(0\right)=f\cdot e^{-\mathrm{bD}*}+\left(1-f\right)\cdot e^{-\mathrm{bD}},$$

to the signal *S*, the IVIM parameters can be obtained: *f* is the pseudo‐diffusion signal fraction, D* the pseudo‐diffusion coefficient, and *D* the tissue diffusion coefficient. Note that D* includes the water diffusion coefficient in blood, *D*_*blood*_, and D* is typically (and throughout this paper) used instead of D* + *D*_*blood*_. Typically, the pseudo‐diffusion is attributed to microvascular perfusion, and *f* is most commonly referred to as perfusion signal fraction. More generally, various types of microcirculation can also be studied with IVIM, such as flow in larger vessels, tubular, and ductal compartments.

**FIGURE 1:**
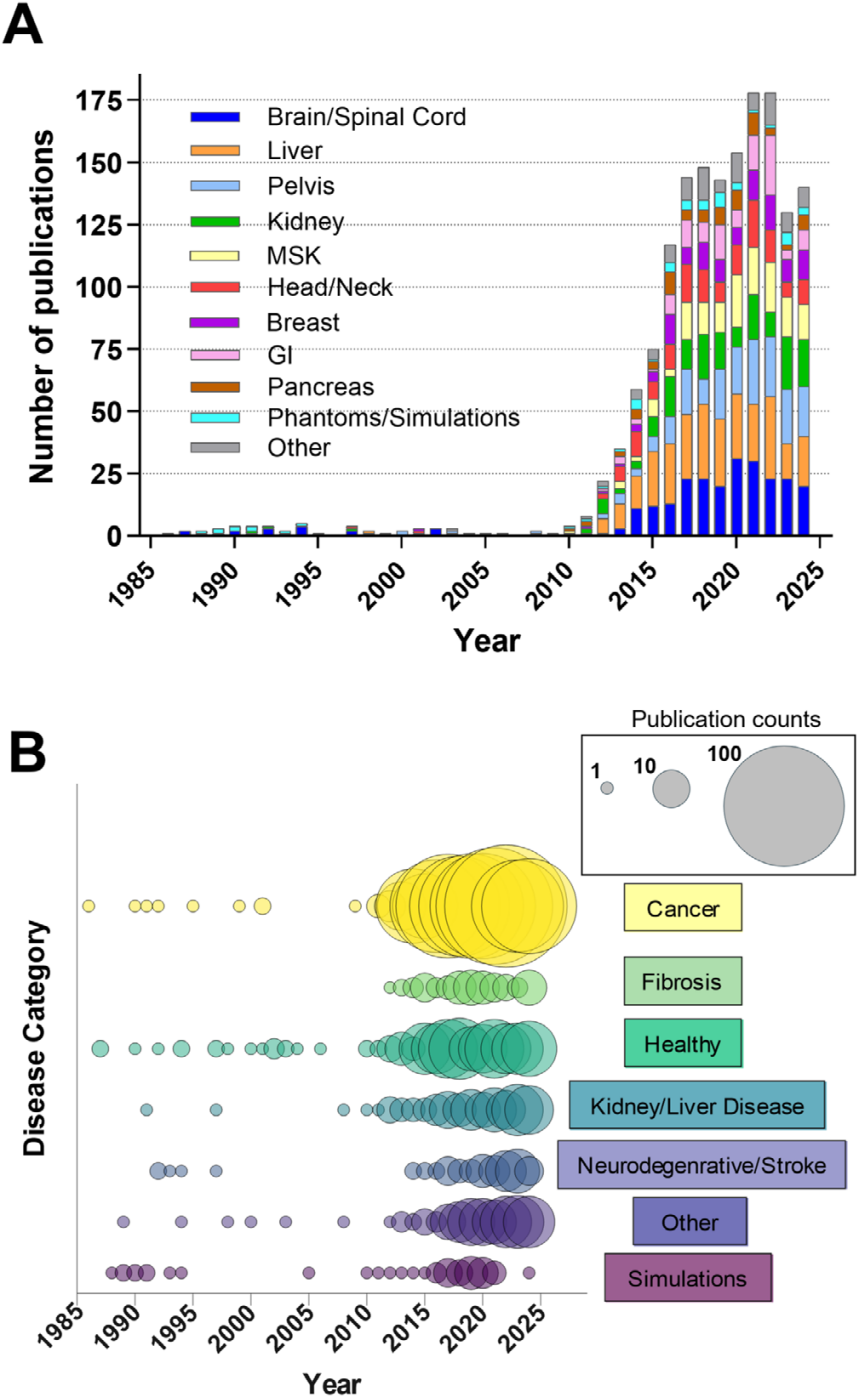
Timeline of publications related to the use of IVIM sorted by (A) organs and (B) disease categories. The organs brain/spinal cord, liver, and pelvic regions constitute the majority of the published studies. Cancer clearly dominates the majority of disease studies. A total of 1500+ research articles were included in this analysis obtained from PubMed as of August 13, 2024, using the keywords “intravoxel incoherent motion OR IVIM.” Review articles, commentaries, and editorials were excluded. Due to a vast coverage of organs in the IVIM literature, not all organs are depicted in this figure. The organs were consolidated as follows: Pelvis includes embryo/fetus/placenta, cervix/ovary/uterus, and prostate; gastrointestinal (GI) includes stomach, intestines, colon, and rectum; musculoskeletal (MSK) includes muscle, bone, and cartilage; Head/Neck includes eye, nose, and throat. For the disease categories: Cancer includes any type of tumor and metastasis; liver/kidney disease includes cirrhosis, fatty liver disease, and chronic kidney disease.

While DWI has become a pillar of clinical MRI since its inception (63,000+ publications on PubMed, August 13, 2024), IVIM has had a smaller representation in the literature primarily due to scanner hardware and MRI sequence limitations, but has been steadily growing in recent years (Figure 1). This can partly be explained by the extensive progress in MRI hardware and method developments; most vendors now offer a free choice of the number and values of the *b*‐values. Moreover, stronger magnetic field gradients and acceleration techniques facilitate the integration of IVIM exams into existing DWI protocols within reasonable acquisition times. Additionally, many vendors have started offering commercial inline image analysis pipelines for fitting the IVIM model. Finally, the ability to obtain an endogenous perfusion marker without the need for exogenous contrast agents makes IVIM an attractive technique when contrast agents cannot be used due to contraindications, in young patients, or in repeated MRI exams.

Compared to conventional DWI, IVIM‐derived *D* reflects the pure tissue diffusion coefficient, corrected for effects from blood, in contrast to the conventional apparent diffusion coefficient (ADC), which contains diffusion effects from tissue and blood. Moreover, IVIM provides additional parameters related to perfusion (*f*, D*).

**FIGURE 2:**
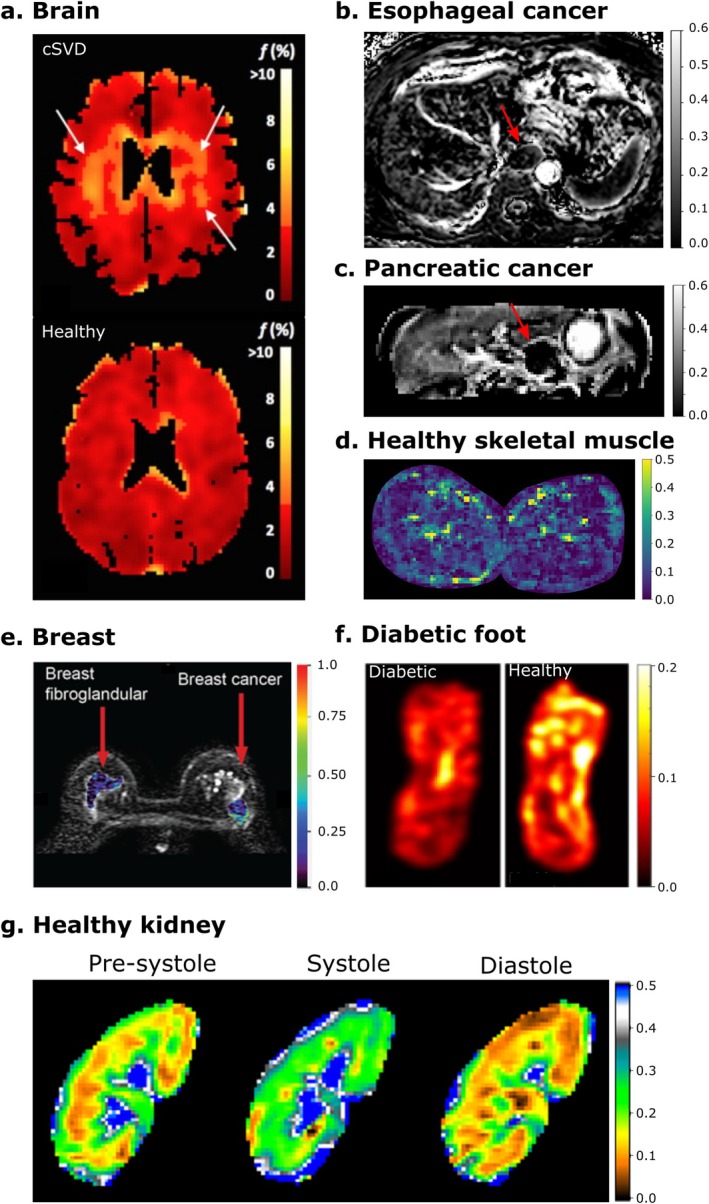
Examples of IVIM pseudo‐diffusion fraction (*f*) maps showing distinct patterns across different organs and cancers. (a) *f* maps of the brain of a patient with cerebral small vessel disease (cSVD) and a healthy control, showing regions with increased *f* in the cSVD patient. Figure modified from [30] with permission. (b, c) *f* maps of esophageal and pancreatic cancers demonstrating decreased *f* values in cancers compared to healthy tissue (arrows), showing hypo‐vascularity of those cancers. (d) *f* map of healthy skeletal muscles in the lower leg. The vessels are clearly visible by high *f* values, whereas the muscle tissue exhibits low *f* values. (e) Comparison of breast cancer and normal breast tissue, with breast cancer exhibiting higher *f* values in the periphery (rim‐enhancing) than normal breast fibroglandular tissue. (f) *f* map showing reduced pseudo‐diffusion fraction values in the plantar region of the diabetic foot compared with a healthy individual. (g) Sequential changes in renal *f* maps, showing variations in fraction values during pre‐systole, systole, and diastole phases of cardiac activity. Modified from Sigmund et al. [48] with permission. All examples came from ethics‐approved studies, and all patients gave written informed consent.

IVIM has demonstrated potential across various organs and pathologies, and systematic evidence can be found in a large body of literature. For example, in breast lesions, IVIM provided superior diagnostic performance compared to conventional ADC in differentiating malignant from benign tumors [4], and showed potential as a predictive marker for neoadjuvant chemotherapy response [5]. In kidneys, IVIM has demonstrated potential in detecting renal function decline, ureteral obstruction, fibrosis, chronic kidney disease, diabetic kidney disease, and renal cell carcinoma in patient studies [6, 7, 8, 9, 10, 11]. Specifically, cortical *f* showed distinction of chronic kidney disease in a meta‐analysis of 4 studies [10]. In the liver, IVIM has been used in a variety of diseases [12] and found useful in characterizing nonalcoholic fatty liver disease [13], liver fibrosis [14], and focal liver lesions [15]. In placental MRI, IVIM has shown potential in prenatal diagnostics by studying placental and fetal perfusion [16, 17]. In skeletal muscle, IVIM has distinguished differences in pseudo‐diffusion signal fraction at rest [18, 19] and during functional hyperemia [20, 21] in aging and disease, respectively. In the brain, IVIM can detect hyperperfused lesions such as high‐grade brain tumors [22, 23, 24] and hyperperfused state during hypercapnia [25, 26, 27] as well as hypoperfused lesions such as stroke and infarction [28, 29], and small vessel disease [30]. Regarding cancer, 18 meta‐analyses involving 9 different cancer types have been published [4, 31, 32, 33, 34, 35, 36, 37, 38, 39, 40, 41, 42, 43, 44, 45, 46, 47]. Among these, 13 reported area under receiver operating characteristic curve (AUC) values for clinical tasks (diagnosis, grading, and subtyping), averaging 0.89 ± 0.04 for *D*, 0.81 ± 0.07 for *f*, and 0.76 ± 0.08 for D*. Within this set of meta‐analyses in the oncologic sector, 12 studies also reported AUC values derived from ADC analysis, yielding 0.88 ± 0.04, 6 of which were lower than the highest IVIM‐derived AUC value in that study. Exemplary applications represented by the pseudo‐diffusion fraction maps are highlighted in Figure 2.

These strong indicators of clinical potential should also be considered in light of counterbalancing limitations, however. First, the IVIM sensitization of microcirculation and microstructure has a broad range of tissue context applications (an asset to be sure), all of which have their own clinical tasks and quantification of diagnostic accuracy, which limits opportunities for large scale data aggregation. Given the broad spectrum of MR acquisition techniques and settings (for instance, choice of *b*‐values, diffusion preparation, imaging read‐out) and data processing procedures (i.e., pre‐processing steps and fitting algorithms), heterogeneous and even contradicting findings have been reported for IVIM [22, 49, 50, 51, 52, 53]. These challenges have constrained large‐scale deployment of IVIM. Moreover, with the lack of clear and harmonized guidelines, deploying IVIM protocols and analysis typically requires on‐site (technical) experts, further hindering wider use, especially by clinicians. Therefore, we propose to accelerate IVIM deployment and translation by providing consensus recommendations and harmonized protocols.

To address these challenges, we organized multiple questionnaires and a dedicated workshop to develop community‐driven and harmonized recommendations for IVIM MRI. To promote the accumulation of compatible evidence without restricting innovation, our recommendations include tiered sets of *b*‐values (abbreviated, minimal, and advanced) with overlap of *b*‐values to maximize opportunities for future data pooling. Results from these harmonized protocols (abbreviated or minimal) can be reported in, for example, Supporting Information to improve repeatability and reproducibility and allow for meta‐analysis, without hindering authors' advanced methods and analysis. We further introduce more detailed recommendations on how to set up a protocol and analysis pipeline. We highly recommend that these protocols and pipelines are deployed by scientists and clinicians new to the field of IVIM MRI and for IVIM studies where there is no strong rationale for deviating from these settings to increase consistency, repeatability, and reproducibility.

## 2 Consensus Process

**FIGURE 3:**
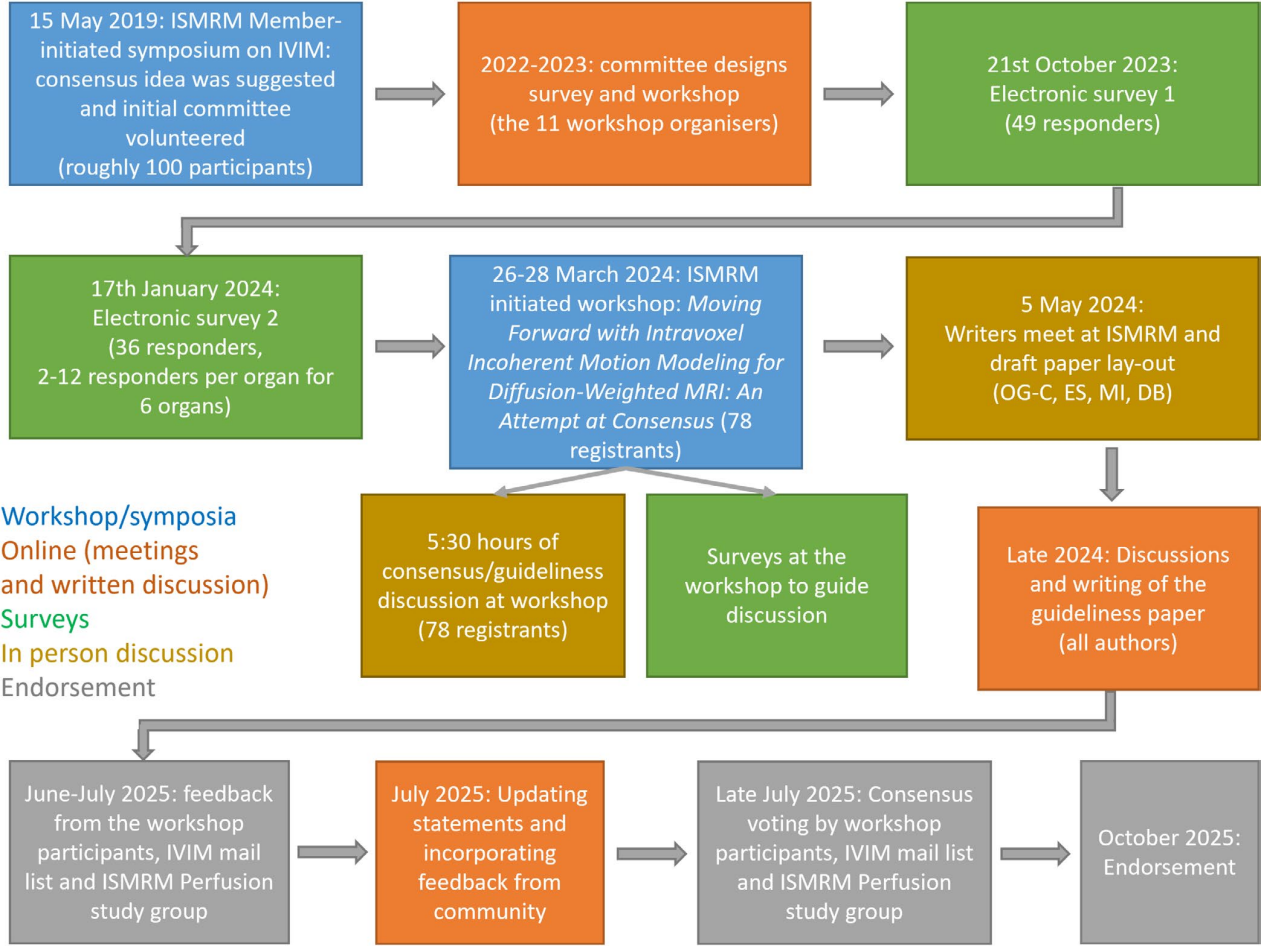
Schematic overview of the consensus process in the paper.

Several steps were taken to ensure that the recommendations outlined here reflect a consensus of the broader IVIM community (Figure 3). The consensus process was initiated at an IVIM symposium held at the annual 2019 ISMRM meeting (https://www.ismrm.org/19/program_files/MIS17.htm) and was consolidated starting in 2022, where the workshop committee conducted monthly online meetings to lay out the route to consensus, design surveys, and organize a workshop.

Using Google Forms, a survey was conducted among a wide range of scientists and clinicians experienced with (pre)clinical application of IVIM. Recipients included all members of a longstanding IVIM email list server and those added due to their literature contributions. The first part included questions on respondent training, target organ systems, and confidence assessment of IVIM use in various clinical indications. For each organ system receiving at least 10 responses, a second part included questions on the acquisition and modeling/processing of IVIM data for that organ. A full listing of questions is provided in Supporting Information S1: Survey Part 1.

Next, a dedicated workshop was held in March 2024 (https://www.ismrm.org/workshops/2024/IVIM/), featuring invited talks, proffered papers, and discussions. A total of 78 participants registered for the workshop. To help with forming recommendations, open‐floor discussions formed a large part of the scientific program. The results of the survey formed the basis for the workshop discussions. Input from the audience was further gathered using voting via Slido at the end of the workshop. Finally, a call for authors was held.

Following the workshop's outcome, the three lead authors drafted an outline of key sections of the manuscript for review by all co‐authors. After all co‐authors reached a consensus on the initial outline, all co‐authors contributed to designated sections of the full manuscript. On June 23rd, 2025, the draft of the manuscript was shared with all workshop attendees, the IVIM mail list participants, and the ISMRM Perfusion study group for careful revision and feedback. Finally, on July 29th, 2025 the paper was sent for an endorsement vote to the same groups. On October 25th, 2025 the paper was endorsed by workshop attendees, IVIM mail list participants, and the ISMRM Perfusion study group.

## 3 Survey Highlights

Full survey aggregate data are provided in the Supporting Information S1: Survey Part 1. A total of 47 respondents answered Part 1 of the survey. The majority (85.1%) were trained as MR researchers, and 21.3% were trained as radiologists. Tissue function and oncology were the dominant applications, with 68.1% of respondents reporting experience with both. The six organ systems with the highest respondent interest were: the brain, kidney, liver, muscle, pancreas, and breast.

**FIGURE 4:**
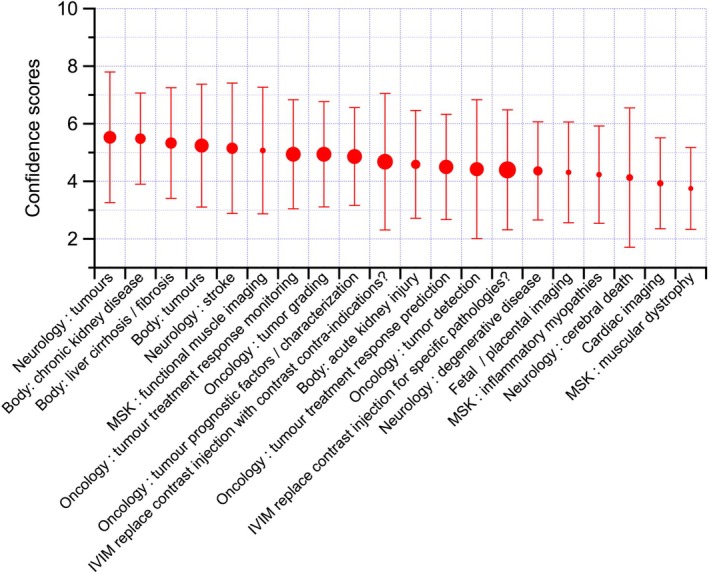
Ranked average and standard deviation of the confidence scores of IVIM application to clinical indications. Score specifications: 1: Should not be used; 3: Worth investigating; 5: Preliminary proof; 7: At a stage to set up a large clinical trial; 10: Should be used in a daily clinic with no further proof needed. The size of the dots indicates the number of responders, ranging from 12 to 40 responders. MSK, musculoskeletal.

Clinical indication confidence scores varied with application and organ system but showed averages close to the middle of the provided range (Figure 4). Indications with high confidence (> 5; defined as preliminary proof) included neurological tumors, chronic kidney disease, liver cirrhosis/fibrosis, body tumors, stroke, and MSK functional imaging. The confidence scores should be interpreted in light of the definitions provided to respondents. Scores of 7–8 imply readiness for large multicenter clinical trials, which typically require well‐established and standardized acquisition and analysis protocols. Consequently, the predominance of scores in the 4–6 range reflects the current developmental stage of IVIM, characterized by promising preliminary evidence but limited methodological harmonization. Rather than indicating a lack of confidence in IVIM as a technique, these results highlight the need for standardized guidance to enable robust multicenter studies and clinical translation, which is the primary aim of the present work.

The number of respondents to Part 2 of the survey varied from 12 (kidney) to 2 (pancreas, Supporting Information S2: Survey Part 2 Table S1). Supporting Information S2: Survey Part 2 shows acquisition preferences from Part 2 of the survey for each organ system. While MR acquisition preferences are organ‐specific and not universal, some aspects recur with strong levels of support, such as: magnetic field strength of 3 T for enhanced signal‐to‐noise ratio (SNR), single‐shot spin echo echo‐planar imaging (EPI) sequence, in‐plane spatial resolution ~2 mm, slice thickness 3–5 mm, repetition time (TR) 3–5 s, use of minimum echo time (TE), parallel imaging factor 2, spectral attenuated (or adiabatic) inversion recovery (SPAIR) based fat signal suppression, free breathing acquisition, and single refocused spin echo monopolar diffusion sensitizing gradient waveforms (Steijskal‐Tanner). Some respondents did not provide suggested *b*‐values, and some provided the same suggested set of *b*‐values.

**FIGURE 5:**
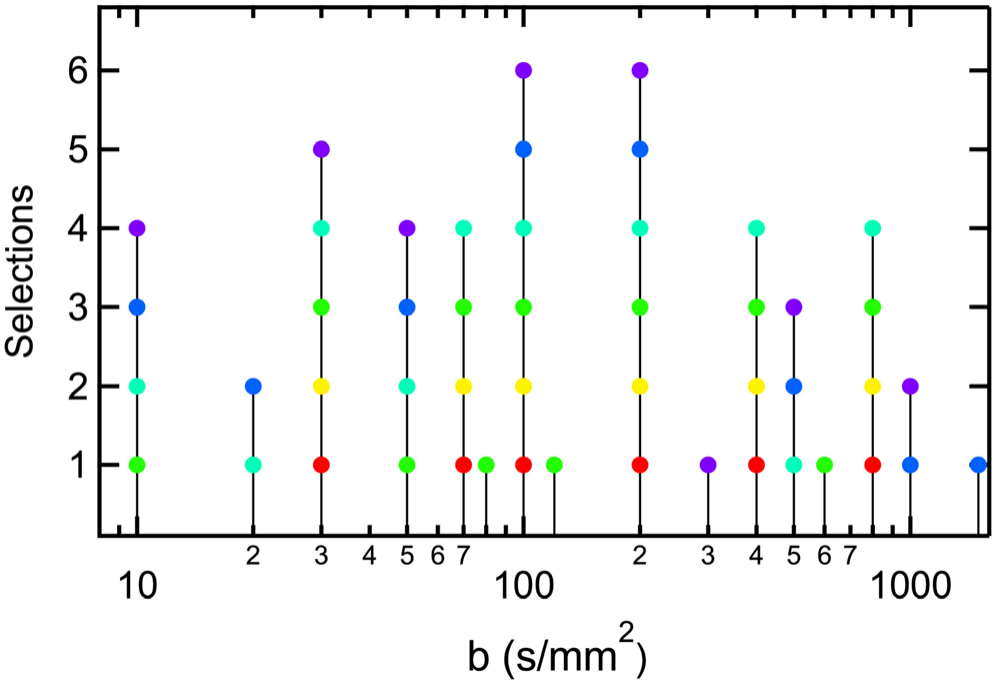
Cumulative distribution of suggested *b*‐values from 6 experts (color coded) reported for kidney applications in response to Survey Part 2.

The choice of *b*‐values was heterogeneous among respondents and organ systems. Figure 5 shows an example histogram of suggested *b*‐values of the correspondents in the kidney (organ with most respondents). Some *b*‐values (100, 200 s/mm^2^) were common to all submitted sets. For all organs, a slightly higher number of *b*‐values was suggested for IVIM in the lower *b*‐value range (10 ≤ *b* < 200 s/mm^2^) versus the upper range (200 ≤ *b* ≤ 1000 s/mm^2^) (Table 1), with an average value of 1.55.

**TABLE 1:** Numbers of suggested low and high *b*‐values for each organ system and the average number of unique *b*‐values.

Organ	Replies	Set #1: 10 ≤ *b* < 200	Set #2: 200 ≤ *b* ≤ 1000	Ratio #1/#2
Kidney	6	4.5	3.5	1.28
Breast	6	6.2	4.3	1.42
Liver	3	6.3	5.0	1.27
Muscle	3	6.3	3.0	2.11
Brain	3	6.7	4.3	1.53
Pancreas	2	5.0	3.0	1.66
**Average**	**3.8**	**5.8**	**3.9**	**1.55**

*Note*: The rightmost column shows the ratio of the two previous columns. Bold values indicate the average value.

**FIGURE 6:**
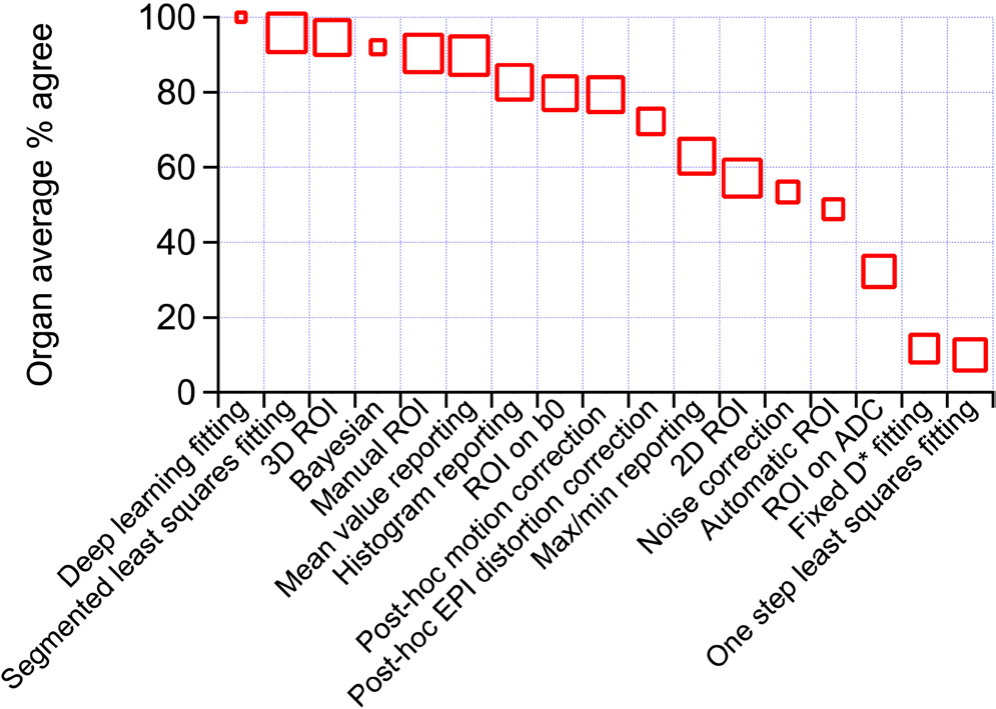
Ranked levels of agreement with the usage of particular analysis/reporting options of IVIM data, averaged over organ systems. Multiple selections were allowed. The size of the symbol is an indication of the number of respondents, ranging from 9 to 34. ADC, apparent diffusion coefficient; EPI, echo‐planar imaging; ROI, region of interest.

Figure 6 provides a summary of levels of agreement for a variety of pre‐processing and reporting alternatives, ranked in order of agreement. A full summary of these preferences stratified for each organ group is given in Supporting Information S2: Survey Part 2 Table S2.

Based on the questionnaire results, 23 additional polls were taken during the workshop to further specify the consensus. There was an average of 30 votes per poll (full questions and response data are given in Supporting Information S3: Workshop Polls). Recommendation‐specific results will be discussed in the respective sections. The top obstacles to clinical translation were deemed to be a lack of (1) inline processing, (2) demonstration of clinical sensitivity, (3) clinical understanding, (4) uniformity of pre‐processing or protocols, and (5) available test–retest data. Finally, a variety of these and other obstacles were suggested to be addressed next, particularly (1) standardization and validation (phantoms, *b*‐values, fitting, terminology/lexicon), (2) outreach (to clinicians and non‐research radiologists), (3) integration (Picture Archiving and Communication System (PACS) workflow, inline vendor processing), and (4) identification of high‐potential applications for inclusion in clinical trials and/or clinical routine.

These preferences, assessments, and future directions (some of which were also identified in the prior remote survey data) were taken into account in the recommendations that are given and discussed below, both for the utilization of IVIM in the present and its potential for future development.

## 4 Recommendations

The survey results demonstrate that there is not a one‐size‐fits‐all solution to IVIM. Therefore, here, we present recommendations for the six organs with the most survey respondents. Other organs have not been discussed at length during the workshop, and hence other considerations may lead to differing protocols. Absent other considerations, we would recommend following the liver recommendations for abdominal organs. For all other organs, we suggest the breast settings.

### 4.1 Data Acquisition

A summary of recommendations for IVIM data acquisition, stratified by organ system and based on the surveys, workshop, and post‐workshop discussions, is given in Table 2, and a summary of the *b*‐values in Table 3. As sufficient agreement existed among participants, our recommendations reflect parameter ranges that are compatible with at least 60% of pre‐workshop survey participants in each organ system. While agreement ranged between 60% and 100%, a recommended range is provided for all cases based on preferences and practical considerations to promote consistency between studies. Recommendations for the *b*‐values are summarized in Table 3 and are based on the survey and consensus discussion results (abbreviated tier) and on Cramer‐Rao lower bound simulations (minimal tier). Note that these recommended options (*b*‐values and other settings) are available by default or as a product on most modern MRI systems from most vendors. Here, we elaborate on the individual parameters in more detail.

**TABLE 2:** Recommendations for IVIM data acquisition and processing in human studies.

Protocol option	Brain	Kidney	Liver	Pancreas	Muscle	Breast
Field strength	1.5 or 3.0 T					
Sequence	Single shot EPI					
In‐plane resolution	1–3 mm	2–3 mm	2–4 mm	2–3 mm		1.8–3 mm
Readout FOV	200–270 mm	300–400 mm^a^				
Slice orientation	Axial	Coronal	Axial			
Slice thickness	1.2–3 mm	2.5–5 mm	4–6 mm	2–4 mm	4–6 mm	2–4 mm
Parallel imaging factor	2–3	2				
Fat suppression	SPIR (+SSGR if available)	SPAIR (+SSGR if available)				
TR (s)	≥ 4	≥ 3				≥ 4
TE (ms)	Minimum					
Averages	≥ 1				≥ 2	≥ 1
Diffusion gradients	Monopolar or bipolar					
# directions	≥ 3					

*Note*: The displayed ranges are compatible with a majority (> 60%) of pre‐workshop survey respondents and incorporate workshop and post‐workshop consensus discussions. Note that even given some range of parameters, these recommendations are provided for a non‐inferior approach to promote consistency between human studies. TR denotes the nominally selected repetition time or time between successive volume acquisitions. While in the case of respiratory gating, the effective TR can exceed this value, we include this suggested TR value as a lower bound.

Abbreviations: SPAIR, spectral attenuated inversion recovery; SPIR, spectral presaturation with inversion recovery; SSGR, slice selection gradient reversal.

^a^ Readout FOV for bilateral muscle imaging; for unilateral extremity imaging smaller FOVs are recommended. Note that *b*‐values are provided in Table 3.

**TABLE 3:** Suggested abbreviated (bold) and minimal (all) *b*‐values (s/mm^2^) for IVIM studies in each organ system.

Organ	*b*‐values (s/mm^2^)					
	#1	#2	#3	#4	#5	#6
Brain	**0**	0	70	**300**	400	**1000**
Breast	**0**	30	70	**200**	330	**800**
Kidney	**0**	30	70	100	**200**	**800**
Liver	**0**	10	20	100	**200**	**500**
Muscle	**0**	40	70	**200**	300	**600**
Pancreas	**0**	0	50	**200**	290	**600**

*Note*: The abbreviated tier was derived from survey results, consensus discussions, and literature values (for *b*_threshold_). The additional *b*‐values for the minimal tier were derived by Cramer‐Rao lower bound simulations. Note that for some organs, the Cramer‐Rao Lower Bound analysis suggested repeating the *b* = 0 s/mm^2^ instead of measuring a new unique *b*‐value.

#### 4.1.1 Field of View, Spatial Resolution, and Parallel Imaging

DWI commonly employs single‐shot echo planar imaging, which carries its own image quality challenges [54]. Field of view and in‐plane spatial resolution are two parameters that can differ per purpose. Diffuse diseases can typically be imaged with lower spatial resolution, whereas focal diseases require higher spatial resolution. Regarding parallel imaging with EPI, reasonable acceleration using parallel imaging techniques should only be performed when receiver radio‐frequency (RF) coils used for signal reception have sufficient receive elements along the phase‐encoding direction to support undersampling. The field‐of‐view and receiver bandwidth subsequently need to be adjusted to maximize SNR while managing EPI‐related image distortions. These limits will depend on the disease of interest and the purpose of the scan. In particular, for breast MRI, we advise using a cropped field of view combined with a posterior saturation band. For other organs, fold‐over suppression with saturation bands may be used to shorten the EPI echo‐train if the desired spatial resolution causes substantial distortions (bandwidth issue).

#### 4.1.2 Imaging Readout

While single‐shot EPI is the most commonly used and recommended imaging readout, we acknowledge the growing utilization of segmented (also known as multi‐shot) EPI approaches including segmentation along the phase‐encoding or the read‐out direction, for instance, in brain and breast studies [55, 56]. This approach significantly removes image distortion. If these protocols are utilized, we advise maintaining a similar SNR for IVIM analysis as with single‐shot EPI.

#### 4.1.3 Fat Suppression

The high sensitivity of EPI to chemical‐shift artifacts due to the fat‐water shift renders optimal fat signal suppression essential for IVIM MRI, especially in breast, abdominal, and muscle imaging. Spectral presaturation with inversion recovery (SPIR) and SPAIR are commonly used for fat suppression in clinical settings. Less common methods include short tau inversion recovery (STIR) [57] and slice select gradient reversal (SSGR) [58]. SPAIR is less sensitive to transmission field (B_1_) inhomogeneity [59] and, given sufficient magnetic field shimming (B_0_ homogeneity), provides more uniform fat suppression compared to SPIR. Therefore, SPAIR is recommended for breast, muscle, and abdominal imaging, with the addition of SSGR where possible. In the brain, SPIR is preferred to SPAIR as it offers acceptable fat suppression while allowing for shorter TRs.

#### 4.1.4 Respiratory Motion

Another critical aspect of the IVIM protocol, particularly for abdominal imaging, is respiratory motion compensation. Due to the fast 2D EPI readout, blurring and ghosting of individual diffusion‐weighted images is limited, but respiratory motion may still cause misalignment between slices, misregistration between diffusion‐weighted images and directions, and signal loss (due to intravoxel spin dephasing and/or poor overlap of the excitation and refocusing RF pulses) that may adversely impact the quality of IVIM MRI. This motion can partially be mitigated during acquisition using prospective motion correction (for instance, triggering), which synchronizes image acquisition with the respiratory cycle. Prospective motion correction is recommended in the abdomen if the examination permits a longer acquisition time. This is particularly important for focal diseases.

#### 4.1.5 Diffusion Gradient Waveform

Practitioners supported using twice‐refocused bipolar or single refocused monopolar gradient waveforms to varying degrees, neither achieving sufficient consensus to exclude the other at this stage, although monopolar waveforms are more common. Twice‐refocused diffusion sensitizing gradients allow for some eddy current compensation, background gradient effects mitigation and partial suppression of coherent blood flow effect, whereas monopolar gradients allow for shorter TEs, resulting in higher SNR.

#### 4.1.6 Repetition and Echo Time

The TR value should be chosen high enough to avoid a T_1_ bias in the perfusion fraction. The T_1_ values of the organs presented here range between 570/812 ms in the liver to 1080/1800 ms in gray matter at 1.5/3 T, respectively [60, 61, 62]. The TR values recommended in Table 2 ensure at least 2 * T_1_ recovery for the blood pool and each organ.

The use of higher *b*‐values results in longer diffusion gradients and, consequently, longer TE. Generally, we recommend using the lowest possible TE to maximize SNR. Nevertheless, it should be noted that the T_2_ values of tissues differ widely, from 40/30 ms for liver and muscle to 90/80 ms in the brain at 1.5/3 T, respectively [60]. Preferably, the TE should not exceed 2 * T_2_.

#### 4.1.7 Averaging

Using multiple averages in image space increases the SNR and can improve the data quality. This can be beneficial for high *b*‐values or regions with low SNR. For skeletal muscle, we recommend using at least 2 averages collected separately to enable correction of artifacts from involuntary contractions (muscle twitches) that may disturb the signal decay [63].

#### 4.1.8 Diffusion‐Weighting (*b*‐Values)

The range and number of *b*‐values directly impact the estimation of IVIM parameters and thus are important acquisition parameters and key for harmonization. Our recommendations are based upon several factors: (1) literature meta‐analyses/review articles on reported IVIM metrics in different organs; (2) quantitative guidance for minimization of bias and uncertainty; (3) current practices of active IVIM practitioners; (4) principles of harmonization that may permit maximal data pooling in future cross‐site data acquisition and analysis.

**FIGURE 7:**
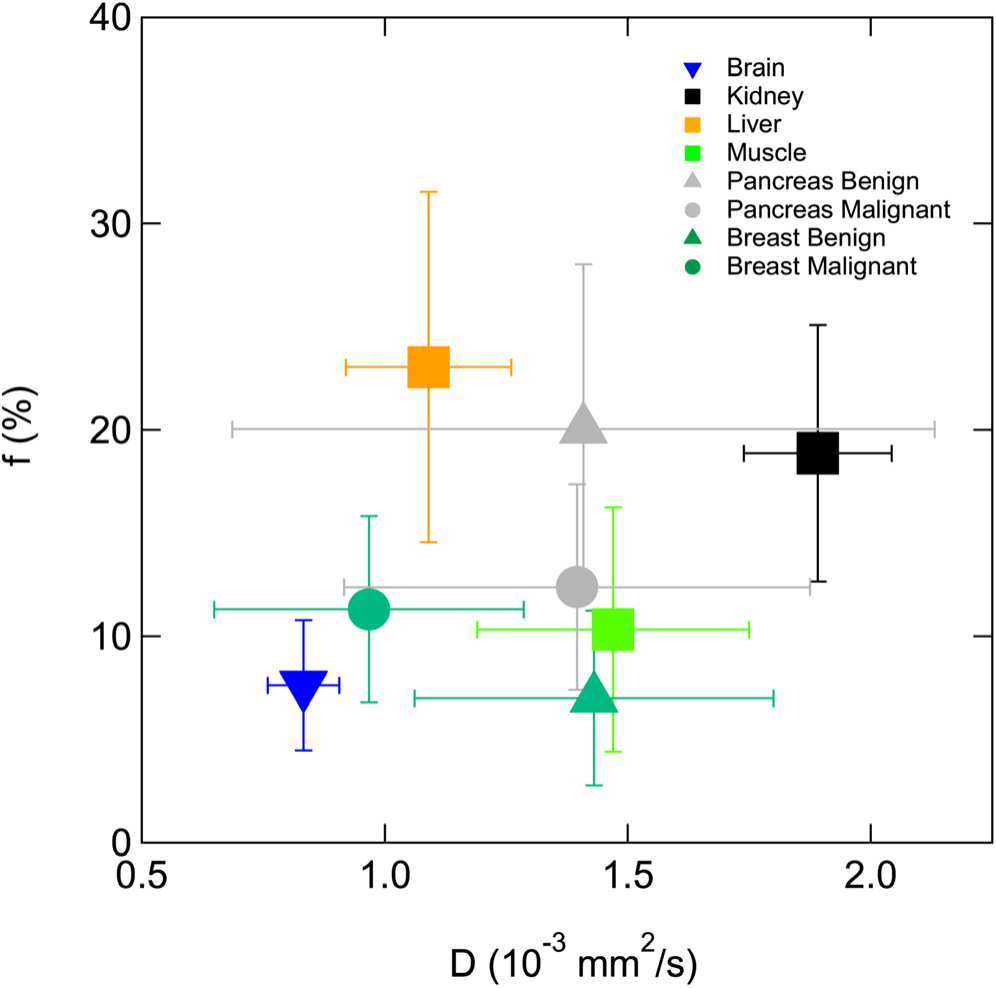
Mean IVIM values and standard deviations over studies (tissue diffusivity *D* and pseudo‐diffusion fraction *f*) derived from meta‐analyses and review articles for 6 organ systems.

Average IVIM values collected from meta‐analyses and review articles [4, 41, 64, 65, 66, 67] in different organ systems show notable variations and support optimized *b*‐value sets per organ (see Figure 7 and Supporting Information S4: Recommendations, Table S1).

**FIGURE 8:**
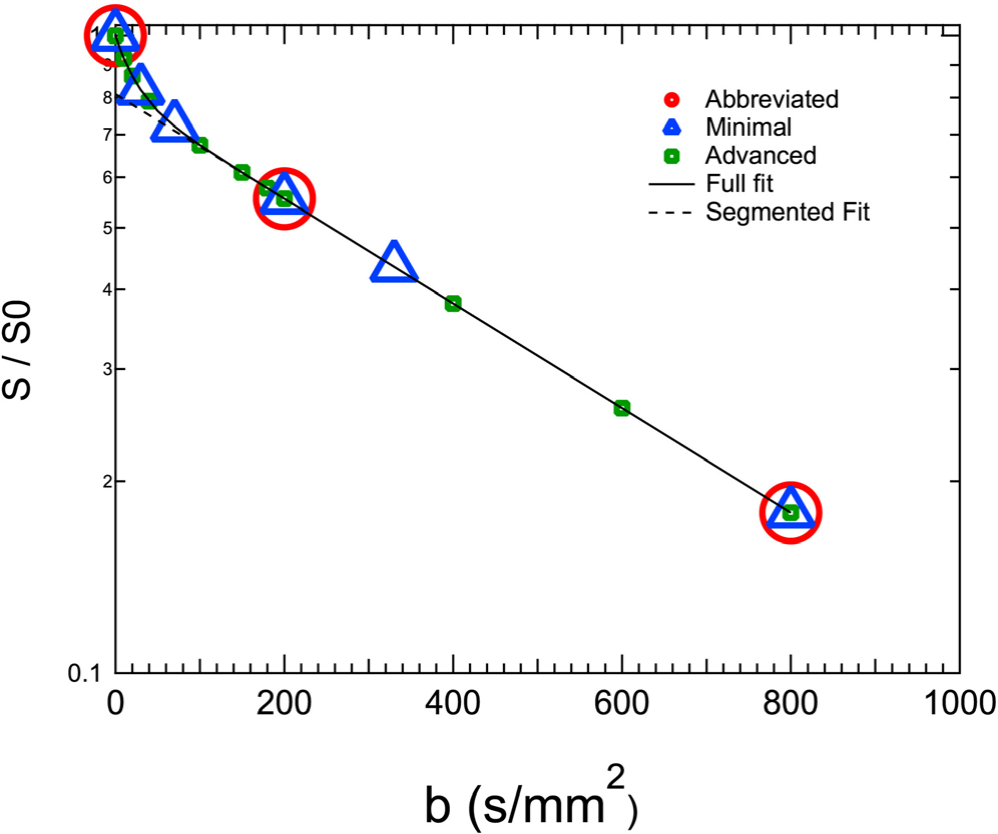
Tiers of suggested *b*‐values for IVIM studies, illustrated for breast: abbreviated (red circles), minimal (blue triangles), advanced (green squares).

Taking into account varying experience levels and goals of prospective IVIM practitioners, our suggested sets are organized in three “tiers,” which have overlapping *b*‐values: “abbreviated,” “minimal,” and “advanced.” This approach supports future pooled analysis of subsets of data from different studies or sites. An example of the tiered *b*‐value sets is depicted schematically in Figure 8; note that particular values (listed in Table 3) are different for each of the 6 focus organ contexts. The simplest, “abbreviated” protocol consists of the sampling of 3 *b*‐values (including *b* = 0 mm^2^/s), all with 3 orthogonal diffusion directions for trace‐weighting, to derive *D* and *f* from a segmented fit approach. This small set is the minimum number of *b*‐values needed to obtain these two IVIM parameters (sometimes referred to as abbreviated IVIM; hence the term) and is meant for studies in which no additional scan time is available. The “minimal” tier includes the 3 abbreviated values plus 3 additional *b*‐values, enabling all three IVIM parameters (*D*, *f*, D*) to be estimated within a limited scan time. The “advanced” tier permits expanding the “minimal” tier at the discretion of the investigator and can include many additional *b*‐values and diffusion directions to allow improvement in parameter estimates and advanced quantification models (such as compartmentation, diffusion spectrum analysis, anisotropy, diffusion time effects), but should again at least contain the *b*‐values from the minimal tier.

To generate these *b*‐value sets, we first selected a threshold *b*‐value for segmented (two‐step) fitting. The cut‐off value, *b*_threshold_, was determined to have a negligible contribution of perfusion‐driven IVIM effect above this value, making the diffusion part of the signal immune to this component. Our recommendation is to include the *b*‐value *b* = 200 s/mm^2^ for all organs (except the brain, which includes *b* = 300 s/mm^2^) to be used as *b*_threshold_. This recommendation is based on (1) expert opinions, (2) a balance between bias and variance, (3) commonly used values in literature [12, 67], (4) simulations, and (5) consistency where possible across applications. *b*_threshold_ must be sufficiently low to ensure a broad range of signal intensities to determine *D* and extrapolate to signal intercept at *b* = 0 s/mm^2^ to determine *f*, reducing variance in both. To estimate contribution of bias, the *b*‐value corresponding to suppression of the pseudo‐diffusion term by a factor of 20 (*e* ^−bD^* ~ 0.05; *b* ~ 3/D*) was computed using literature D* values from Supporting Information S4: Recommendations, Table S1, and for all cases except the brain, these thresholds were < 200 s/mm^2^ (for brain *b*_threshold_ = 275 s/mm^2^).

The *abbreviated tier b*‐value set (Table 3) consisted of *b* = 0 s/mm^2^, the threshold *b*‐value, and a selected upper‐limit. This *b*‐value upper limit was selected (a) to avoid sampling non‐Gaussian diffusion/kurtosis effects, (b) considering typical T2‐relaxation‐weighted SNR for each organ, and (c) to align with survey input, consensus discussions, and expert opinion. These upper limits were *b* = 500 s/mm^2^ for liver, *b* = 600 s/mm^2^ for muscle and pancreas, *b* = 800 s/mm^2^ for kidney and breast, and *b* = 1000 s/mm^2^ for brain.

Finally, the *minimal tier b*‐value sets were based on a Cramer‐Rao Lower Bound analysis [68, 69] and assumed Gaussian noise. We note that this noise model is only an approximation to the Rician noise distribution encountered in commonly magnitude‐averaged DWI signals and is thus a limitation. However, we assert that the main features of the suggested *b*‐value distributions are retained in this case and confer the intended organ‐specific optimality. The cost function penalized an error metric consisting of the sum of relative uncertainties in *D* and D* and absolute uncertainty in *f* (to avoid divergence for *f* ~ 0). For each organ context, optimization was performed over a range of parameters centered on the literature mean value (Supporting Information S4: Recommendations, Table S1) and spanning one standard deviation above and below it (in the case of breast and pancreas, averages of the benign and malignant literature values and a sum of their standard deviations was used). The code of the Cramer‐Rao Lower Bound analysis is available on Google Colab (https://colab.research.google.com/drive/1ffm3vqbeMMMvm9LvTV06ZuQKqbnc3hlf#scrollTo=23K0kjHtO1RT). A common SNR of 25 was assumed for all cases to set a reasonable worst‐case scenario.

A recent consensus effort for kidney IVIM [65] heuristically suggested *b*‐values (0, 30, 70, 100, 200, 400, 800 s/mm^2^) with considerable overlap to those generated by the process herein (0, 0, 30, 150, 200, 800 s/mm^2^). The difference in estimated relative parameter uncertainty between the subset (0, 30, 70, 100, 200, 800 s/mm^2^) and (0, 0, 30, 150, 200, 800 s/mm^2^) was 2%, 8%, and 3% for *D*, *f*, and D*, respectively; given this minimal difference, the former subset was adopted to support continuity of evidence generation. The final optimal sets (rounded to the nearest 10 s/mm^2^) are outlined in Table 3. Note that these sets have at least as many *b*‐values < 200 s/mm^2^ as greater than or equal to that threshold, consistent with the general survey trend in Table 1.

The aforementioned algorithm utilizes a generic process for all organs while incorporating organ‐specific parameter values, but nevertheless, it still has limitations. The ranges adopted from the meta‐analyses in Supporting Information S4: Recommendations, Table S1 incorporate major organ differences and reported variability but not healthy/pathologic ranges in all cases. We emphasize that these values compose only the abbreviated and minimal sets. Studies may employ more pathology‐specific *b*‐values at their discretion and as studies warrant, including dynamic and longitudinal studies where *D*, *f*, and D* may change over time.

We recommend using, at minimum, the abbreviated tier of 3 *b*‐values, 3 directions, and at least 1 average (for muscle at least 2 averages). In practice, given the typically poor SNR, we suggest substantially more averages with a minimal tier having at least two *b*‐values between 0 and 200 s/mm^2^. However, given the broad applications of IVIM in different organs, it is within the practitioner's discretion to increase the number of directions (for tensor imaging), increase averages, or increase the number of *b*‐values.

It is important to note that imaging gradients and spoilers also add diffusion‐weighting, effectively increasing the *b*‐value. As a result, the nominal *b*‐value might differ from the true *b*‐value if these gradients are not taken into account [70]. This is especially pronounced at low *b*‐values and can cause a substantial bias in the IVIM estimates. However, correcting for *b*‐values can still be challenging. In particular, it is not always possible to obtain the true *b*‐value, and there is a need for vendors to add them to DICOM headers. Due to those challenges, using corrected *b*‐values is not included as a recommendation. If available, we encourage the use of corrected *b*‐values. We encourage further research on the effect of corrected *b*‐values for IVIM.

We also provide Supporting Information to guide study design in the form of minimal suggested SNR to achieve total relative error (combining elements of bias and dispersion) of less than 20%, generated from Monte Carlo simulation studies centered around the mean values in Supporting Information S4: Recommendations, Table S1 and *b*‐value samples in each organ system from Table 3. Full details on these estimations are provided in Supporting Information S4: Recommendations Text S1, Figures S1 and S2, Table S2.

### 4.2 Data Pre‐Processing

Data pre‐processing is a key step in generating high‐quality IVIM maps from diffusion‐weighted images. In a review of pre‐processing for DWI, 15 steps were identified as essential [71]. While this review primarily targeted conventional diffusion and diffusion tensor MRI of the brain, many steps can be applied to IVIM and across other anatomical regions. In our survey of IVIM in particular, four key pre‐processing steps were identified: motion correction, EPI distortion correction, denoising, and removal of signal voids. These pre‐processing steps are summarized in Table 4. These steps are most effectively applied to individual *b*‐value images (separate averages and directions), and we therefore advise against inline averaging/trace‐images.

**TABLE 4:** Suggested pre‐processing for IVIM data, derived from the survey results.

	Brain	Non‐brain (abdomen, breast, muscle)
Motion correction via registration	Yes	Yes
Distortion correction	Yes	If motion is limited in the source‐images
Denoising	If extensive protocol	If extensive protocol and motion is limited in the source‐images
Signal void exclusion	N/A	Yes

*Note*: Extensive protocol: Denoising algorithms rely on the redundancy of data and sufficient *b*‐values or diffusion directions should be sampled if denoising is used.

Abbreviation: N/A, not applicable.

For clinical workflows, inline pre‐processing may be easier to achieve. In this case, please use the subset of tools available on your MR operator console or remote analysis computer. Registration and denoising are typically available on scanners, but inline processing may mean skipping denoising and signal void exclusion. Therefore, the offline setting is preferred, whilst the inline alternative is intended to provide a workflow feasible in a clinical setting.

#### 4.2.1 Motion Correction

Retrospective bulk motion correction based on an affine registration to a *b* = 0 s/mm^2^ image is recommended as a minimum baseline method to deal with rigid and affine motion and image deformations due to eddy currents. Using EPI, slices are acquired rapidly, minimizing within‐slice motion artifacts, but slice‐to‐slice motion can still occur and needs to be corrected [72, 73]. In regions with more complex motion, non‐rigid registration methods are suggested if available [74, 75, 76]. In the abdomen, image registration can be combined with prospective motion correction, which produces images of superior quality compared to the individual methods [77].

#### 4.2.2 EPI Distortion Correction

We recommend using EPI image distortion correction. EPI distortions cause deformations of the images and can cause signal pile‐up and signal voids, resulting in misaligned anatomy and distorted parameter estimates. EPI distortion correction should be applied using a method based on reversed phase encoding [78], if this can be achieved in a robust fashion. The distortion effect is pronounced in the vicinity of (air‐)tissue interfaces, such as those found around the kidneys, pancreas, liver, bowel, and in regions close to the sinuses or cavities. Additionally, the distortion field in the abdomen changes with respiratory and bowel motion. Therefore, the best results are found when estimating the distortion field for each read‐out individually. Previous studies performed this by using multi‐echo imaging [74] or scout acquisitions throughout the respiratory cycle [79] with reverse polarity acquisition.

#### 4.2.3 Denoising

We recommend denoising the diffusion‐weighted images; preferably, these include separate averages and directions, but for some studies, only trace‐weighted images are available. Denoising of diffusion‐weighted images typically exploits the redundancy available following the acquisition of several *b*‐values and diffusion encoding directions. This is often done using principal component analysis and, more recently, deep learning [80, 81], which have both been applied to IVIM [48, 82, 83]. Applicability of such algorithms may depend on the level of redundancy in the data; for the abbreviated or minimal acquisition protocols with only three to six *b*‐values, such redundancy may be unavailable, and in those cases, denoising may not be as effective. Potentially, deep‐learning‐based denoising may offer a solution there.

It is important to note that denoising methods can alter the original signal at different *b*‐values. Therefore, these methods should be used with caution and data consistency checks should be performed.

#### 4.2.4 Removal of Signal Voids

When present, we recommend the removal of signal voids due to bulk motion or incoherent motion other than perfusion. Signal voids can occur in scenarios involving pulsatile or respiratory motion (particularly in the left liver lobe and pancreas) or involuntary contractions of skeletal muscle [63, 84, 85, 86]. Their most direct solution is manual inspection and removal of the corresponding data from the image set, assuming that sufficient redundancy exists in the sampling and inline averaging has not already been performed.

### 4.3 Fitting

Most MRI vendors support in‐line IVIM fitting (potentially as an additional product), and there are commercial third‐party solutions available, as well. These solutions are CE‐labeled and hence can be used in the clinic. For this reason, there is certainly merit in reporting results from such software. However, vendor implementations may differ from each other, may change with updates, and vendors are not always transparent in their exact implementation. Therefore, for reproducibility purposes, we recommend also reporting results from a standardized fitting routine.

Fitting the IVIM model to noisy DWI is an ill‐conditioned problem, where the precision and accuracy of conventional (non‐linear) least‐squares (NLLS) estimators are limited for the pseudo‐diffusion parameters, especially in regions of low SNR and low pseudo‐diffusion signal fraction. Many alternative approaches have been proposed [87], ranging from simple asymptotic “segmented” approaches [88, 89] to more advanced Bayesian inference [90, 91, 92] or deep learning [93, 94]. The majority of studies use segmented fitting, which is straightforward to implement and appears more robust than conventional simultaneous least‐squares fitting. This trend in the literature was reflected in the results of the survey and workshop discussions, where further development of advanced estimators was supported (see Figure 6), while segmented fitting was the approach recommended to be included in the baseline protocol for IVIM pre‐processing.

Segmented fitting relies on the assumption that D* ≫ *D*, such that the contribution of the pseudo‐diffusion component to the signal attenuation becomes insignificant at sufficiently high *b*‐values. This permits a two‐step fitting procedure, whereby first, only the diffusion component is fitted to the data using only *b*‐values larger than a predefined threshold. This can be achieved by fitting a mono‐exponential function to the signal data using NLLS, or by fitting a linear function to the logarithm of the signal data using weighted linear least squares (WLLS), ideally iteratively [95]. We recommend the use of iterative WLLS as it does not require initial guesses, and it is faster than NLLS. However, we acknowledge that applying NLLS for this first step may simplify implementation depending on the software used and typically should give similar results given appropriate initial guesses. Note that taking the logarithm of the signal can enhance noise at high *b*‐values in conditions where SNR is low, so caution should be taken in such cases. As *b*‐value threshold, we recommend using *b* = 200 s/mm^2^, except for the brain (*b* = 300 s/mm^2^), as defined in Section 4.1.

The first step also yields an estimate of *f* from the difference between the signal at *b* = 0 s/mm^2^ and the corresponding vertical intercept of the initial fit. In the second step, D* is estimated by fitting the full IVIM equation to the data at all *b*‐values using NLLS (recommended initial guess D* = 10 × *D*), after either fixing both *D* and *f* from the first step or fixing only *D* and re‐solving for *f* [96]. As the workshop discussions indicated a slight preference for fixing both *D* and *f*, we recommend this approach to increase consistency.

Note that segmented fitting can introduce a positive bias in the estimation of *D* and a negative bias in the estimation of *f*, owing to the finite pseudo‐diffusion component that may remain at the chosen threshold *b*‐value [88, 97, 98]. Note further that this implementation of the segmented fit relies on measuring the signal at *b* = 0 s/mm^2^, which cannot be exactly measured due to the contribution from imaging gradients and spoilers to the *b*‐value. Those contributions are neglected in this implementation; we offer some discussion on how to deal with this in Supporting Information S5.

### 4.4 Reporting Preferences and Good Practice

We expect the recommended protocol to serve well under many circumstances, and for a broad range of applications. However, we understand that it may not meet the demands of every conceivable IVIM study. Nonetheless, it would be desirable to put more advanced studies on a similar footing to allow for data pooling, provide some level of data comparability, and quantify the added value of the optimized research protocols as compared to standardized analysis. The recommendations in this section are based on the survey results and consensus discussions during the workshop.

Regarding region of interest (ROI) prescription, the unweighted (*b* = 0 s/mm^2^) image was preferred in the majority (4/6) of organ systems in the surveys as it contains the highest SNR, making the contouring of organs easiest. Some pathologies, such as cancer, are most visible on high *b*‐value images and/or *ADC* maps. Hence, we encourage adherence to *b* = 0 s/mm^2^ images with secondary consultation of *ADC* map and/or high *b*‐value images.

While the acquisition protocols may heavily depend on the intended study purpose, we recommend reporting the results of a separate sub‐analysis that is streamlined with the suggested protocol and pre‐processing steps. We further recommend reporting the within‐ROI values that were rated highest in the standardized pipeline poll (see Figure 6): mean, standard deviation, median, and interquartile range of ROI statistics for completeness and meta‐analysis. Then, further paper‐specific analysis should reflect the data normality.

To support and ensure reproducible research and maintain consistent relaxation weighting, we advise comprehensive reporting of acquisition timing: TR, TE, and diffusion gradient waveform, including the diffusion time (delay between the pulsed magnetic field gradients [Δ], the duration of the pulsed magnetic gradients [*δ*], and the maximum gradient strength and slew rate of the MR system). Ideally, a comprehensive description of all used MRI protocols should be provided as Supporting Information, including type of fat suppression, acquired voxel size, echo train length, echo spacing, receiver bandwidth used for signal read‐out, acceleration options, oversampling factors and directions, and slice gap and slice acquisition order. Also, reconstruction settings should be reported if available, including interpolation, zero filling, and filtering. Moreover, details from pre‐processing and fitting should be stated, including details of individual pre‐processing steps, fit algorithm, initial guess, and fit constraints. A checklist of parameters to report can be found in Supporting Information S6: Reporting Checklist.

There is a strong TE dependency of the pseudo‐diffusion signal fraction, as the diffusion and pseudo‐diffusion compartments may have different T_2_ relaxation times [99, 100]. For example, de Bazelaire et al. [61] reported liver T_2_ times of 46 ms (1.5 T) and 34 ms (3 T), whereas Riexinger et al. [101] estimate the T_2_ times of arterial and venous blood as 148 ms (venous, 1.5 T), 44 ms (venous, 3 T), 206 ms (arterial, 1.5 T), and 107 ms (arterial, 3 T). When interpreting these population‐averaged relaxation time values, it is important to note that intersubject variability can be substantial; for example, blood relaxation times depend on hematocrit [102]. If T_2_‐values are known, a T_2_‐corrected *f* can be reported [103], changing *f* from being a signal fraction to a blood fraction. It has been proposed as good practice to report such metrics for muscle and liver IVIM [67, 99, 100]. In the workshop, we did not reach an agreement on whether reporting T_2_‐corrected IVIM metrics should be regarded as best practice. One challenge we discussed was lack of consensus on how to perform the correction appropriately. For example, arterial and venous blood have vastly different T_2_ relaxation times, so one would have to take into account their volume fractions [104]; moreover, in the kidney, primary urine, and the tubular fluid would also have to be considered [105]. Moreover, a T_1_ correction might also be important [106]. Therefore, we advise always to report the used TE and TR times, such that corrected/uncorrected *f* values can be calculated post hoc for meta‐analysis. Second, IVIM contrast can vary with sequence timing for other biophysical reasons such as ballistic/diffusive regimes and degree of motion compensation [107, 108]. These are discussed in the section of future directions but their existence is another motivation for comprehensive reporting.

We recommend using validated and verifiable software. That is, the evaluation code should have been tested and validated on synthetic data and ideally, the code is provided open access, or vendor‐provided software packages should be used. We highlight that OSIPI [109] offers open‐source unit‐tested IVIM code at https://github.com/OSIPI/TF2.4_IVIM‐MRI_CodeCollection.

With the objective of translating IVIM into quantitative imaging biomarkers for clinical trials, and ultimately into clinical practice, it is of paramount importance to test and validate the robustness of the IVIM‐derived metrics [110]. The quantitative imaging network communities recommend performing a test–retest study prior to designing and executing the clinical study. Alternatively, literature may be consulted to provide guidance; several studies have performed IVIM repeatability (test–retest) and reproducibility (cross‐vendor/cross‐site) studies. Generally, the highest reproducibility values have been reported for *D* and *ADC*, ranging from good to excellent (coefficient of variation [CV] ~ 1%–10%), with good to poor reproducibility (CV ~ 10%–40%) for *f* and D*; this has been shown for hepatocellular carcinoma and liver parenchyma [111], renal tumors, normal renal cortex and medulla [112], and brain [113, 114]. Consequently, continued measurement and improvement of repeatability and reproducibility of *f* and D* is crucial. An extensive review of previous test–retest studies and reproducibility results from literature is given in Supporting Information S7: Test–retest studies.

## 5 Future Directions

We conclude this work with a discussion of the recommended focus areas and frontiers for translation and advancement of IVIM in the coming years.

To underline its importance, we start by stating we strongly encourage the IVIM community to reach out to the clinical community about the added value of IVIM. This includes continued efforts at large scale, such as data summaries (review articles, meta‐analyses) and multi‐center trials to elevate and clarify the evidence level of the clinical applications of IVIM. Such efforts pave the way for consideration and inclusion of IVIM in clinical trials, recommendations to the scientific and clinical societies, and ultimately governmental approvals. The process employed here by the consensus participants may also be revisited for other organ contexts (e.g., prostate, placenta) when researchers' demand, evidence accumulation, and/or compelling clinical applications coalesce in those cases.

IVIM is already used as a tool to study physiological and pathophysiological conditions in cohorts, for example, to understand disease onset and progression better, or to develop and validate treatment pathways. If accurate and precise enough, quantitative perfusion imaging with IVIM has great potential for diagnosis, treatment stratification, treatment monitoring and follow‐up imaging in individual patients. One first application of IVIM may be as an alternative to contrast‐enhanced MRI for imaging tissue perfusion, which is used in many clinical routines. IVIM parameter maps of *D*, D*, and particularly *f*, might be used to replace the contrast‐enhanced images, particularly for existing contexts in which quantitative visualization is key (e.g., pre/post contrast difference). Obstacles to the use of exogenous MRI contrast agents are the risks of nephrogenic systemic fibrosis (NSF), gadolinium deposition in tissues, and environmental impact. In this context, IVIM MRI, as an endogenous contrast alternative for assessing perfusion and cellular density, could become a highly valuable tool, potentially offering critical diagnostic information while mitigating the risks associated with contrast agent use. Moreover, IVIM has the added benefit of obtaining matched microstructural/cellular information simultaneously. Finally, IVIM offers a complementary view of microcirculation (vascular, tubular, and others), as summarized below.

Similar to IVIM, arterial spin labeling (ASL), dynamic susceptibility contrast (DSC), and dynamic contrast‐enhanced (DCE) MRI all probe perfusion. Relationships between the IVIM pseudo‐diffusion parameters and blood flow parameters from other MRI perfusion methods have been derived [115]. A majority of the published literature found a good correlation between *f* and perfusion blood volumes derived from other perfusion MRI approaches [116]. However, as the mechanisms and biophysics behind IVIM differ from ASL, DSC, and DCE, IVIM may offer complementary information and could provide added value to the alternatives in detecting certain pathologies [117, 118, 119]. Perfusion measures from DSC and DCE are based on spatio‐temporal contrast agent kinetics, which allow for probing exchange parameters. However, unlike IVIM, they strongly depend on the accurate measurement of the contrast agent in the supplying artery, including assessment of the arterial input function. ASL uses arterial blood water as an endogenous tracer by saturating or inverting its magnetization upstream of the microvascular network [120]. Consequently, direct comparison with IVIM can be subtle, as ASL monitors the transit of externally labeled blood, while IVIM reflects all randomly moving intravoxel blood. Hybrid ASL‐IVIM methodologies have also been demonstrated that allow emphasis or suppression of different vessel sizes [121]. While these perfusion measurement techniques are interrelated, IVIM offers a distinctive advantage by providing a simultaneous assessment of tissue microcirculation (reflecting microperfusion effects) and tissue microstructure without the need for contrast agents.

IVIM parameters have been shown to be sensitive to physiological changes in perfusion, for example, increasing *D*, *f*, and D* values have been reported in muscles after exercise [67], and increased *f* in kidney and brain in cardiac systole, or in the brain during specific tasks [48, 116]. In correlation with histology, literature results demonstrate fair to good correlation between *f* and mean vessel density [116, 122]. IVIM has also been related to standard perfusion parameters, with a linear relationship between *f* and blood volume, D* and mean transit time, and fD* with blood flow [115]; however, the exact relationship between IVIM parameters and perfusion (i.e., blood flow, blood speed, and structure of the micro‐vascular network) remains to be investigated in future research.

We encourage further research on IVIM fitting alternatives. Estimator performance can be strongly influenced by acquired *b*‐values [123, 124], data quality, and design features (for instance Bayesian prior [125, 126]). Specific examples and use cases of deep learning have received increasing attention and recognition in recent years as a promising fitting alternative. Both voxel‐wise [93, 94, 127] and spatially‐aware [128, 129, 130] deep learning methodologies show potential for improving estimator accuracy and reducing computation time. However, it is essential to approach deep learning supported IVIM modeling with caution and ensure comprehensive validation [131].

While many comparative studies exist for subsets of IVIM‐fitting methods [125, 126, 131, 132, 133, 134], a major limiting factor in the identification of a new gold‐standard estimator is the lack of standardized tools for the characterization of estimator performance. Quantitative assessment of accuracy and bias is only possible using synthetic data, whereas performance on in vivo data can only be assessed either in terms of precision (repeatability [133, 135]) or classification accuracy (for instance, tumor type [136]). Hence, there is a need for reliable validation and open‐source benchmarks, such as realistic digital IVIM phantoms, standardized synthetic data sets, freely‐available in vivo datasets (with multiple repetitions) for various organs and pathologies, ideally complemented by histological characterization, and physical IVIM phantoms. We also need consensus on standardized metrics for evaluating performance. We encourage the development of international IVIM fitting competitions (“challenges”) as a gold standard for fair performance evaluation enabling algorithmic benchmarking in a controlled environment while simulating real‐world conditions. We support increased implementation of inline IVIM processing by MRI system vendors, not only conventional simultaneous fitting but also segmented variants.

We encourage research into advanced variants of IVIM that may eventually be translated as broadly as the canonical variant. As the tissue and vessel structure is very complex, several extensions to the standard IVIM model have been proposed. First, the microstructural compartment of the signal only shows Gaussian diffusion behavior (as in the canonical IVIM variant) to first order, and deviations from this single exponential decay arise at higher *b*‐values (typically exceeding *b* = 1000 s/mm^2^). Diffusion kurtosis imaging (DKI) provides one quantification of this non‐Gaussian behavior, and hybrid IVIM/DKI methods have been shown to prevent confounds between their respective imaging biomarkers [137, 138]. Multi‐compartment behavior that expands IVIM beyond the standard two‐compartment model has shown promise in organs with physiologic compartments with distinct diffusion rates. This includes tri‐exponential and spectral diffusion in the kidney (for vascular and tubular flow) [105, 139, 140, 141, 142, 143, 144] and the brain [145, 146, 147].

A key assumption of the IVIM model is that blood flowing through randomly oriented capillary segments induces a phase dispersion of the MR signal, leading to signal attenuation in diffusion‐weighted images. However, most body tissues have a capillary network with an anisotropic microarchitecture. Several advanced diffusion models combining the IVIM theory with DTI formalism have been proposed to characterize the anisotropy of diffusion and the flow‐related signal in the kidney [48, 148, 149, 150], skeletal muscles [151], myocardium [152], and cerebral gray matter [146, 153]. Overall, it has been demonstrated that the IVIM signal in these tissues is direction‐dependent, suggesting that both the tissue microstructure and (blood) flow contribute to the diffusion anisotropy in tissues with a spatially organized capillary network.

Variation of diffusion time is known to modulate both aspects of the IVIM signal (microcirculation and microstructure), and the microcirculation aspect has been recognized in multiple contexts [107, 108, 154] as the water motion shows a ballistic (short‐time), diffusive (long‐time), or intermediate regime. This modulation can be a route to more specific tissue modeling and biomarker quantification.

Another experimental tool contrast for enhanced quantification is the modulation of the diffusion gradient waveform. Through the first moment (M1) of the gradient waveform, the degree of flow‐induced signal decay can be altered, up to the extreme case of a fully flow‐compensated (FC) (M1 = 0) waveform, which will nullify all signal decay from flow that remains steady during the echo time TE. Schemes have thus been offered to continuously vary M1 to generate a more precise or more complete characterization of IVIM contrast [68, 107].

Since physiologic vasculature occurs with a range of flow speeds and channel geometry, the flow compensation condition is variably met throughout the tissue, and thus variation of either flow encoding moment and diffusion time (or both) can be advantageous to capture the full network behavior. In that sense, flow‐compensated DWI for IVIM modeling (FC‐IVIM) provides a more comprehensive description of tissue microvasculature than conventional IVIM [48, 150, 155, 156, 157]. Beyond evaluating *D* and *f*, FC‐IVIM additionally incorporates microvascular characteristics by modeling blood flow velocity (*v*) and vessel segment length (*l*), which are related via a characteristic time scale *τ* = *l*/*v*. It is believed that these additional flow‐related parameters could be used as markers to reveal pathological changes in microvasculature associated with various diseases. In addition, flow‐compensated diffusion gradients were found to reduce motion‐related artifacts in DWI of the liver [158, 159], pancreas [160], and kidney [84].

The standard IVIM model does not account for the different transverse relaxation times (T_2_) of tissue, blood, and other compartments. This results in the overestimation of *f*, particularly as TE and T_2_ weighting increase. Jerome et al. [103] proposed an extended model called the T2‐IVIM model, which enables measurement of the T_2_ values for both compartments (tissue and blood). This model, which requires sampling multiple *b*‐values at multiple echo times, corrects the TE dependence of the *f* parameter, leading to more accurate parameter estimates. Moreover, using multiple relaxation weightings may enhance our understanding of the different compartments contributing to the IVIM signal decay.

## 6 Limitations

Note that this paper reflects a consensus, which is not necessarily the same as stating these are the “optimal” settings. For example, the simulations to determine best cut‐off values for segmented fit were based on a heuristic rule that *e* ^−bD^* ~ 0.05 and then rounded to some shared cut‐off. This paper does not aim to describe universally “optimal” settings (as such a concept is elusive given all organ contexts considered) but aims to harmonize protocols for more reproducible results. We believe that at this stage, harmonization will be an important step to bring IVIM closer to the clinic. We acknowledge that IVIM is still an active research topic, and some future insights may alter the recommendations, which may then be updated accordingly by an analogous process as performed here.

## 7 Conclusions

We present consensus‐driven recommendations on how to perform IVIM by target anatomy, covering all major organ systems. We provide context for these recommendations from the existing IVIM literature and highlight the most compelling scientific frontiers of IVIM development and application. We hope that the broad implementation of these recommendations will make IVIM more accessible to clinical and technical researchers and accelerate clinical translation of IVIM. In particular, it can contribute to building an evidence base conducive to multi‐site translation and data pooling and feed into societal recommendations, approvals by national and international health authorities, and clinical trial usage. We expect that the best utilization of IVIM continues to promote and leverage multidisciplinary dialogue that formed the basis of this consensus.

## Funding

This work was supported by National Institutes of Health (K99DK138294, P30 CA008748, R01CA245671, R01DK125561, R01DK136983, TL1TR004420, UH3CA239861); Japan Agency for Medical Research and Development (23ck0106892h0001, 23he0422025j0002); Deutsche Forschungsgemeinschaft (437119659); Cancer Research UK (C33589/A28284); Nederlandse Organisatie voor Wetenschappelijk Onderzoek (NWA.1418.22.002); KWF Kankerbestrijding (KWF‐UvA 2021.13785); Swedish state under the agreement between the Swedish government and the county councils, the ALF‐agreement (ALFGBG‐942664); Breast Cancer Research Foundation and the Image Processing & Analysis Core (iPAC) at the University of Massachusetts Chan Medical School. P.T. While gratefully acknowledges support from the Research Council of Norway under FRIPRO Researcher Project 302624.

## Supporting information


**Supplementary Information 1** Survey Part 1.

**Supplementary Information 2** Survey Part 2.

**Supplementary Information 3** Workshop Polls.

**Supplementary Information 4** Recommendations.

**Supplementary Information 5** Estimating *f* and S0.

**Supplementary Information 6** Reporting checklist.

**Supplementary Information 7** Test–retest studies.
