# Supplementary material for: Towards Clinical Translation of Intravoxel Incoherent Motion MRI: Acquisition and Analysis Consensus Recommendations
Source: J Magn Reson Imaging. 2026 Mar 19;63(6):1782–801. doi: 10.1002/jmri.70278 (PMC13175230; doi:10.1002/jmri.70278)
Supplement: Supplementary file 1 — Supplementary Information 1 Survey Part 1. [file JMRI-63-1782-s003.pdf]

Supplemental Information 1: Survey Part 1

Belonging to:
“Towards Clinical Translation of Intravoxel Incoherent Motion MRI: Acquisition and Analysis Consensus Recommendations”, *JMRI*, Sigmund et al.

Overview Survey Part 1 Questions

1. What is your training?
2. What target system are you working on?
3. What applications are you working on?
   IVIM applications
   For each application listed below, the question was as follows:
   a. What is the confidence level to use IVIM on a scale from 1 (should not be used) to 10 (should be used in a daily clinic with no further proof needed)? Note that a score of 0 reflects abstaining from voting. This reflects, for example, body experts who do not have a strong opinion on brain confidence.
   b. Which IVIM parameters should be analyzed?
4. Neuro: Stroke
   c. Stroke targets
5. Neuro: Degenerative disease
6. Neuro: Tumors
7. Neuro: Cerebral death
8. Body: Liver cirrhosis / fibrosis
9. Body: Chronic kidney disease
10. Body: Acute kidney injury
11. Body: Tumors
12. MSK: Muscular dystrophy
13. MSK: Inflammatory myopathies
14. MSK: Functional muscle imaging
15. Oncology: Tumor detection
16. Oncology: Tumor grading
17. Oncology: Tumor prognostic factors / characterization
18. Oncology: Tumor treatment response monitoring
19. Oncology: Tumor treatment response prediction
20. Fetal / placental imaging
21. Cardiac imaging
    IVIM to replace contrast injection
22. Do you think IVIM could replace contrast injection for specific pathologies?
23. Do you think IVIM could replace contrast injection in patients with contrast contra-indications?
    Specific comments
24. Neuro indications
25. Body indications
26. MSK indications
27. Oncology indications
28. Fetal / placental imaging
29. IVIM for perfusion
30. Other

Supplemental Information to “Towards Clinical Translation of Intravoxel Incoherent Motion MRI: Acquisition and Analysis Consensus Recommendations” by Sigmund et al.

1.

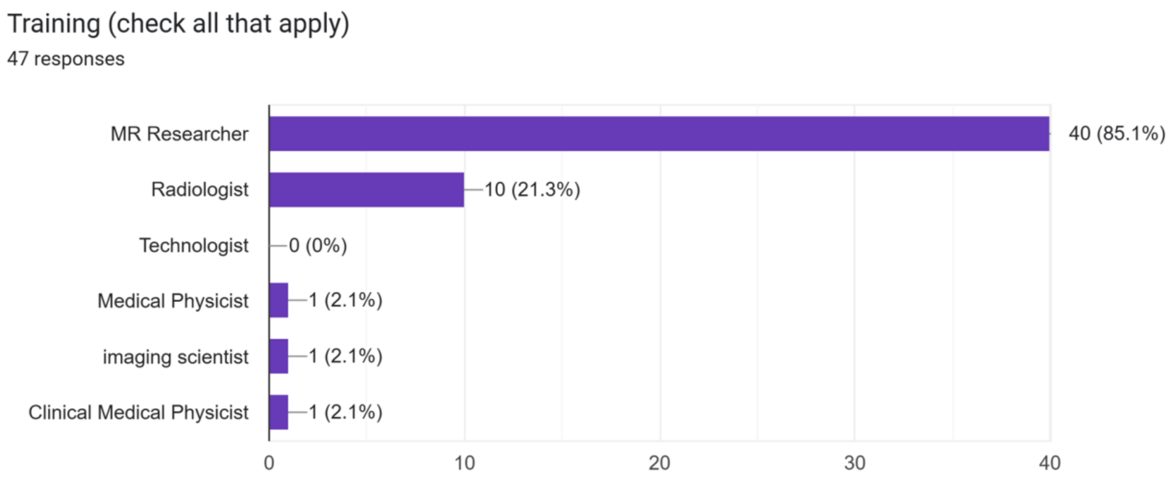

2.

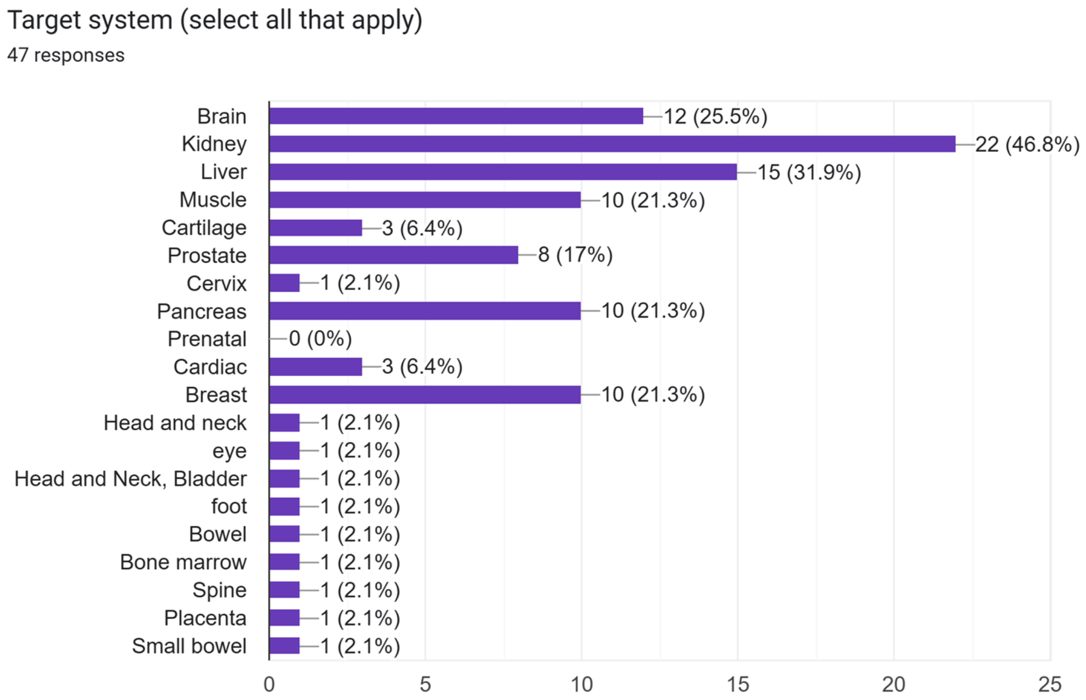

3.

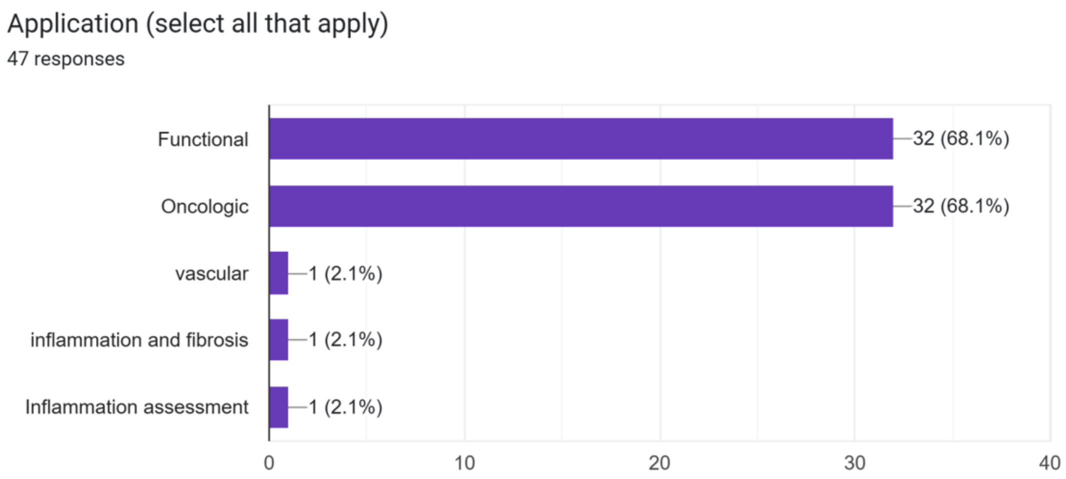
Application (select all that apply)
47 responses
Functional
32 (68.1%)
Oncologic
32 (68.1%)
vascular
1 (2.1%)
inflammation and fibrosis
1 (2.1%)
Inflammation assessment
1 (2.1%)
0
10
20
30
40

Applications

4. Neuro: stroke

a) Confidence level

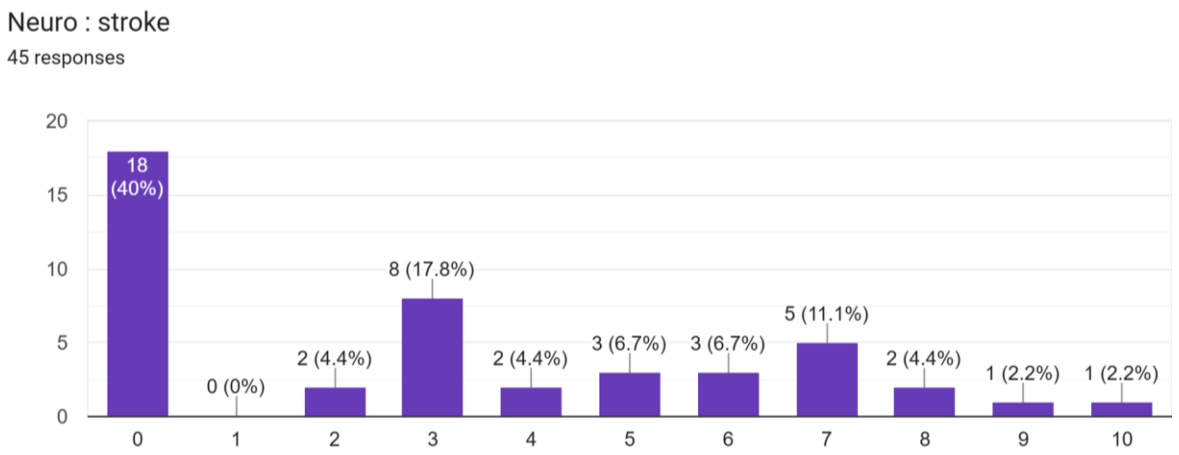

b) IVIM parameters to analyze

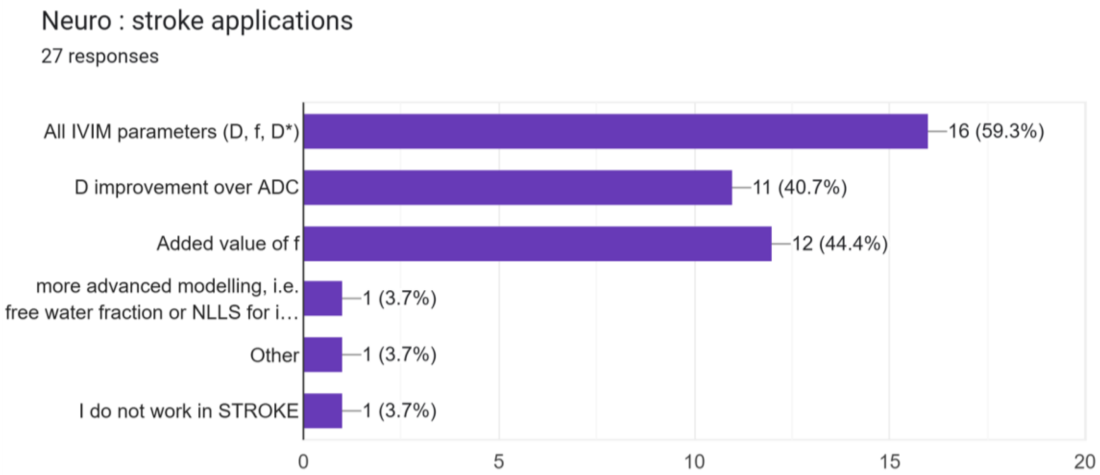

c)

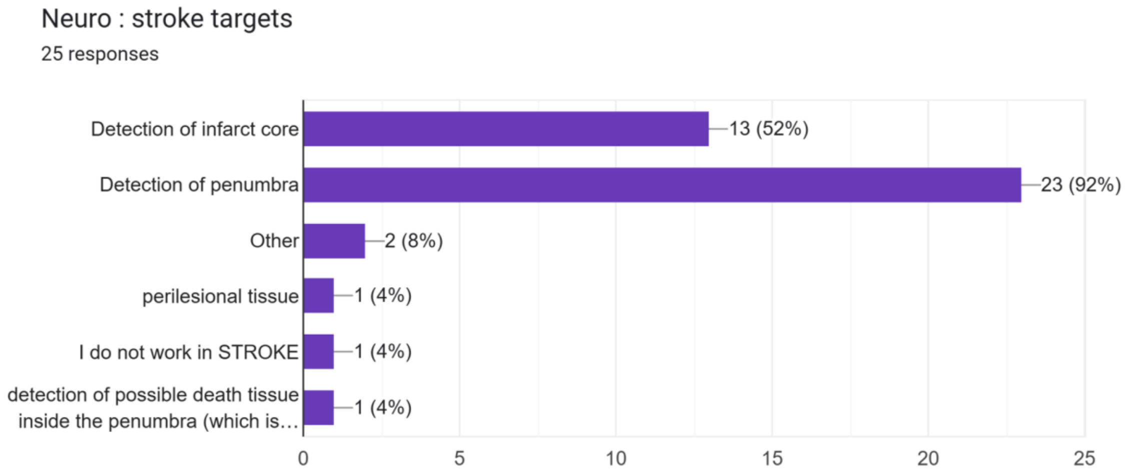

5. Neuro: degenerative disease

a) Confidence level

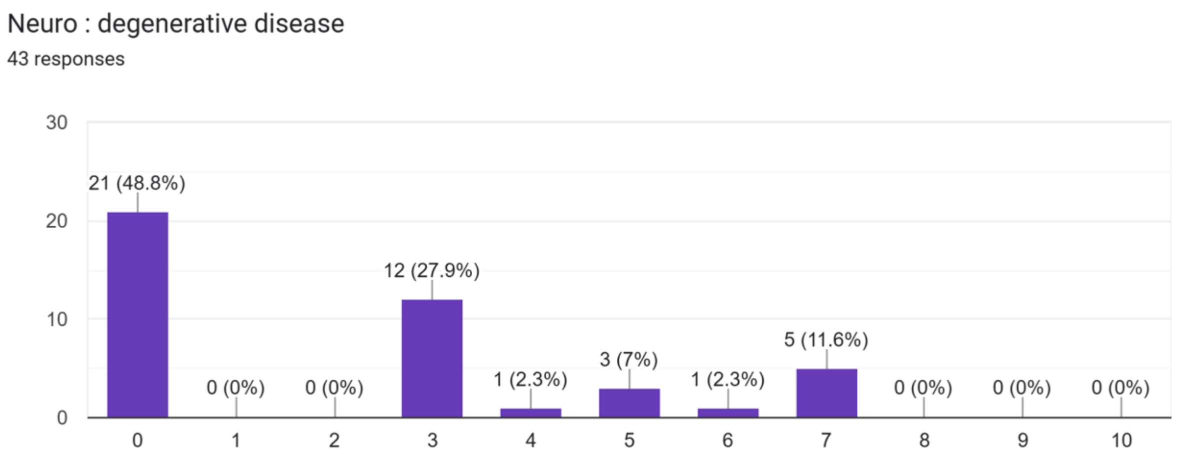

b) IVIM parameters to analyze

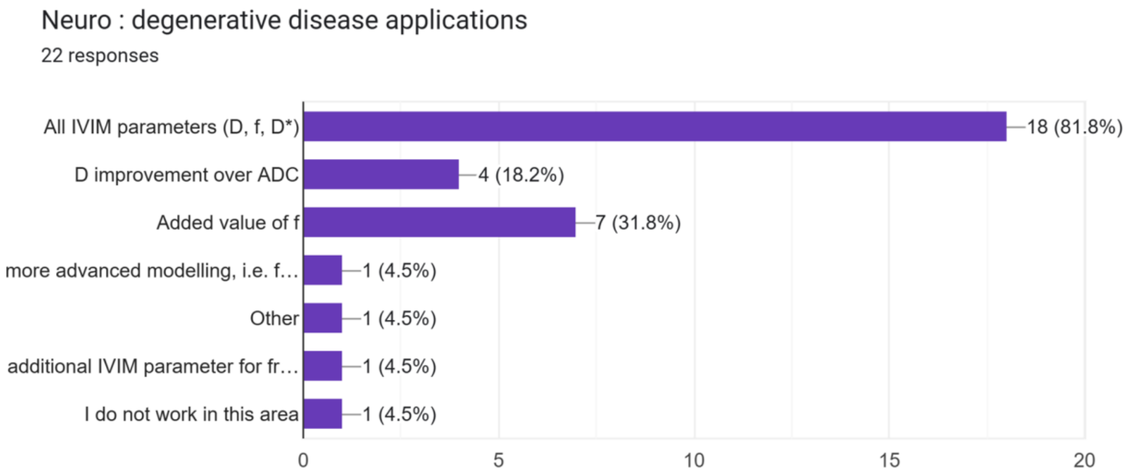

6. Neuro: tumors

a) Confidence level

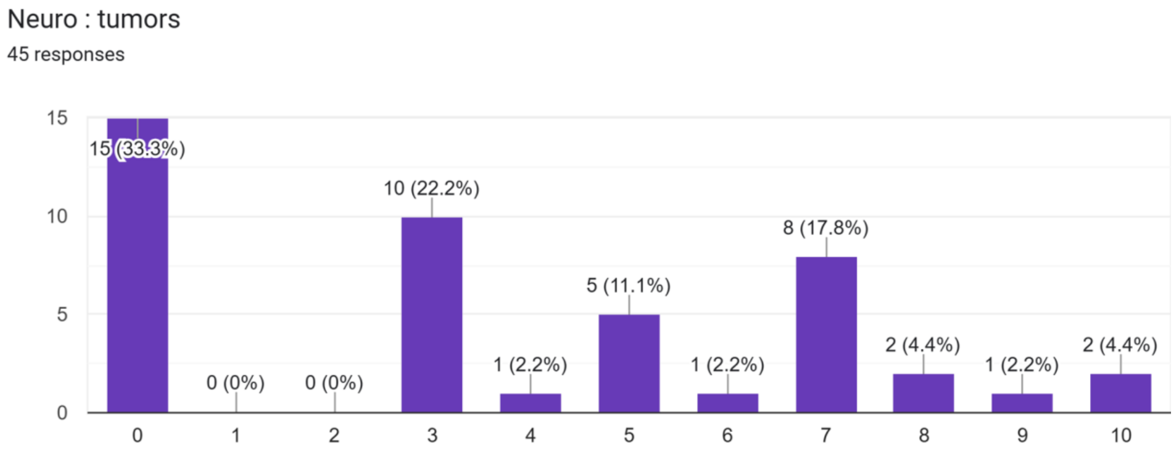

b) IVIM parameters to analyze

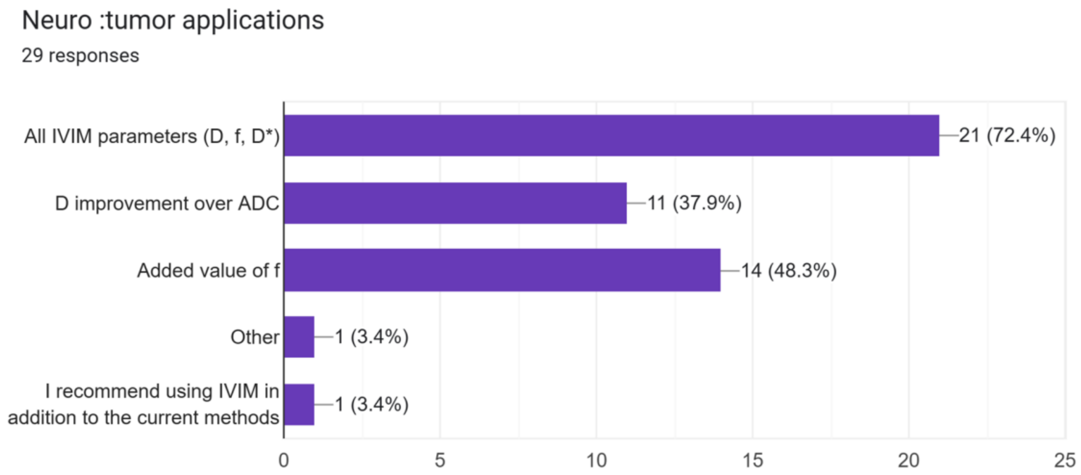

7. Neuro: cerebral death

a) Confidence level

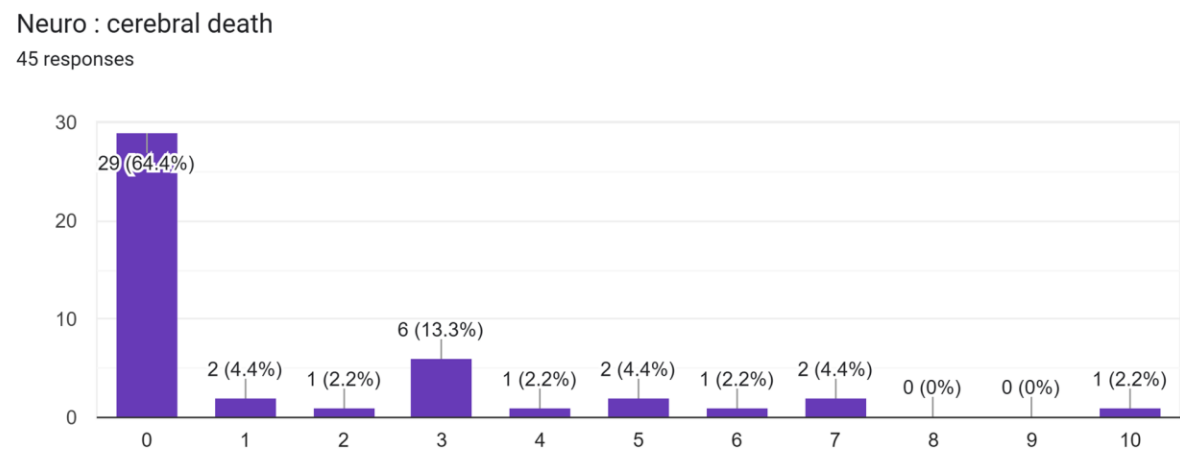

b) IVIM parameters to analyze

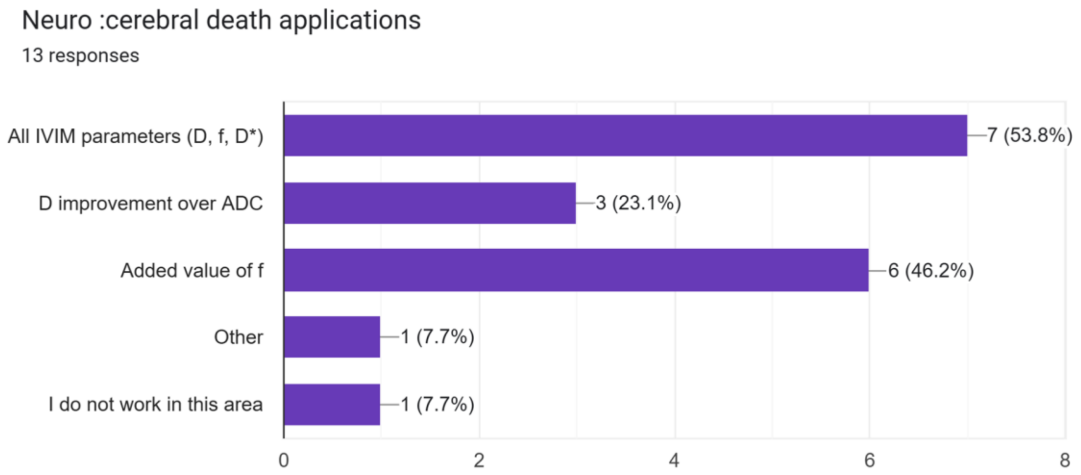

8. Body: liver cirrhosis / fibrosis

a) Confidence level

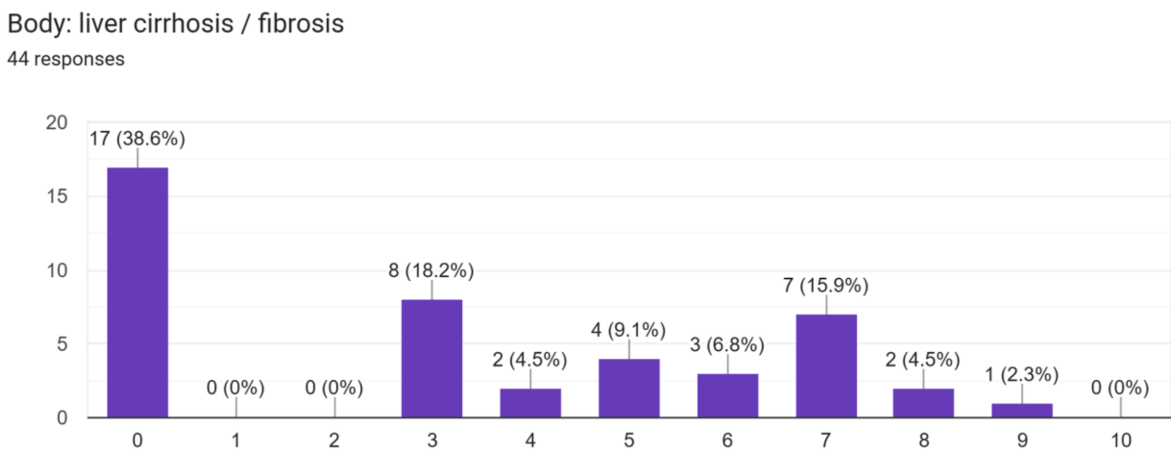

b) IVIM parameters to analyze

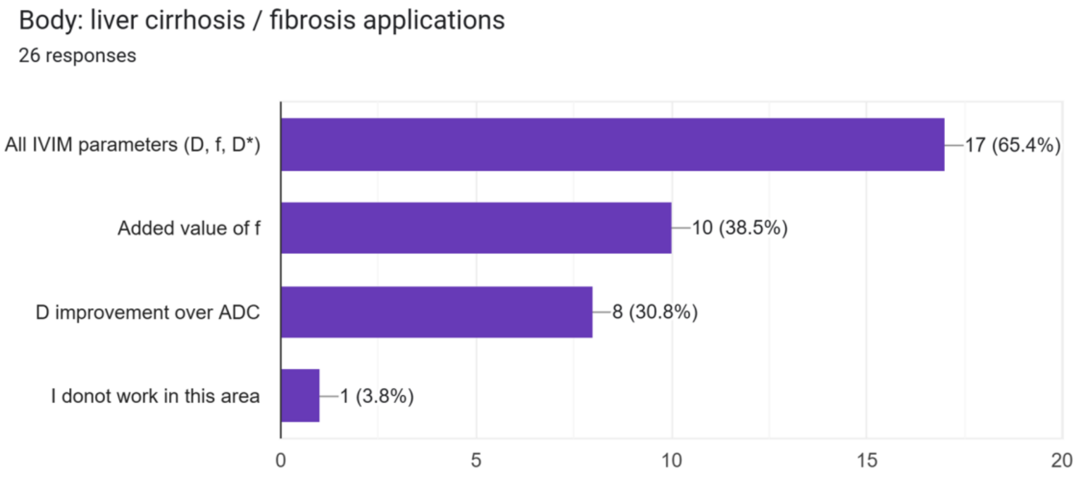

9. Body: chronic kidney disease

a) Confidence level

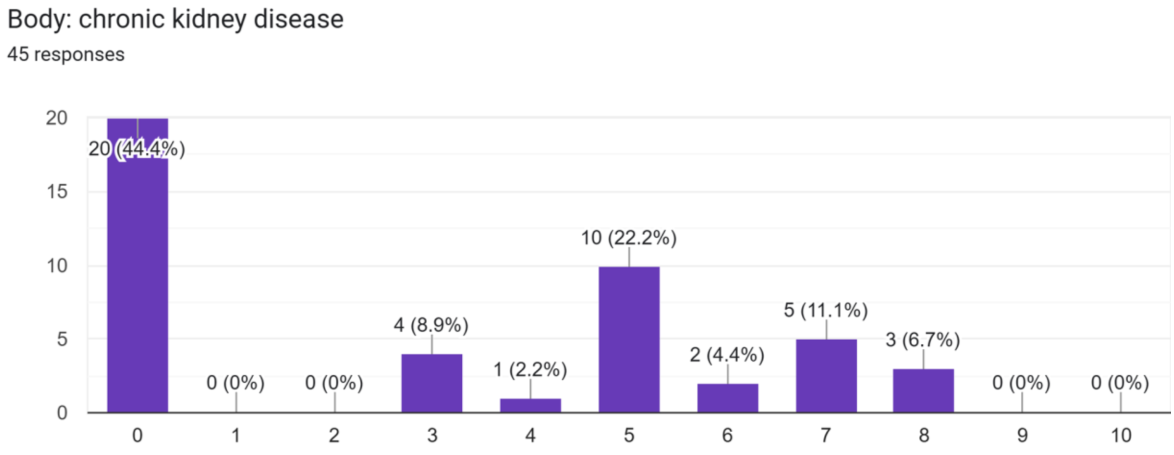

b) IVIM parameters to analyze

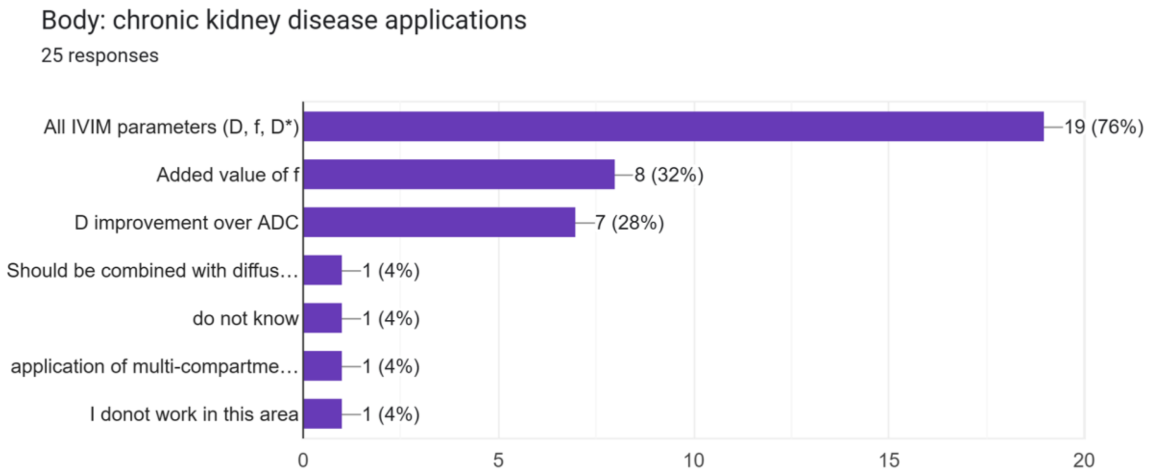

10. Body: acute kidney injury

a) Confidence level

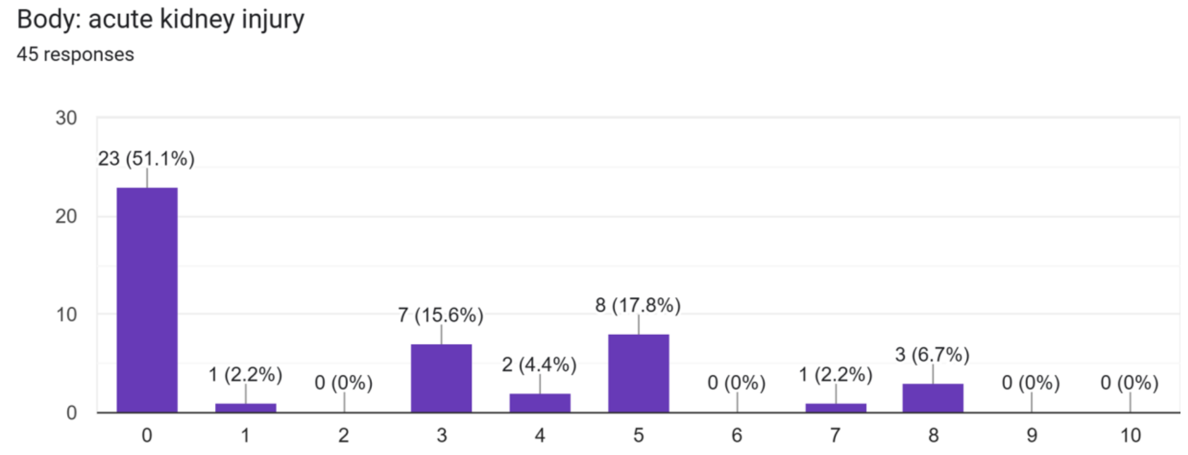

b) IVIM parameters to analyze

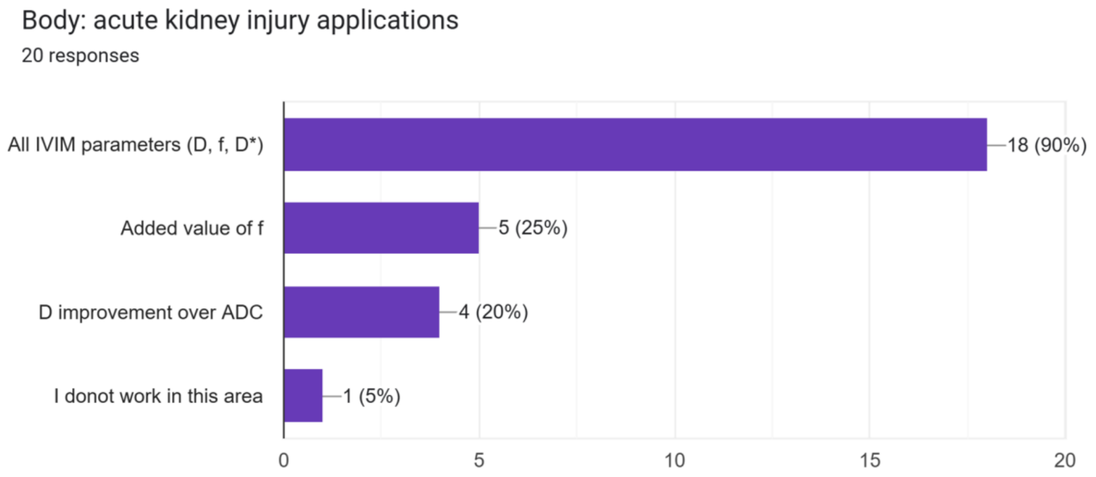

11. Body: tumors

a) Confidence level

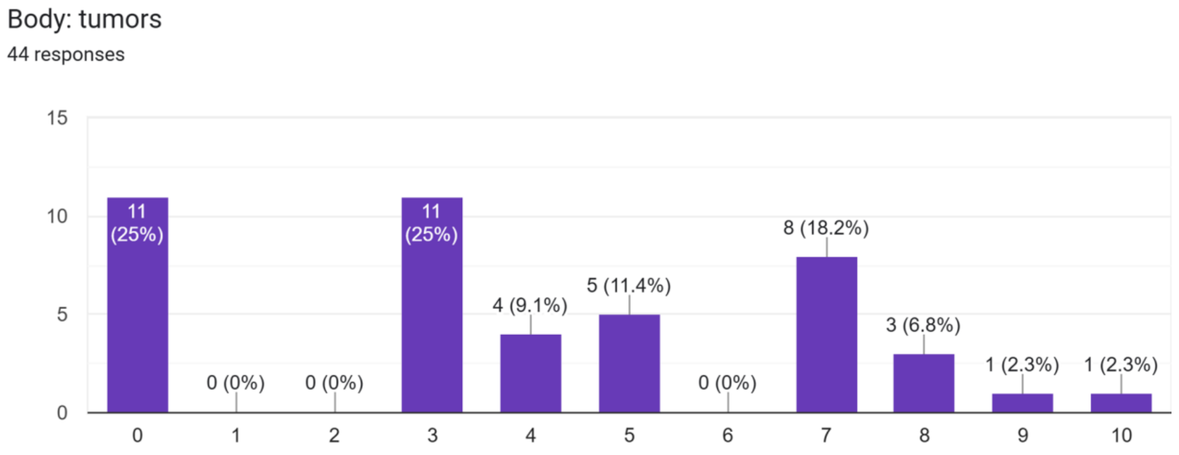

b) IVIM parameters to analyze

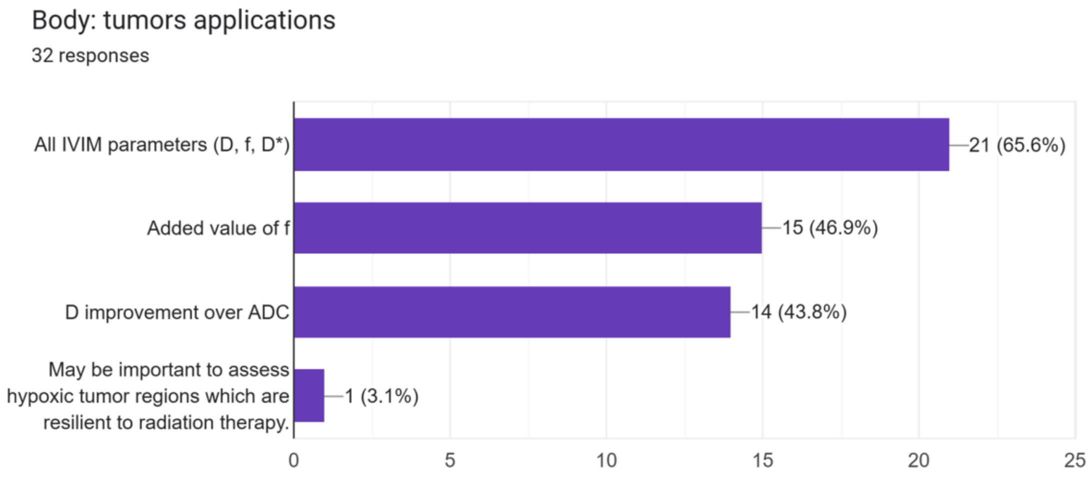

12. MSK: muscular dystrophy

a) Confidence level

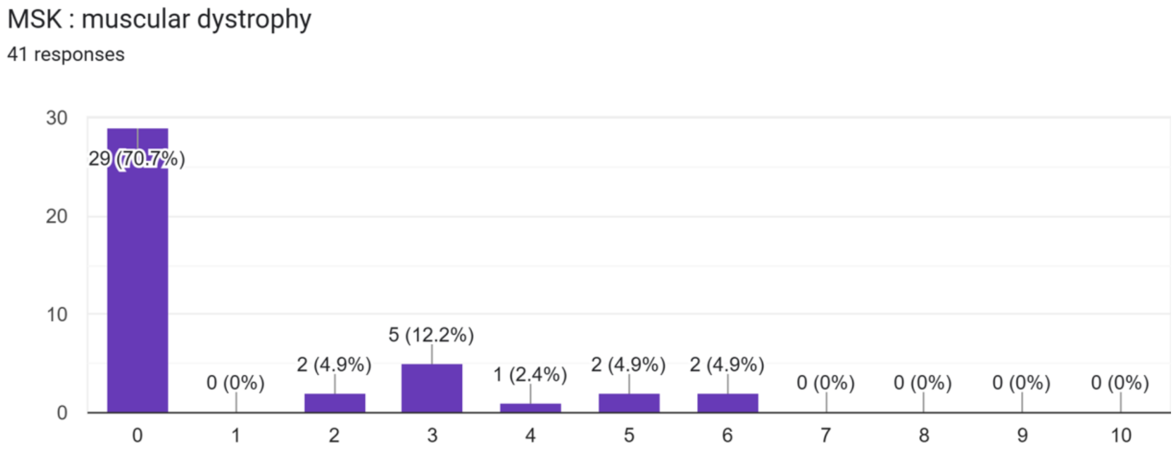

b) IVIM parameters to analyze

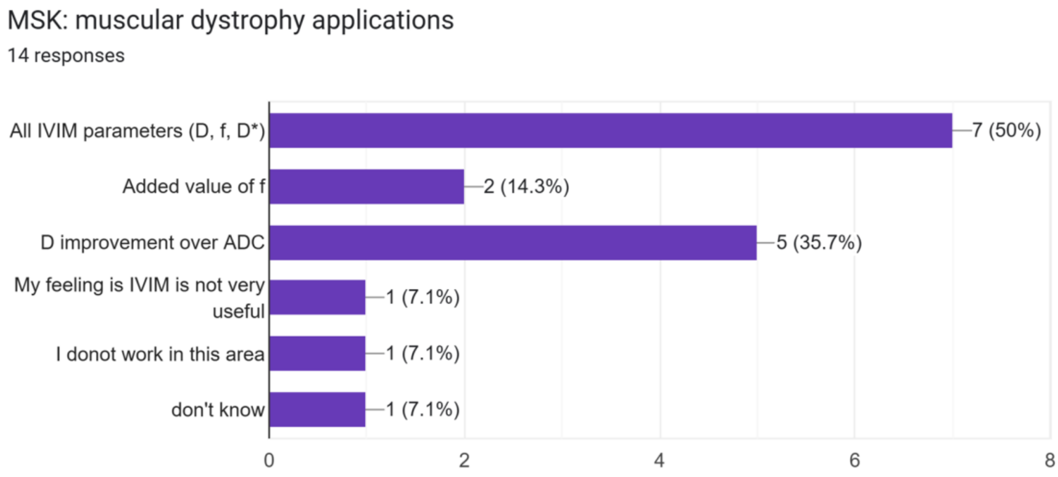

13. MSK: inflammatory myopathies

a) Confidence level

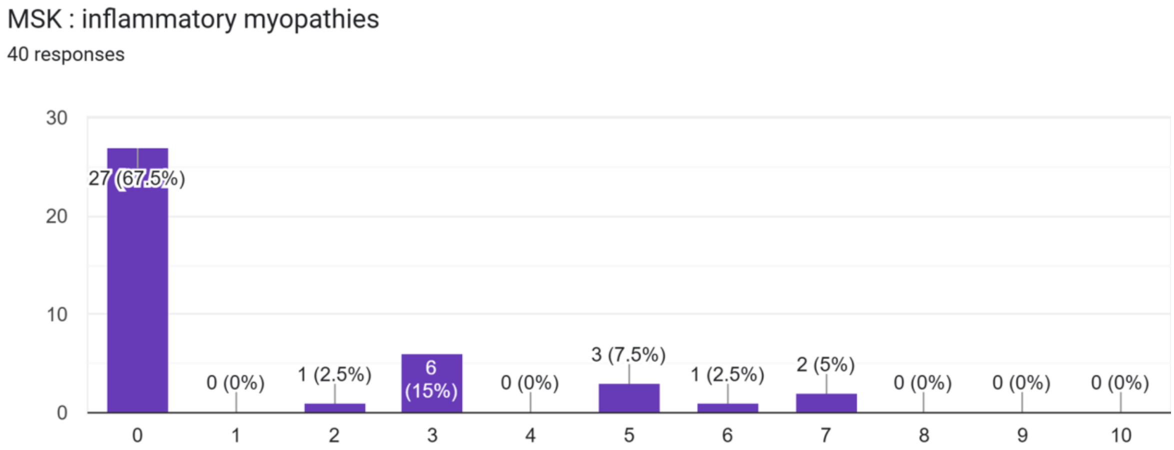

b) IVIM parameters to analyze

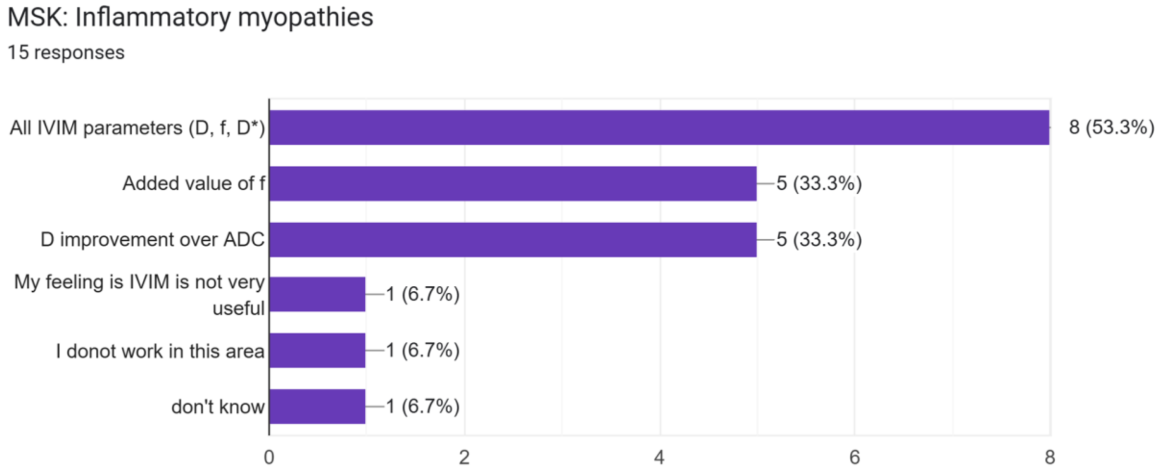

14. MSK: functional muscle imaging

a) Confidence level

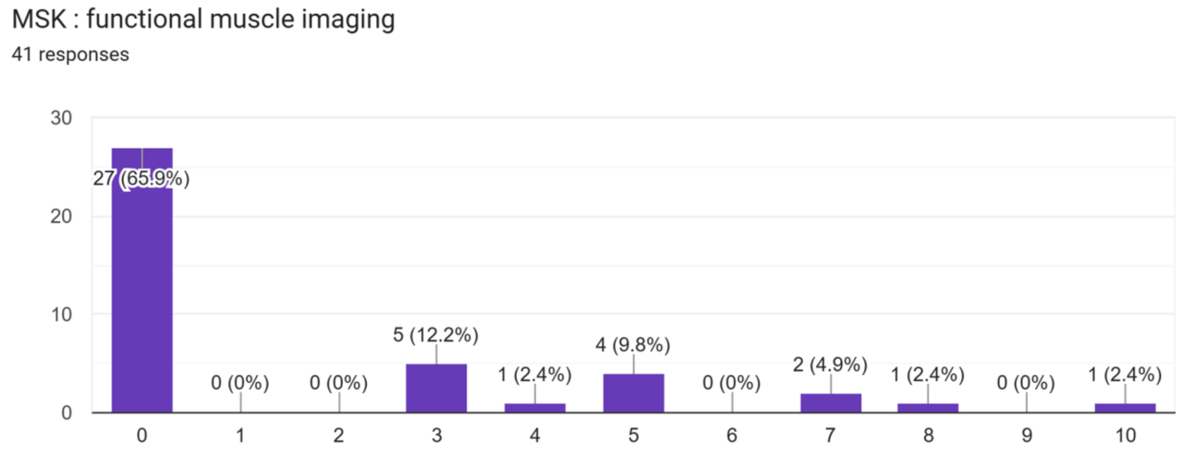

b) IVIM parameters to analyze

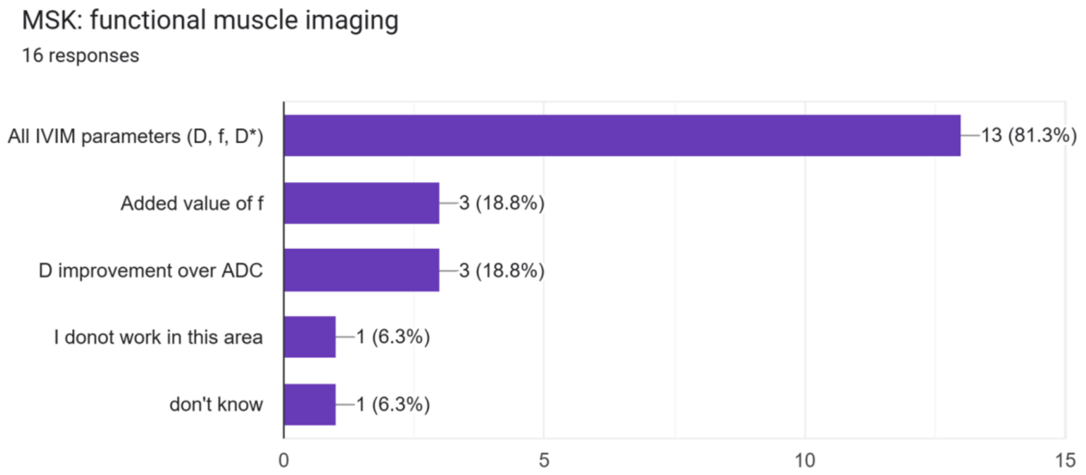

15. Oncology: tumor detection

a) Confidence level

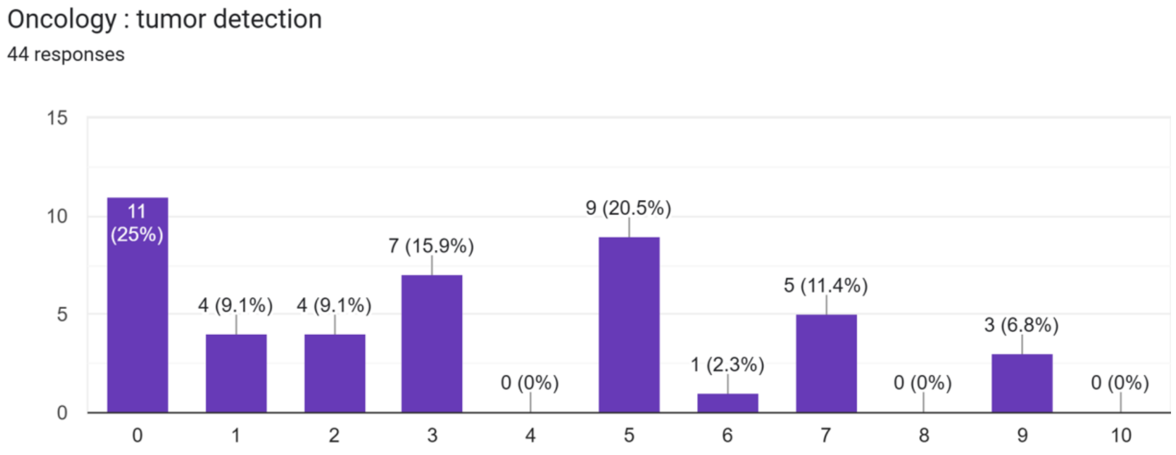

b) IVIM parameters to analyze

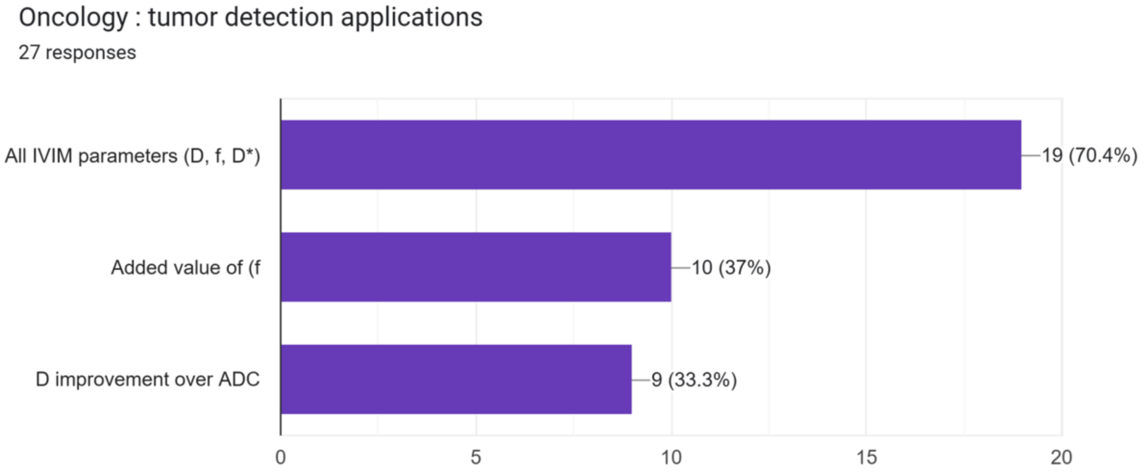

16. Oncology: tumor grading

a) Confidence level

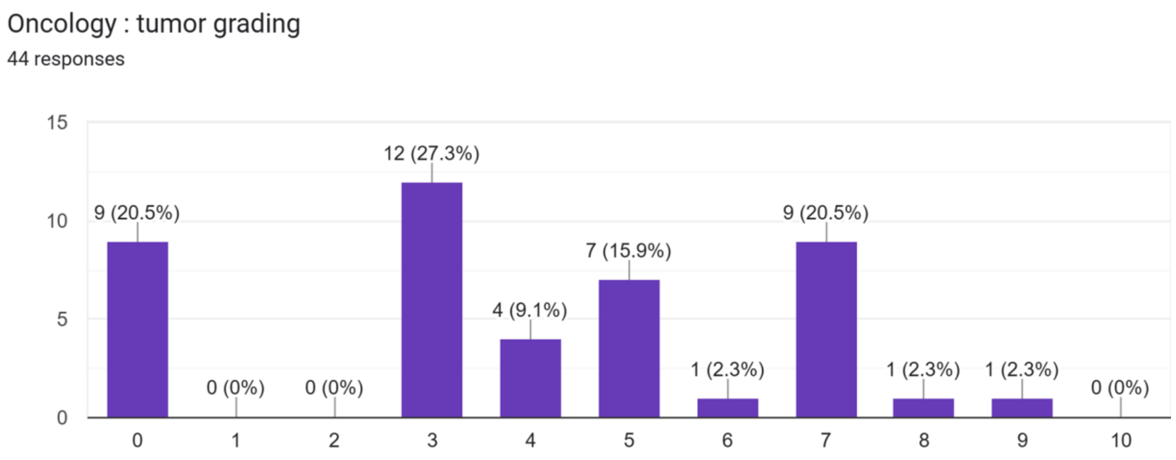

b) IVIM parameters to analyze

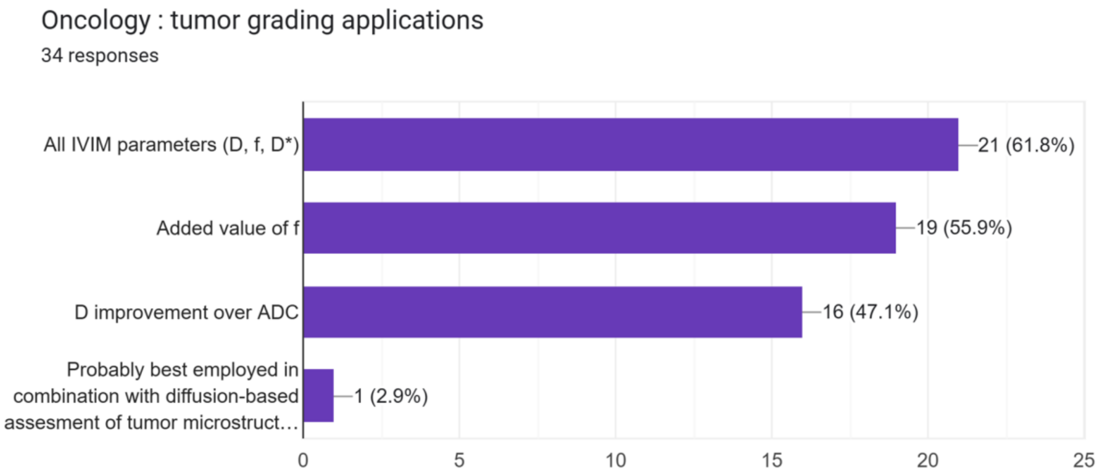

17. Oncology: tumor prognostic factors / characterization

a) Confidence level

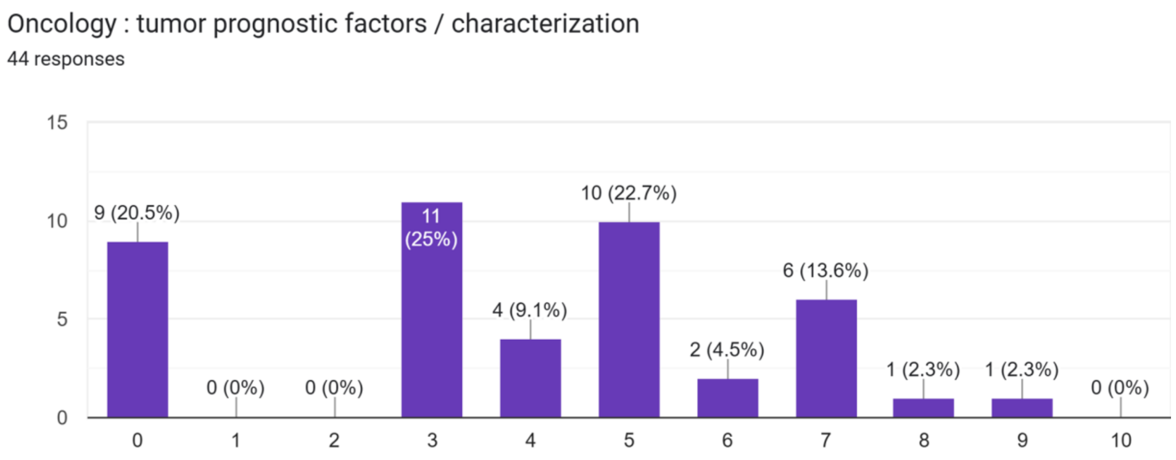

b) IVIM parameters to analyze

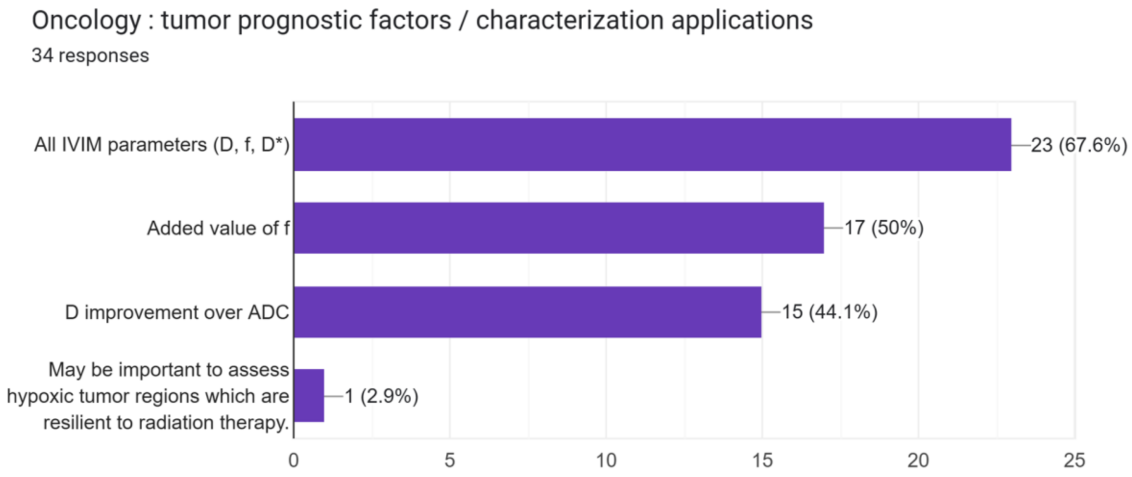

18. Oncology: tumor treatment response monitoring

a) Confidence level

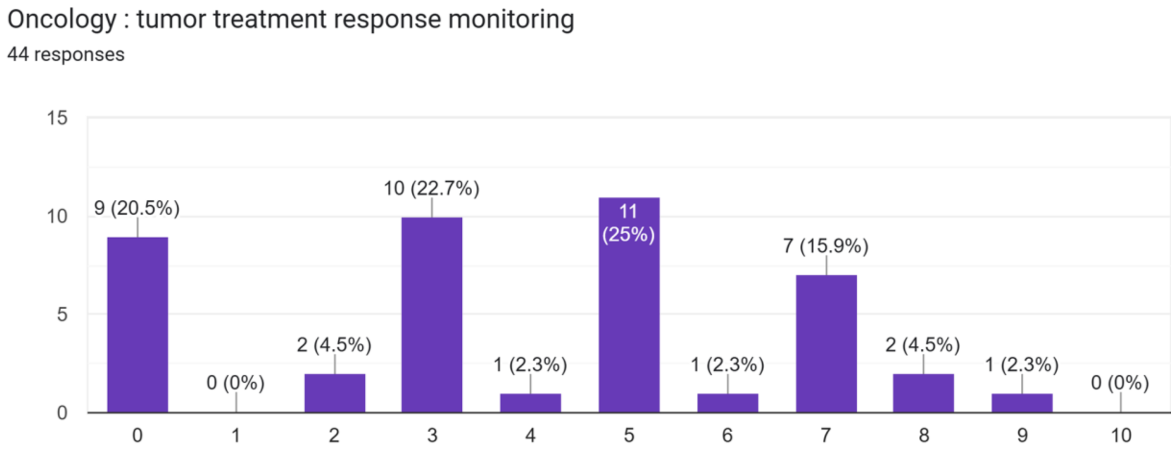

b) IVIM parameters to analyze

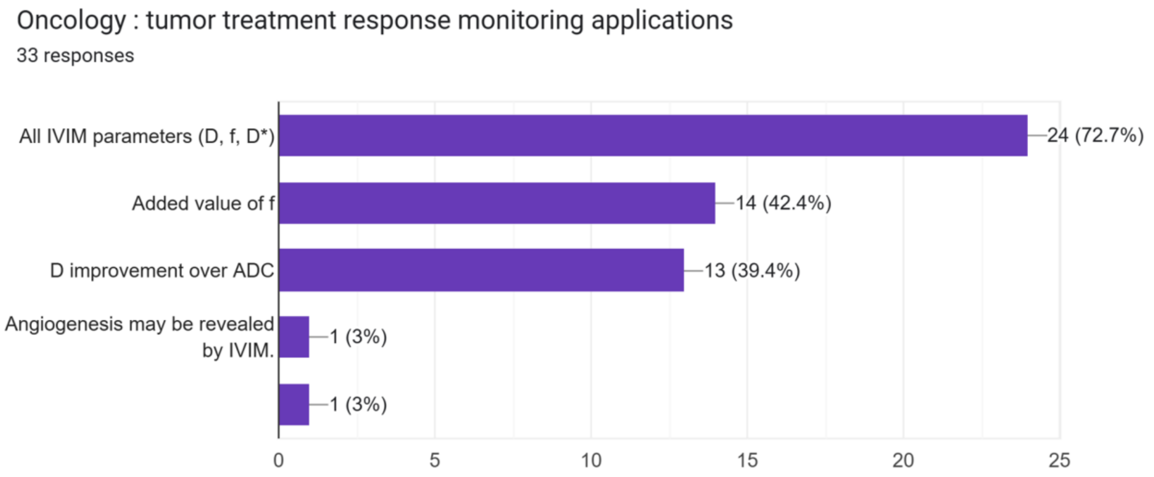

19. Oncology: tumor treatment response prediction

a) Confidence level

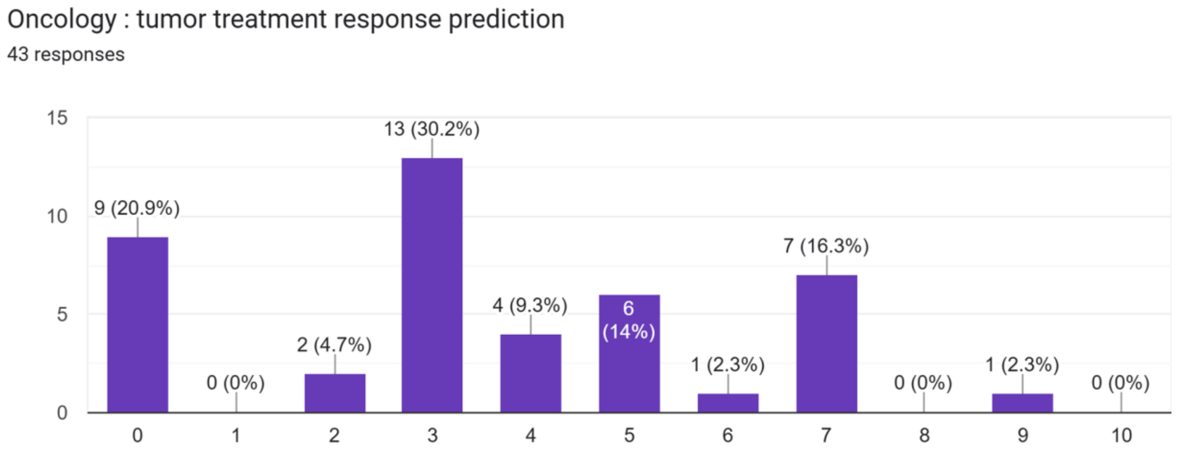

b) IVIM parameters to analyze

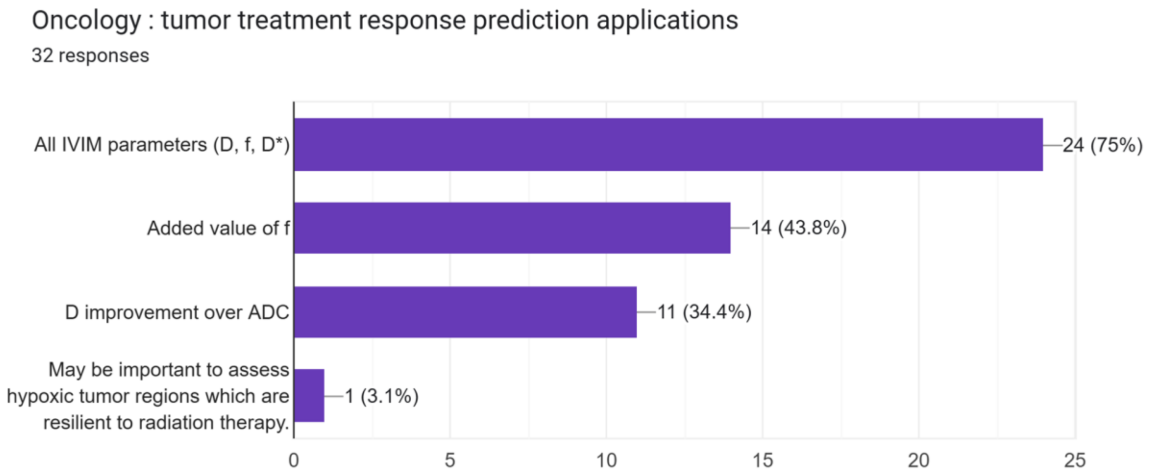

20. Fetal / placental imaging

a) Confidence level

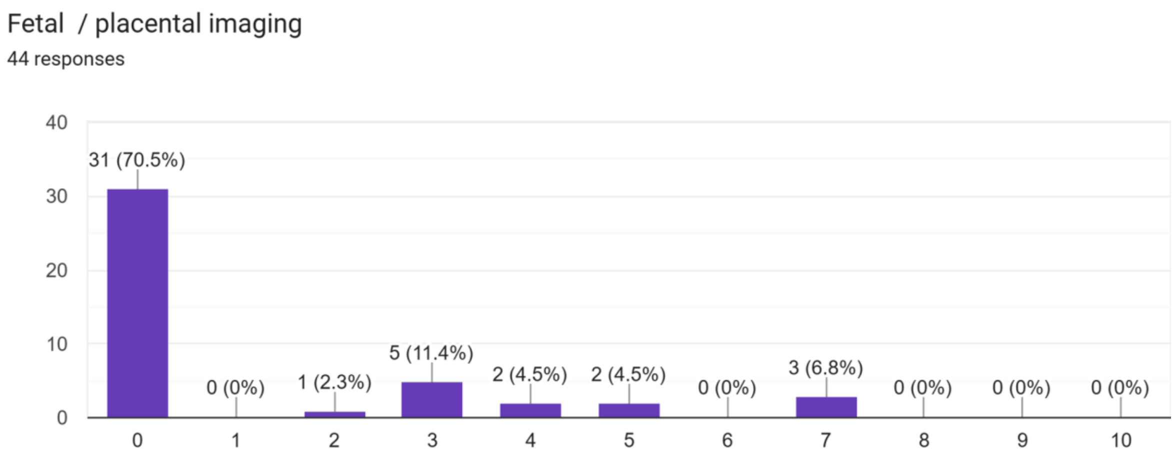

b) IVIM parameters to analyze

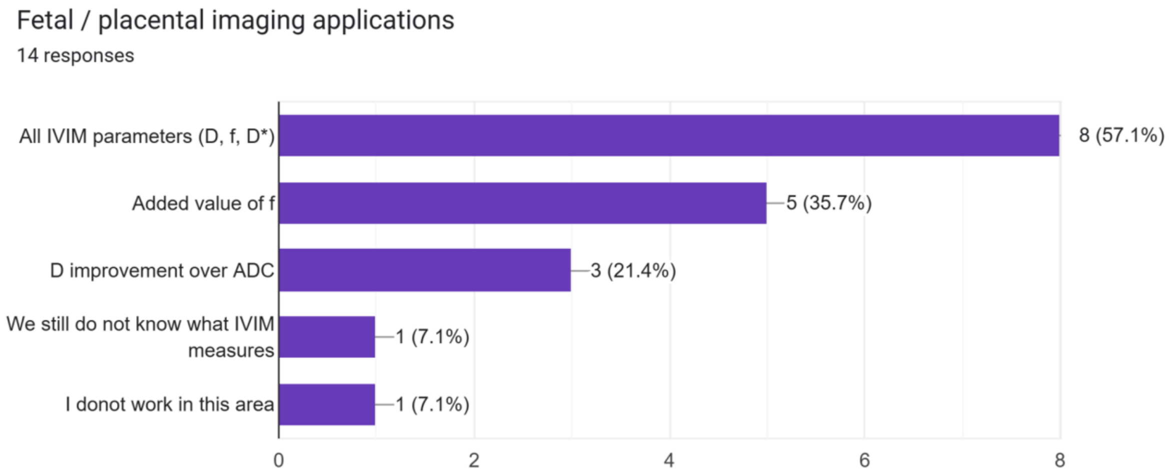

21. Cardiac imaging

a) Confidence level

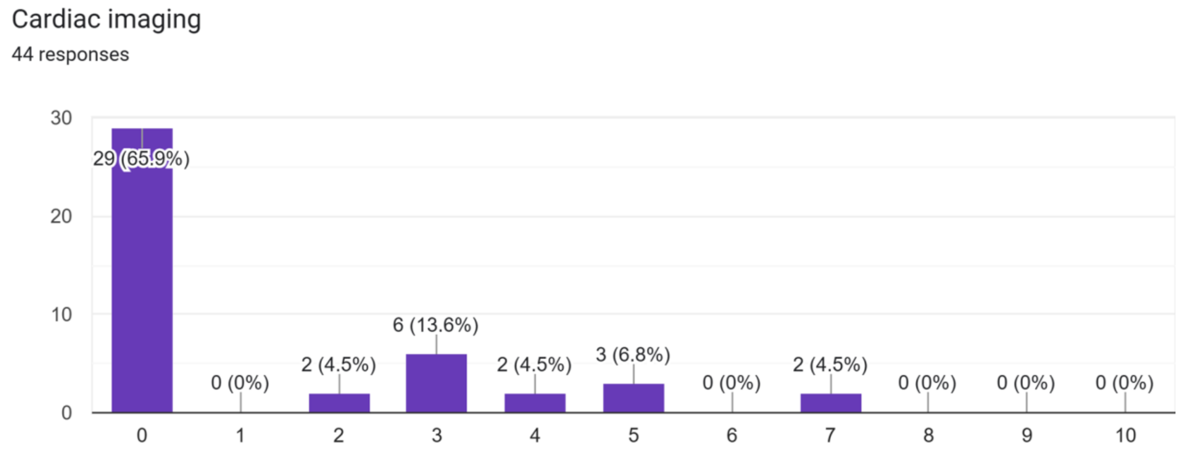

b) IVIM parameters to analyze

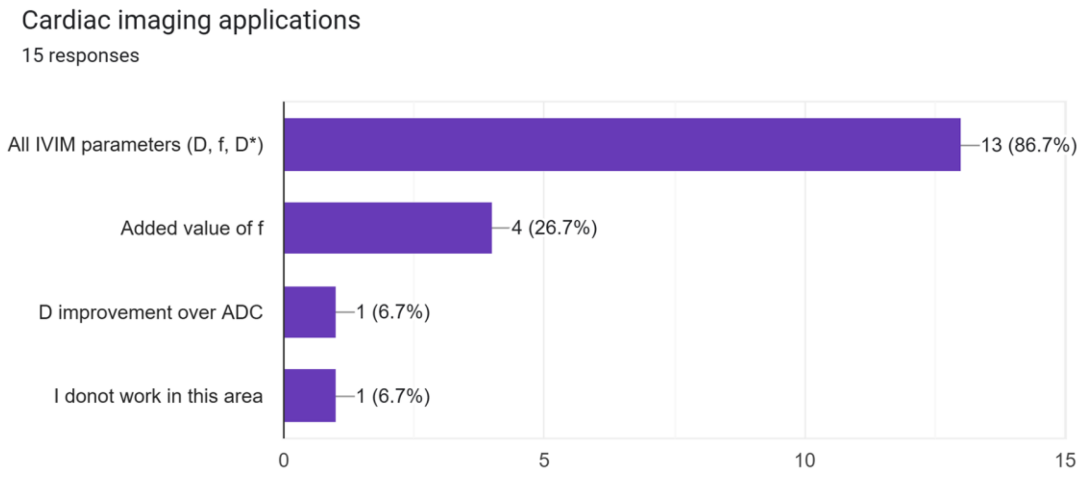

22.

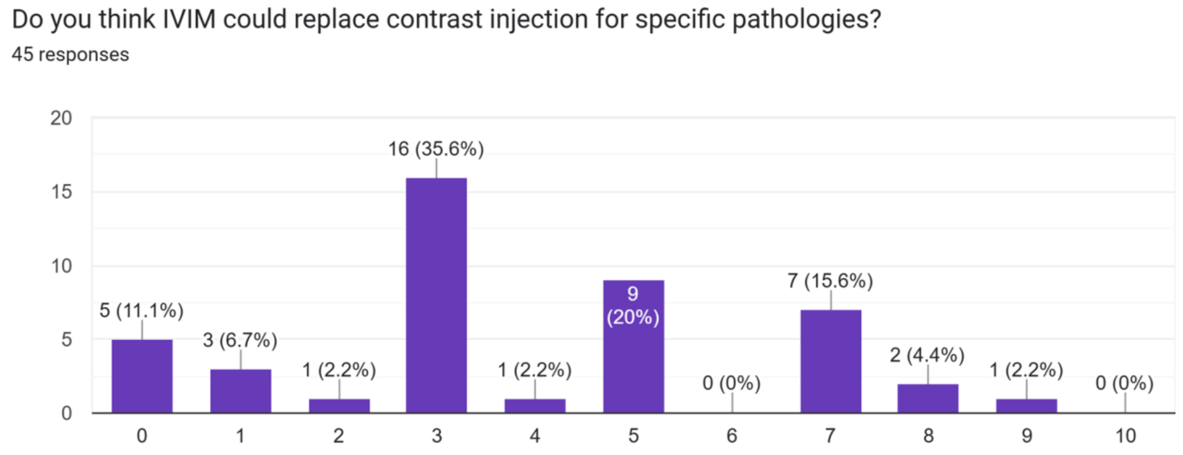
Do you think IVIM could replace contrast injection for specific pathologies?
45 responses
20
15
10
5
0
5 (11.1%)
3 (6.7%)
1 (2.2%)
16 (35.6%)
1 (2.2%)
9 (20%)
0 (0%)
7 (15.6%)
2 (4.4%)
1 (2.2%)
0 (0%)
0
1
2
3
4
5
6
7
8
9
10

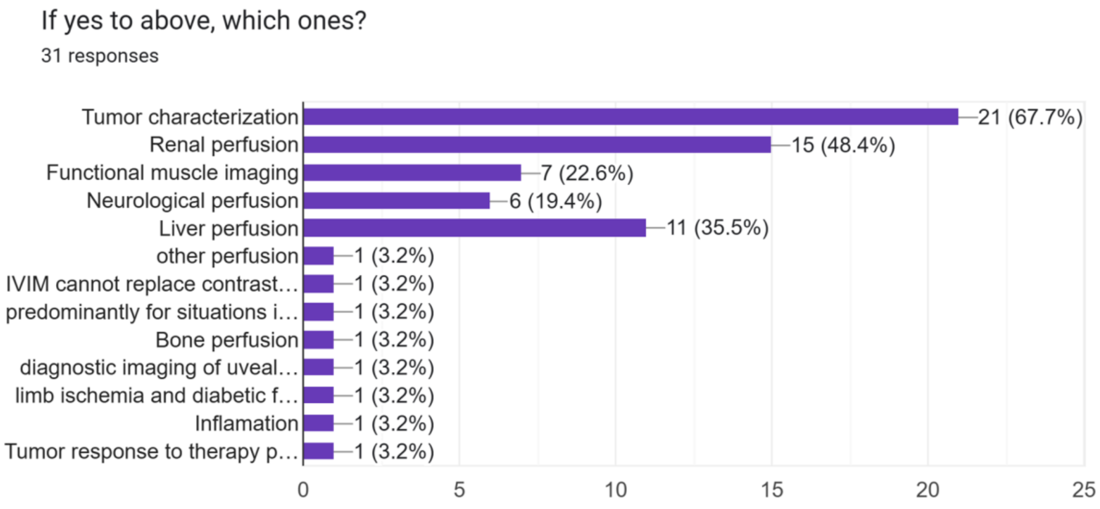
If yes to above, which ones?
31 responses
Tumor characterization
21 (67.7%)
Renal perfusion
15 (48.4%)
Functional muscle imaging
7 (22.6%)
Neurological perfusion
6 (19.4%)
Liver perfusion
11 (35.5%)
other perfusion
1 (3.2%)
IVIM cannot replace contrast…
1 (3.2%)
predominantly for situations i…
1 (3.2%)
Bone perfusion
1 (3.2%)
diagnostic imaging of uveal…
1 (3.2%)
limb ischemia and diabetic f…
1 (3.2%)
Inflamation
1 (3.2%)
Tumor response to therapy p…
1 (3.2%)
0
5
10
15
20
25

Supplemental Information to "Towards Clinical Translation of Intravoxel Incoherent Motion MRI: Acquisition and Analysis Consensus Recommendations" by Sigmund et al.

23.

Do you think IVIM could replace contrast injection in patients with contrast contra-indications?
45 responses

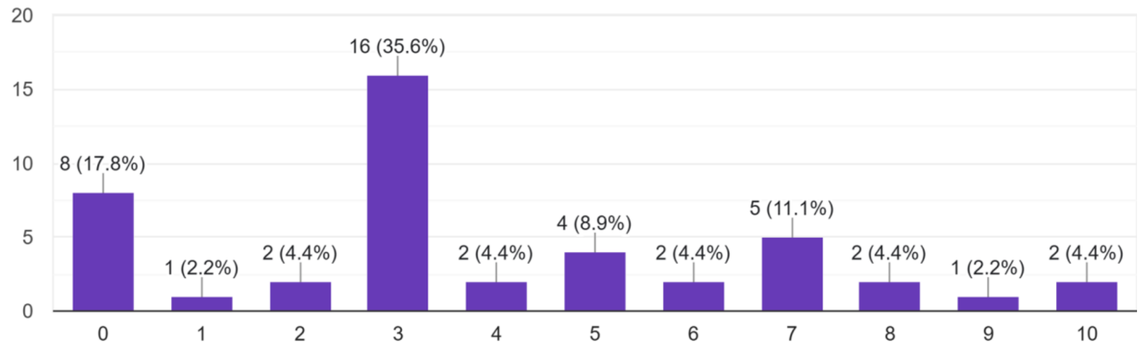

If yes to above, which ones?
31 responses

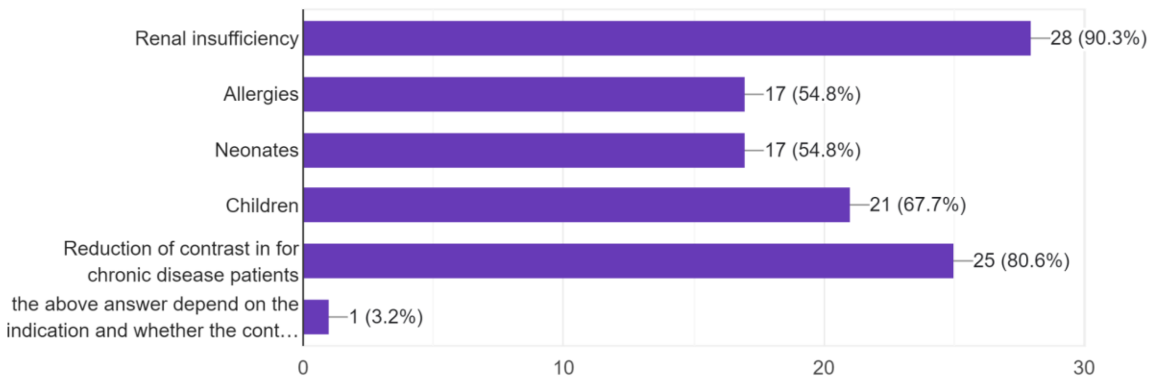

Specific comments

24. Neuro indications

- I think IVIM may prove important for early diagnostics of neurodegenerative diseases. It would be very valuable to compare IVIM in the normal brain to IVIM in patients with mild cognitive impairment (precursor stage to more severe neurodegenerative disease e.g. Alzheimer's).
- Unclear, for acute stroke, CT is commonly used
- In my experience with pre-clinical research of flow augmentation stroke treatment, IVIM f and D* show increased perfusion upon augmentation and correlation with corresponding infarct growth. D and ADC agree strongly regarding infarct volume. f and D* also agree with independently measured collateral supply suggesting as IVIM is endogenous there is not the artificial delay that leads to false hypo perfusion observed with DSC. As collateral supply seems to influence flow augmentation treatment results, it could be a useful method in clinical stroke studies.
- I think we need to show the benefits of IVIM over DTI, esp DTI-ALPS. This is the hot biomarker
- Stroke, oncology, epilepsy
- The potential bias introduced by the kurtosis effect is a major concern in neuro

25. Body indications

- Preliminary data for treatment response assessment
- Rather ADC for large clinical trial
- My feeling is IVIM is not very useful
- I also think IVIM is worth for studying systems at population level, instead of at individual level (e.g. understanding that on average a medicine causes kidneys to be more perfused)
- Tumor characterization and treatment response
- Ongoing research in IVIM DWI for breast imaging is advancing on multiple fronts. Investigators are delving into advanced image acquisition methods and employing both data fitting and model-free data analysis techniques for data analysis. Efforts are being directed towards protocol refinement, aiming to enhance the clinical utility of IVIM in diverse aspects of breast cancer management. A notable challenge lies in the standardization of imaging protocols and the optimization of analysis techniques, particularly in the quest to establish threshold values for IVIM parameters. While many studies focus on distinguishing between different groups, the translation of these findings to the management of individual patient cases poses a significant challenge.
- A pattern of increased mean and stdev D in malignant renal masses. f and D* need more preliminary proof and a consistent sequence and fitting algorithm.
- Oncology
- Lots of potential (eg: in liver) but reproducibility/stability needs to be better before conclusive studies can be conducted

26. MSK indications

- Restsing IVIM muscle perfusion should also be considered particularly in microvascular disease

27. Oncology indications

- The challenge is of course always the repeatability / SNR, and whether D* is useful or not
- Unclear
- Treatment stratification. For tumour detection I think IVIM can play a role, but I do not see added value over classical DWI
- Correlations between PDL1 and IVIM parameters; correlation between IVIM parameters and response after loco-regional HCC therapy

28. Fetal / placental imaging

- Not an expert on this - what can be seen better with IVIM than with ultrasound?
- Difficulty in placental IVIM is understanding the physical significance of IVIM parameters. We know they are altered in disease, but the underlying biology which causes these changes are unclear. The placenta has a range of different flows/lengthscales of motion all within one organ which makes interpreting more difficult, but very interesting!

29. IVIM for perfusion

- IVIM measures probably something different, but it might be closely enough related. Not sure about paediatric applications due to the noise of the DW gradients / EPI readout
- I think IVIM has potential to assess capillary flow characteristics which otherwise require the use of contrast agent. If this could be achieved then perfusion metrics could be obtained without the need for contrast agents in the majority of cases.
- I do not think IVIM f can be a perfect replacement for contrast-enhanced perfusion methods, or ASL. They are complementary, but not interchangeable.
- IVIM perfusion enhances cancer detection and treatment assessment. Starting with a low initial b-value minimizes bias in ADC, improving cancer differentiation. IVIM can be a useful alternative when patients can't use gadolinium contrast, serving as an auxiliary technique when contrast-enhanced MRI is not possible.

30. Other comments

- My experience is limited to the use of IVIM in the kidney
- Only with M2-compensated gradients, otherwise there is too much motion
- My responses reflect modest experience from studies I have been involved in directly, and not my assessment from the literature. Note that my research focus has been on methods development, rather than specific clinical application.
- We still do not know what IVIM measures
- Issues of practicality - short scan time, automated post processing with the key outputs needs to be optimized. Otherwise, there will continue to be limited clinical translation
