# Supplementary material for: Towards Clinical Translation of Intravoxel Incoherent Motion MRI: Acquisition and Analysis Consensus Recommendations
Source: J Magn Reson Imaging. 2026 Mar 19;63(6):1782–801. doi: 10.1002/jmri.70278 (PMC13175230; doi:10.1002/jmri.70278)

Supplemental Information 2: Survey Part 2

Belonging to:
“Towards Clinical Translation of Intravoxel Incoherent Motion MRI: Acquisition and Analysis Consensus Recommendations”, *JMRI*, Sigmund et al.

Number of respondents per organ

Organ	Kidney	Breast	Liver	Muscle	Brain	Pancreas
**Respondents**	12	8	5	4	5	2

Supplemental Material Survey Part 2, Table S1

Analysis and reporting preferences per organ

	kidney	muscle	breast	liver	pancreas	brain
Segmented least squares	100	100	100	100	100	75
one step / simultaneous least squares	10	25	0	25	0	0
fixed D*	10	0	60	0	0	0
Bayesian	60		100	100	100	100
Deep learning		100	100	100	100	100
Noise correction	57.1	33.3	80	0	100	50
post-hoc epi distortion correction	100	66.7	66.7	0	100	100
Post-hoc motion correction	100	33.3	42.9	100	100	100
roi on ADC	36.4	0	75	0	50	
roi on b0	90.9	100	37.5	100	50	100
manual roi	91.7	100	100	100	50	100
automatic roi	42.9	0	50	0	100	100
2D roi	75	50	87.5	80	50	0
3D roi	80	100	87.5	100	100	100
mean value reporting	91.7	100	100	80	100	66.7
max/min reporting	80	100	87.5	60	50	0
histogram reporting	100	100	85.7	60	50	100

Supplemental Material Survey Part 2, Table S2: Percentage (%) agreement among survey part 2 respondents for specific analysis and reporting preferences per organ.

Supplemental Information to "Towards Clinical Translation of Intravoxel Incoherent Motion MRI: Acquisition and Analysis Consensus Recommendations" by Sigmund et al.

Kidney consensus preferences (n = 12)

Acquisition settings:	Survey averages	Confidence level (%)
Field strength	3t	63%
Sequence (single shot/multi-shot EPI)	single shot EPI	80%
FOV	380 +/- 40 mm	
Resolution/interpolation	2.48 mm / no interpolation	62.5%
Slice thickness/gap	4.6 mm +/- 0.9 / with gap	63.6% for gap
TR	3100 +/- 1312 ms	
TE	minimum allowed	100%
Parallel imaging factor	2	100%
Bandwidth (readout)	1978 +/- 250 Hz/px	
Bandwidth (phase encoding)		
Fat suppression	SPAIR	100%
Respiratory motion compensation	respiratory gated	82%
DWI settings:		
b-values		
Encoding gradient shapes	monopolar	80%
Diffusion directions	3	66%
Signal averages	1	44%

Note: Confidence level refers to the fraction of respondents that had suggested values consistent with the survey averages.

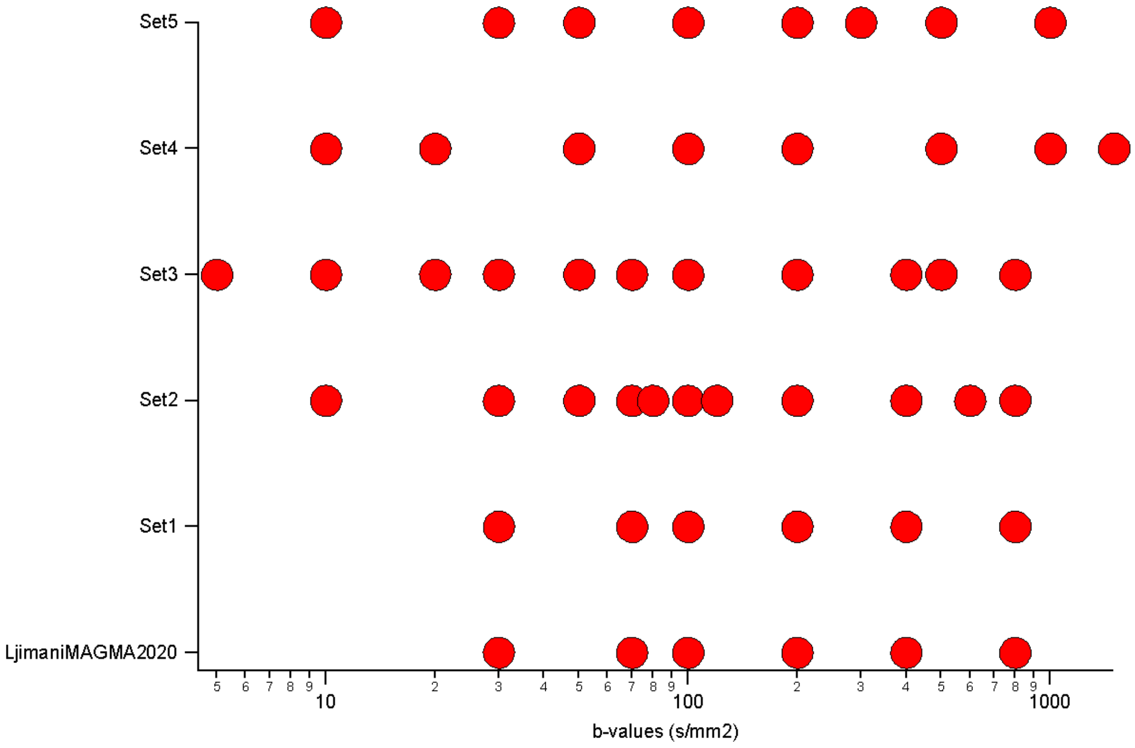

Supplemental Information to “Towards Clinical Translation of Intravoxel Incoherent Motion MRI: Acquisition and Analysis Consensus Recommendations” by Sigmund et al.

Kidney survey comments

Fat suppression
- None
- SPAIR usually works sufficiently well. Maybe for obese volunteers or if high b-values are used, STIR can be an option
- SPAIR can increase acquisition time a lot, especially for multiple b-val acquisitions like for IVIM

TE
- Lower would be better
- We have investigated the TE depedence and have found little impact on IVIM parameters. Minimum TE is beneficial for SNR.

Breathing mode
- I don't think breath-hold acquisition is feasible for DWI (too long)
- Post-hoc registration needed
- Respiratory gating may not be really necessary for allografts

Sequence comments
- Preferred: single shot EPI (SE-EPI)
- Monopolar requires sufficiently good eddy current compensation. Works fine with our Siemens scanners. Not sure if this also works for all other vendors (no experience here). Twice refocused generally comes with a reduced image quality is my experience.

Image processing comments
- Possible removal of single slices from the fitting
- Raw data needs to be of enough quality
- Care must be taken not to change the signal values. We had this problem once in a muscle IVIM study.

B-values comments
- We have been using 13, but the number could be reduced.
- Depends on the fitting model
- I usually take some from previous optimization papers.
- 9 b-values for IVIM; may have to expand to higher b-values for other models

Literature references
- Ljimani et al. MAGMA. 2020; 33(1): 177–195. doi:10.1007/s10334-019-00790-y
- DOI10.1002/nbm.3623, I think IVIM combined with DKI should be encouraged: doi: 10.1002/jmri.24985
- E.g. https://pubmed.ncbi.nlm.nih.gov/21549538/
- An optimized b-value distribution for triexponential intravoxel incoherent motion (IVIM) in the liver

Reporting comments

- Median value reporting preferred; automatic ROI placement only in case of proven accuracy of the automatic tool
- Image resolution should be high enough that subregions can be identified so ROIs contain mostly one tissue type
- Prefer automatic analysis based on whole kidney, i.e. layers or segments.
- Median values rather than mean values
- ROIs should be checked in each image. Reporting of median is also advisable (because e.g. D* outliers alter the mean quite a bit sometimes)

Supplemental Information to “Towards Clinical Translation of Intravoxel Incoherent Motion MRI: Acquisition and Analysis Consensus Recommendations” by Sigmund et al.

Breast consensus preferences (n = 8)

Acquisition settings:	Survey averages	Confidence level (%)
Field strength	3 T	100%
Sequence (single shot/multi-shot EPI)	Single refocused SE<br>TRSE<br>Ms-epi	75%<br>25%<br>75%
FOV	306 +/- 116 mm	
Resolution/interpolation	1.88 +/- 0.3 mm/ interp	50% interp
Slice thickness/gap	4 +/- 0.76 mm no gap	75% no gap
TR	4425 +/- 2449 ms	
TE	minimum	75%
Parallel imaging factor	2.22 +/-0.42	
Bandwidth (readout)	1695 +/- 325 Hz/px	
Bandwidth (phase encoding)		
Fat suppression	SPAIR / SSGR	75% / 50%
Respiratory motion compensation	Free breathing	87.5%
DWI settings:		
b-values		
Encoding gradient shapes		
Diffusion directions	3	100%
Signal averages	2.3 +/-0.4	87.5%

Note: Confidence level refers to the fraction of respondents that had suggested values consistent with the survey averages.

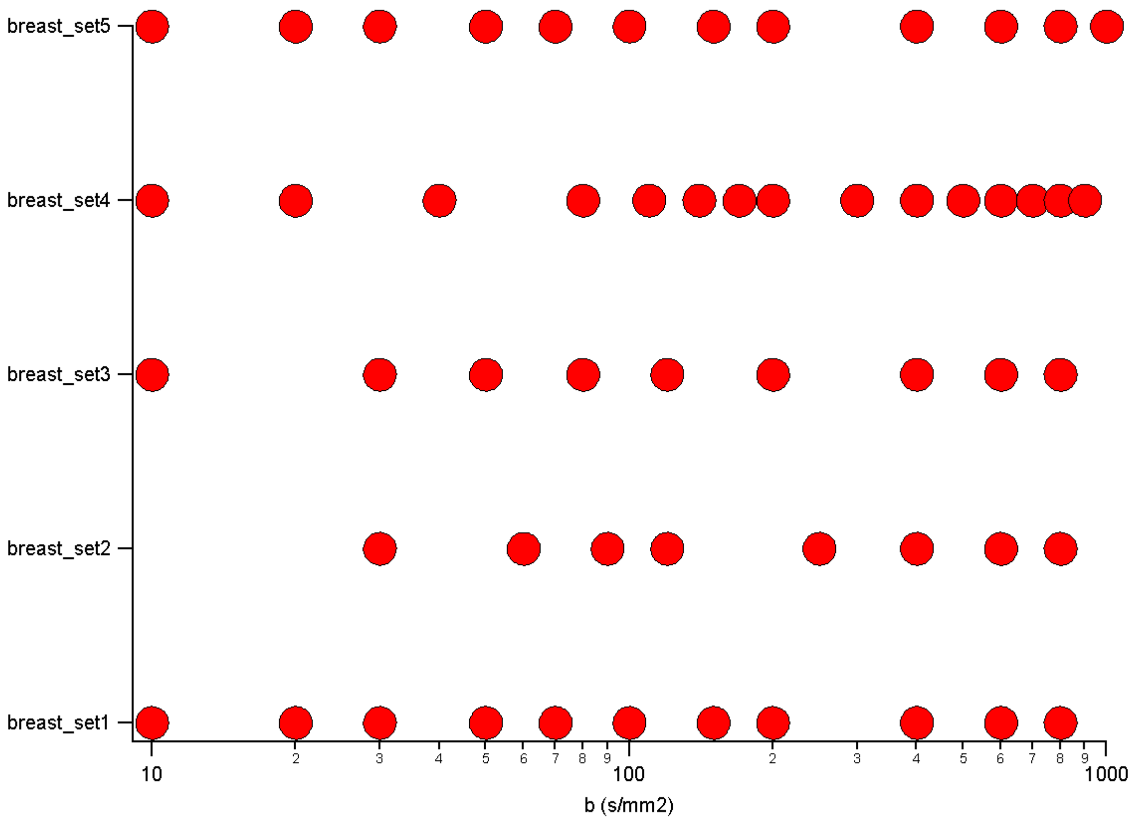

Supplemental Information to “Towards Clinical Translation of Intravoxel Incoherent Motion MRI: Acquisition and Analysis Consensus Recommendations” by Sigmund et al.

Breast survey comments

TR

- TR can be adjusted based on coverage
- >= 3500 ms

TE

- Optimize TE option on GE used (to set min TE)
- ~ 60 ms for max b value = 800

Sequence

- Multi-shot and rFOVtechniques take too long for practical value in combination with IVIM, but this may change in the future with SMS and AI-enhanced recons
- Multi-shot-EPI could be great if it can be used with SMS-acquisitions (to keep scan duration short)

b-value

- For breast we do not include b>1000 in IVIM modeling but useful for kurtosis and other approaches

Algorithm

- We started performing Bayesian approach as neural networks

Image quality

- EPI geometric distortion correction not very successful yet for breast but would like to use it
- EPI correction could be good but not yet successfully implemented at our site

ROI analysis

- Standardly define on b800 DWI images, using manual definition
- b = 800 image might be better for ROI segmentation

Supplemental Information to “Towards Clinical Translation of Intravoxel Incoherent Motion MRI: Acquisition and Analysis Consensus Recommendations” by Sigmund et al.

Liver consensus preferences (n = 5)

Acquisition settings:	Survey averages	Confidence level (%)
Field strength	3T	60%
Sequence (single shot/multi-shot EPI)	Single shot	80%
FOV	400	100%
Resolution/interpolation	2.7+/-1.0 mm	
Slice thickness/gap	5.5 +/- 2 mm / with gap	100% gap
TR	3887 +/- 876 ms	
TE	Minimum allowed	80%
Parallel imaging factor	2	100%
Bandwidth (readout)	1897 +/- 1100 Hz/px	
Bandwidth (phase encoding)		
Fat suppression	SPAIR	80%
Respiratory motion compensation	Free breathing or respiratory triggered	Both 80%
DWI settings:		
b-values		
Encoding gradient shapes	Monopolar single/bipolar TRSE	Both 60%
Diffusion directions	3	80%
Signal averages	1.6 +/- 1.1	

Note: Confidence level refers to the fraction of respondents that had suggested values consistent with the survey averages.

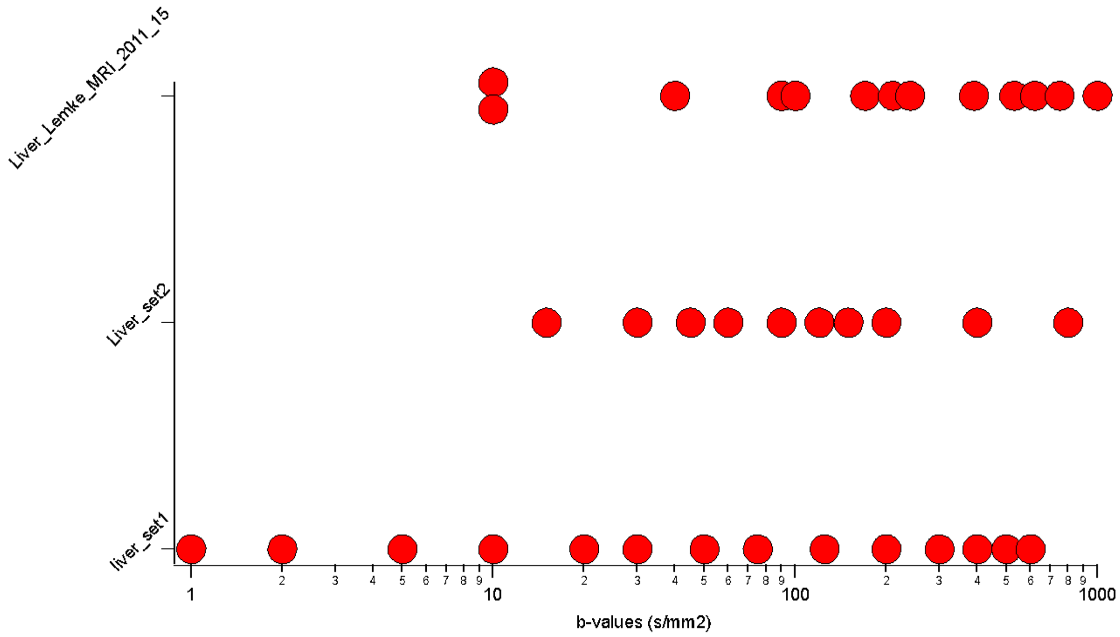

Supplemental Information to “Towards Clinical Translation of Intravoxel Incoherent Motion MRI: Acquisition and Analysis Consensus Recommendations” by Sigmund et al.

Liver survey comments

TE

- Adding a few ms to the minimum TE may give more robust scans
- f values depend heavily on TE, but I am not sure about how to fix it. TE = 90 ms generally came with a much degraded image quality in our studies

Breathing mode

- Breath hold may be useful when smaller lesions are of interest
- Respiratory triggered is the preferred approach to me
- All options are viable, I guess. Free breathing allows for acquiring more data and for fixing TR. For IVIM imaging of liver parenchyma, this may outweigh the disadvantage of image blurring/shifts. For imaging of lesions, the opposite may be true.
- Free breathing is ok with post-hoc motion correction

Pulse sequence

- Bipolar gradient can help with advanced modeling
- rFOV makes little sense, I guess, because the liver is quite large. Pulsation artifact especially in the left liver lobe may be quite relevant.
- SE-EPI

Fit algorithm

- Deep learning > Bayesian > segmented
- One should strive for sufficiently high SNR instead of noise floor correction. Usually, this should be possible.

Image quality

- Nonlinear motion correction usually need
- Depends on purpose. Tumor imaging --> MoCo; MAFLD/whole liver --> no MoConeeded

Roi analysis

- Lesions or diffuse conditions may require different ROI types
- Median value seems most appropriate. Max/Min are outliers and would prefer IQR in that case. ROI placement on high b-value for tumours

Supplemental Information to “Towards Clinical Translation of Intravoxel Incoherent Motion MRI: Acquisition and Analysis Consensus Recommendations” by Sigmund et al.

Muscle consensus preferences (n = 4)

Acquisition settings:	Survey averages	Confidence level (%)
Field strength	3 T	75%
Sequence (single shot/multi-shot EPI)	Single shot spin echo	75%
FOV	375 +/- 35 mm	Depends on area
Resolution/interpolation	2.5+/-0.6 mm / none	
Slice thickness/gap	5.1+/-0.25 / gap	50% for gap
TR	4467 +/- 2540 ms	
TE	Minimum TE	100%
Parallel imaging factor	2	100%
Bandwidth (readout)	2373 +/- 622 Hz/ px	
Bandwidth (phase encoding)	62.5%	25%
Fat suppression	SPAIR / SSGR	100% / 75%
Respiratory motion compensation	Free breathing	75%
DWI settings:		
b-values		
Encoding gradient shapes	monopolar	
Diffusion directions	3	75%
Signal averages	2.5 +/- 0.8	

Note: Confidence level refers to the fraction of respondents that had suggested values consistent with the survey averages.

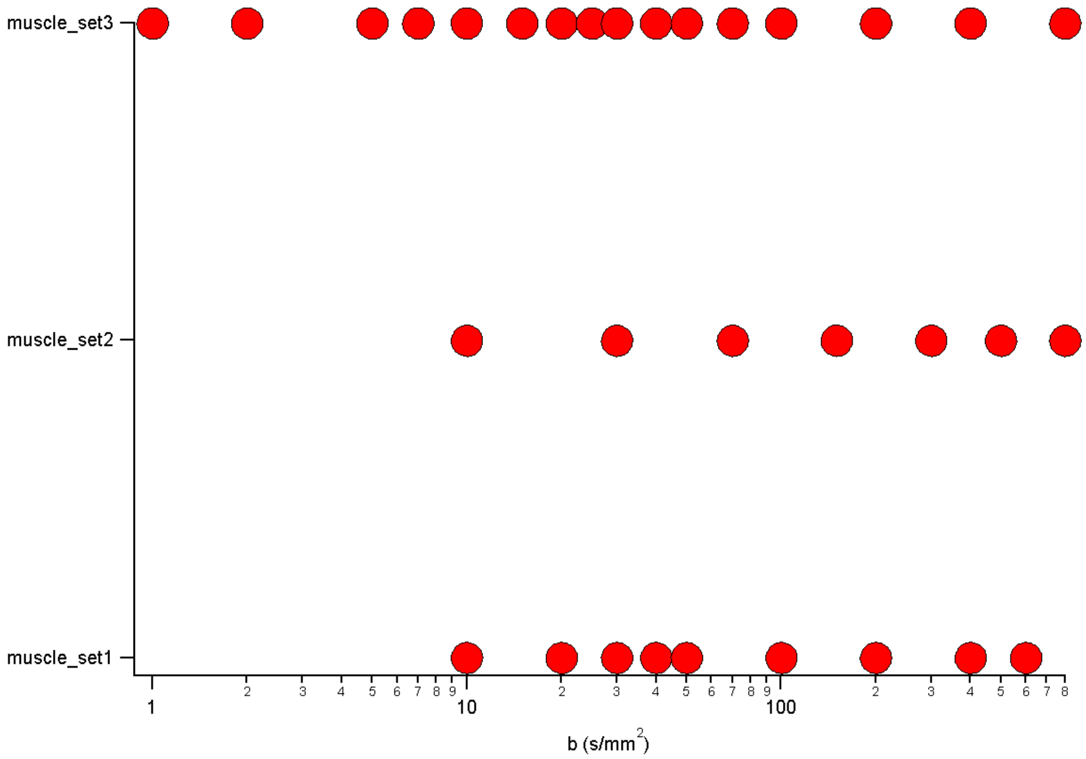

Supplemental Information to “Towards Clinical Translation of Intravoxel Incoherent Motion MRI: Acquisition and Analysis Consensus Recommendations” by Sigmund et al.

Muscle survey comments

Resolution
- Make sure that sufficient SNR is available

FOV
- Depends on the anatomy (i.e. one leg/both legs, lower/upper leg)
- 400
- Depends on body region. I think slice gaps are generally important since inflow effects are potentially not negligible.
- 350 x 220 mm^2

Directions
- More directions at high b-values to allow DTI fit. Smaller b-values (<200): 3-6 directions
- 3
- Maybe 6 if DTI fits are included. The FOV questions cannot be answered in general as they depend on the muscle to be imaged.
- Orthogonal/tetrahedral depending on time limitations

Fat suppression
- Skeletal muscle: olefinic fat peak should be suppressed as well with an additional pulse
- In the calf muscle, I have the experience that a shifted fat ring (=subcutaneous fat) is sometimes visible in the muscle. One should avoid this region in the ROI placement.

TE
- Should not be much longer than 60 ms
- Muscle tissue has a quite short T2 time. My experience is that long TE often result in a quite bad image quality, which makes one have little trust in the IVIM fits.

Breathing mode
- For most skeletal muscle applications not relevant (i.e. legs)

Sequence
- Monopolar requires good eddy current compensation. Multishot and rFOV: depends on muscle to be imaged.

B-values
- Make sure that sufficient signal is available also for diffusion direction || to the muscle direction.

Relevant literature for muscle
- Englund et. al. JMRI 55:988-1012 (2022)
- For muscle IVIM, it is important to report the exact activation pattern (if and which exercise was used, & the timing)

Supplemental Information to “Towards Clinical Translation of Intravoxel Incoherent Motion MRI: Acquisition and Analysis Consensus Recommendations” by Sigmund et al.

Algorithm notes

- I think it is usually possible to achieve a sufficiently high SNR to avoid the necessity for noise floor corrections.
- Fixed D algorithm

Image quality

- Make sure that no shifted fat is in the ROI.

Roi analysis

- Median values are important because of outliers (in particular for D*)

Supplemental Information to “Towards Clinical Translation of Intravoxel Incoherent Motion MRI: Acquisition and Analysis Consensus Recommendations” by Sigmund et al.

Brain consensus preferences (n = 5)

Acquisition settings:	Survey averages	Confidence level (%)
Field strength	3 T	75%
Sequence (single shot/multi-shot EPI)	Single shot single echo<br>TRSE	20%<br>20%
FOV	221 mm	
Resolution/interpolation	1.95 +/-0.67 mm / none	100%
Slice thickness/gap	2.45 +/- 1.23 mm / none	50% no gap
TR	4500+/-707 ms	
TE	minimum	100%
Parallel imaging factor	2.3 +/- 0.6	
Bandwidth (readout)		
Bandwidth (phase encoding)		
Fat suppression	SPIR	60%
Respiratory motion compensation	Free breathing	
DWI settings:		
b-values		
Encoding gradient shapes	Monopolar / bipolar	20% / 20%
Diffusion directions	6	50%
Signal averages	1	100%

Note: Confidence level refers to the fraction of respondents that had suggested values consistent with the survey averages.

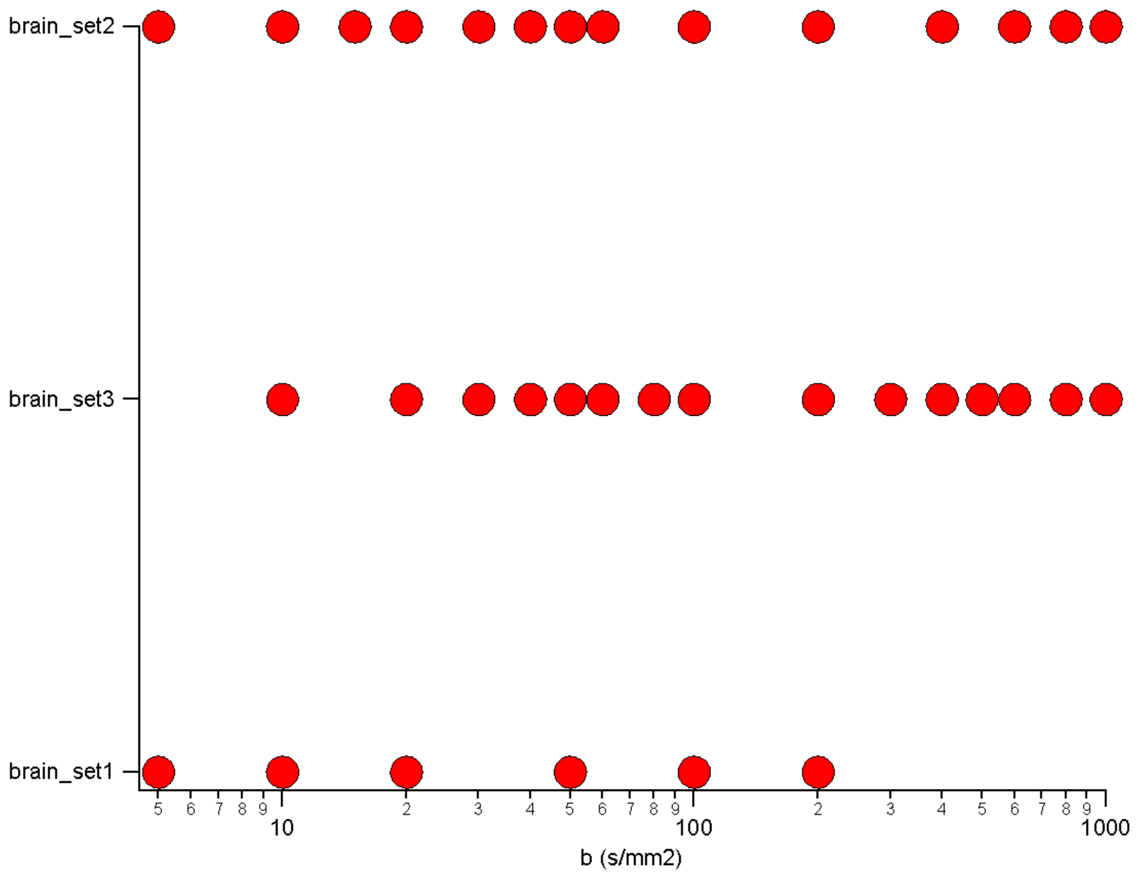

Supplemental Information to “Towards Clinical Translation of Intravoxel Incoherent Motion MRI: Acquisition and Analysis Consensus Recommendations” by Sigmund et al.

Pancreas consensus preferences (n = 2)

Acquisition settings:	Survey averages	Confidence level (%)
Field strength	3 T / 1.5 T	50% / 50%
Sequence (single shot/multi-shot EPI)	Single shot EPI	
FOV		
Resolution/interpolation	2 mm	Interp 50%
Slice thickness/gap	3 Mm / no gap	100%
TR	3000	
TE	minimum	
Parallel imaging factor	2	
Bandwidth (readout)	1568 Hz/px	
Bandwidth (phase encoding)		
Fat suppression	SPAIR / SSGR	
Respiratory motion compensation	Respiratory gated	50%
DWI settings:		
b-values		
Encoding gradient shapes	monopolar	
Diffusion directions	Average 14	
Signal averages	3.5 (more for low b)	

Note: Confidence level refers to the fraction of respondents that had suggested values consistent with the survey averages.

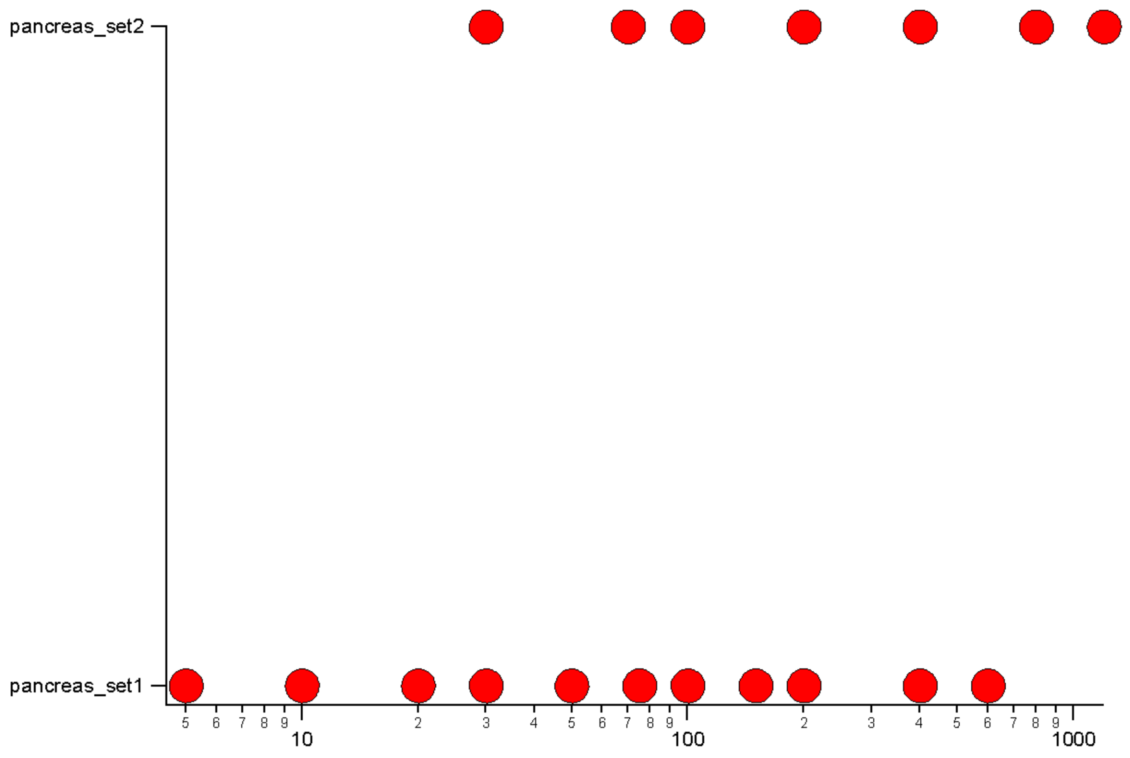

Supplement: Supplementary file 2 — Supplementary Information 2 Survey Part 2. [file JMRI-63-1782-s001.pdf]
