# Supplementary material for: Towards Clinical Translation of Intravoxel Incoherent Motion MRI: Acquisition and Analysis Consensus Recommendations
Source: J Magn Reson Imaging. 2026 Mar 19;63(6):1782–801. doi: 10.1002/jmri.70278 (PMC13175230; doi:10.1002/jmri.70278)

Supplemental Information 3: Workshop Polls

Belonging to:
"Towards Clinical Translation of Intravoxel Incoherent Motion MRI: Acquisition and Analysis Consensus Recommendations", *JMRI*, Sigmund et al.

Results from the 21 additional polls taken during the IVIM workshop.

1. What are the most prominent IVIM applications? (n = 53 votes)

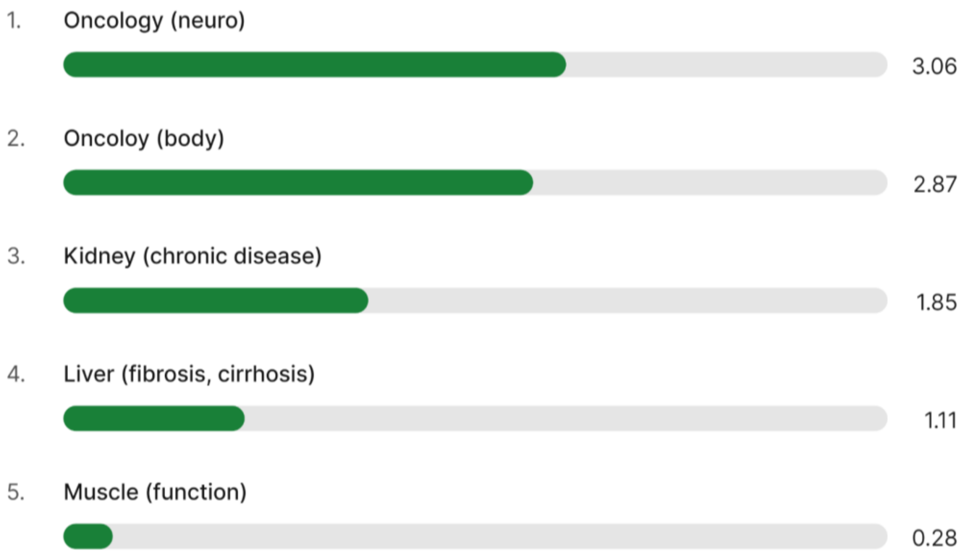

<u>2. What is your training?</u> (n = 61 votes)

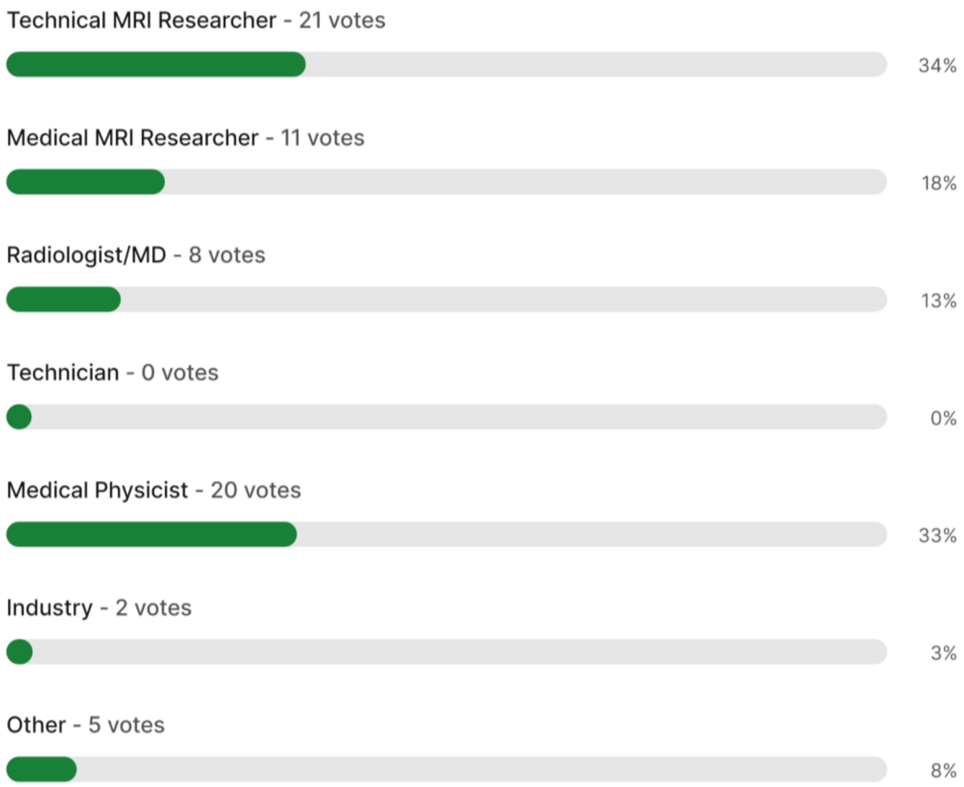

<u>3. What body part(s) do you work on?</u> (n = 96 responses)

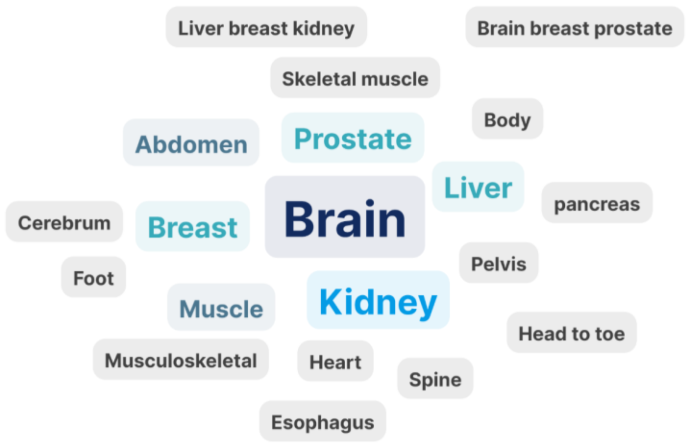

4. Where are you from? (n = 67 responses)

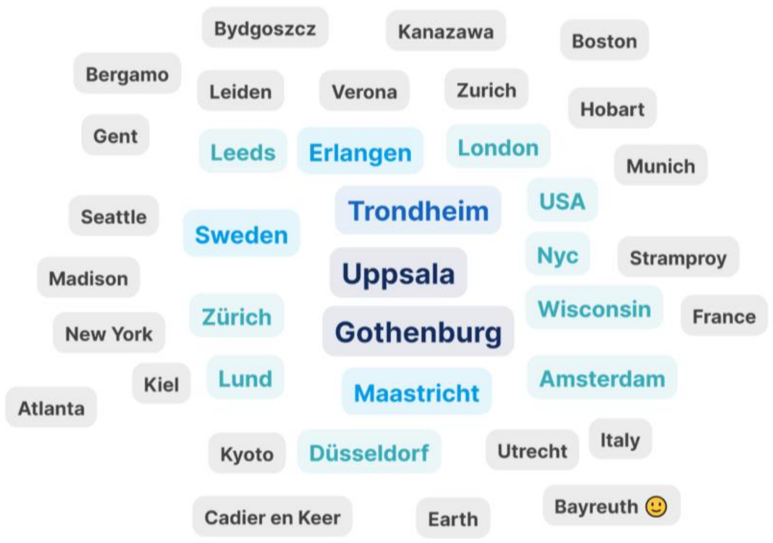

5. What fitting method is preferred in general? (n = 39 votes)

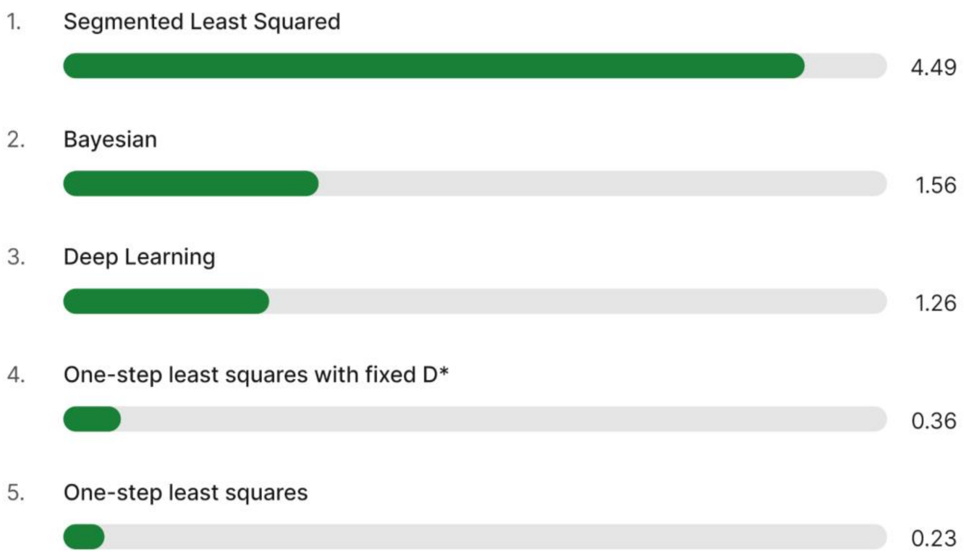

6. What fitting method do we want in a standardized pipeline? (n = 37 votes)

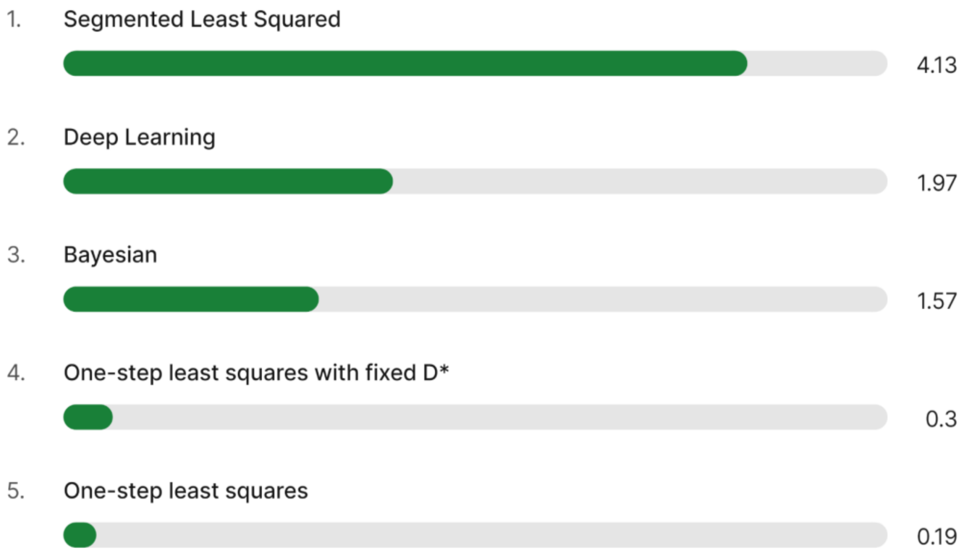

7. What model to use? (n = 46 votes)

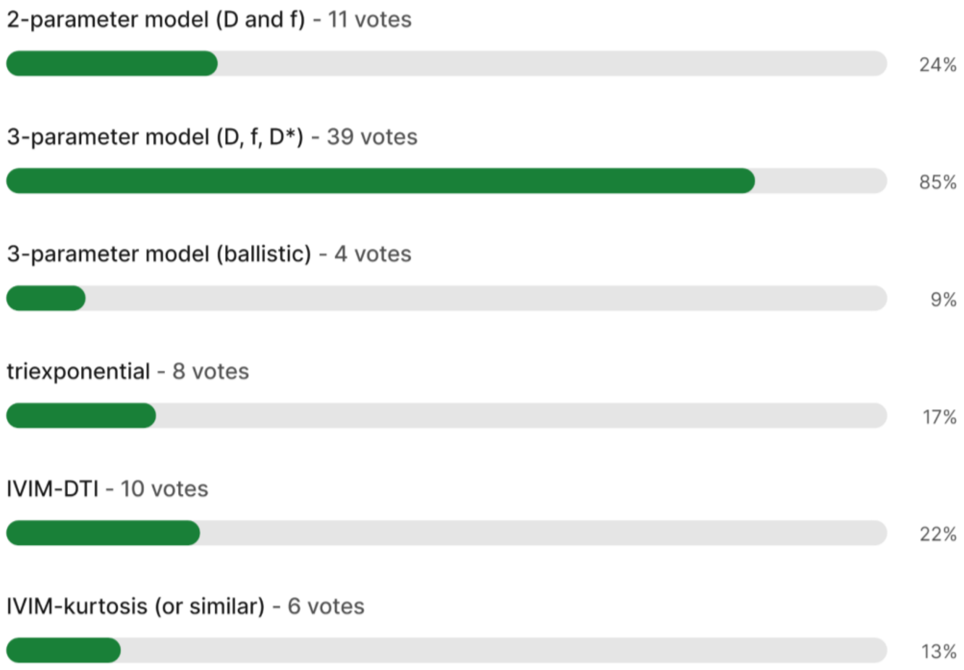

8. In a standardized Pipeline, what pre-processing should be done (brain)? (n = 30 responses)

- Motion correction (n = 8)
- Distortion correction (n = 7)
- Denoising (n = 6)
- Motion correction/ geometric correction
- Distortion, motion, denoising
- Distortion correction and motion correction
- Susceptibility correction
- EPI, eddy current and motion correction
- Registration
- Acquire image
- susceptibility correction
- Denoise, register, fit
- Geometric distortion
- EPI and motion correction (brain)

9. In a standardized Pipeline, what pre-processing should be done (abdomen)? (n = 21 responses)

- Motion correction (n = 14)
- Denoising (n = 5)
- Distortion correction (n = 4)
- Denoising with abdomen specific techniques
- Removal of signal voids due to compressive motion, eg in liver and pancreas

10. In a standardized Pipeline, what pre-processing should be done (muscle)? (n = 6 responses)

- Check Muscle twitch artifacts
- Remove large signal dropout
- Artifact rejection
- Remove muscle twitch signal drop
- Denoising, motion and distortion correction
- Motion correction

<u>11. What is the main obstacle to clinical translation?</u> (n = 38 votes)

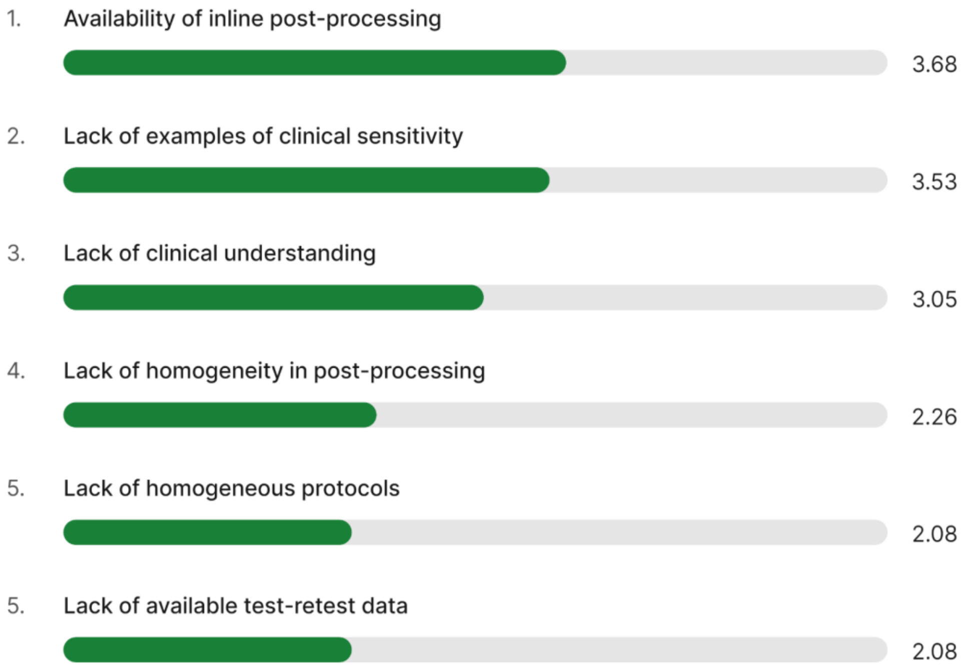

<u>12. What is the next obstacle we should tackle?</u> (n = 36 responses)

- Nomenclature & precise language (n =4)
- Integration in radiology workflow (PACS) or inline processing (n = 3)
- Consensus and standardization of fitting algorithm (n = 3)
- Standardization protocol and analysis (n = 2)
- IVIM standardization (n = 2)
- Validation of protocols & fitting algorithms (n = 2)
- Getting f maps into the clinical routine (n = 2)
- Sharing codes (n = 2)
- Quantitative b-value standardisation (range and distribution)
- Standardized phantoms for evaluation
- Histologic validation
- More test retest data
- Identify the first clinical application for this community to advance into clinical trials
- Convince clinicians that IVIM may replace GDCA
- Inclusion in more trials
- Finding a killer application.
- Education for (non-research) radiologists
- Post processing
- Make sure no one is using completely wrong b-values for the model they use
- Minimal versions
- Correcting for confounders
- Distortion correction
- Motion correction
- Do we even have diffusive blood flow at these diffusion times?

<u>13. In a standardized pipeline, what kind of ROI statistics should be reported?</u> (n = 42 votes)

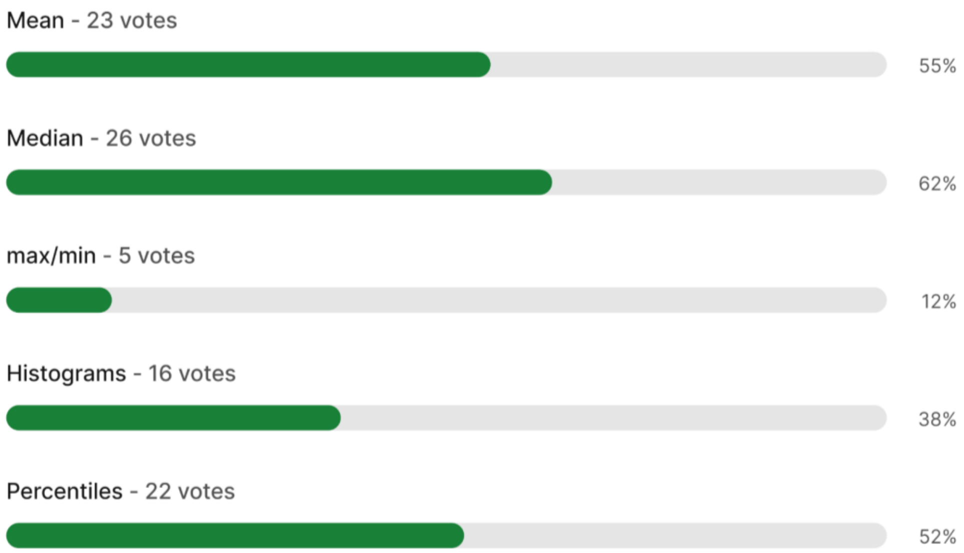

<u>14. Which acquisition discussion will you attend?</u> (n = 39 votes)

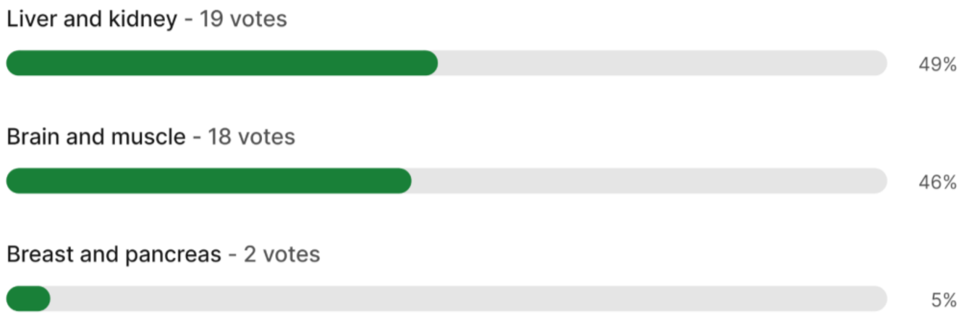

Supplemental Information to “Towards Clinical Translation of Intravoxel Incoherent Motion MRI: Acquisition and Analysis Consensus Recommendations” by Sigmund et al.

Note: The polls were mainly used in the plenary session. Discussions on acquisition and processing steps for the six organ systems were held in smaller subgroups. In those subgroups, polls were only used in the pancreas group, which is why only pancreas poll results are presented here.

15. Pancreas: Motion management (n = 7 votes)

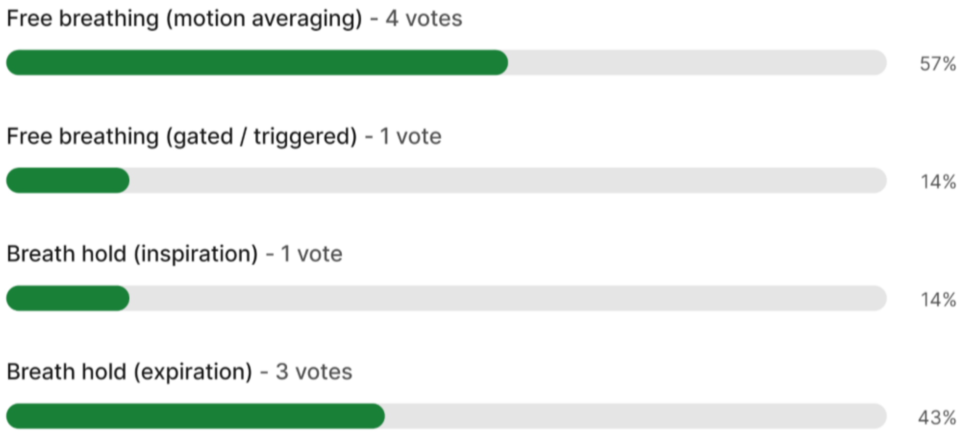

16. Pancreas: Fat suppression (n = 7 votes)

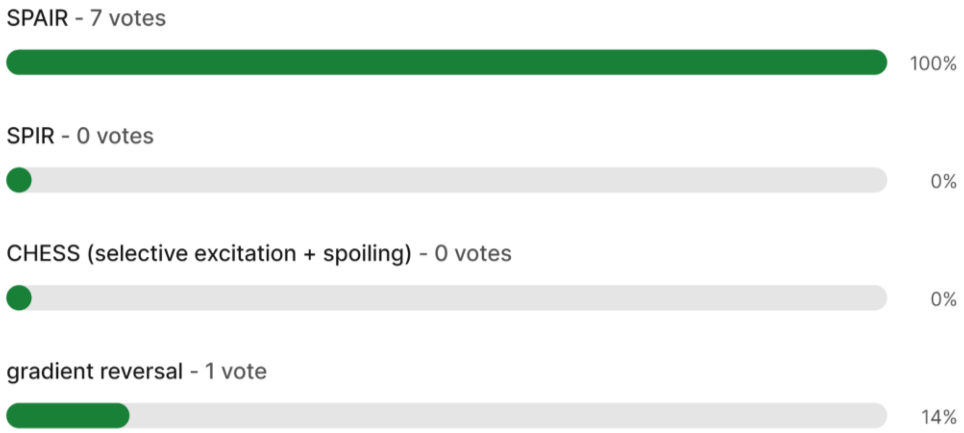

17. Pancreas: Patient preparation (n = 7 votes)

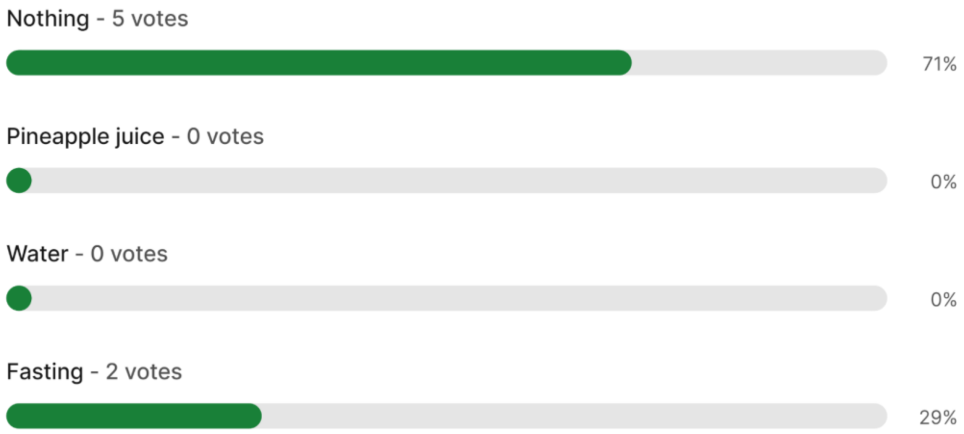

<u>18. Pancreas: Slice acquisition order</u> (n = 7 votes)

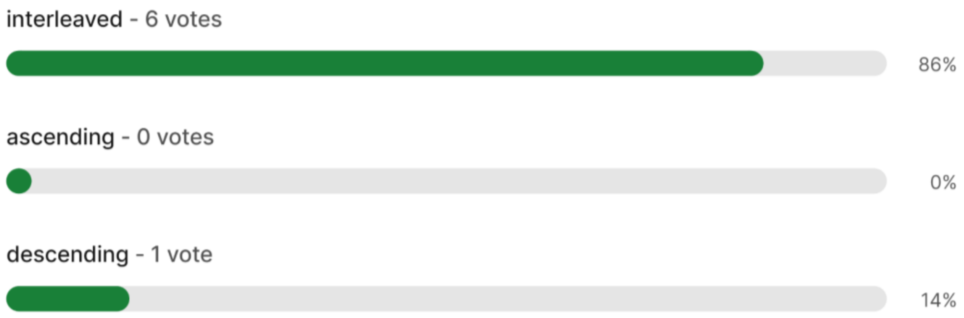

<u>19. Pancreas: Number of diffusion-weighting directions</u> (n = 8 votes)

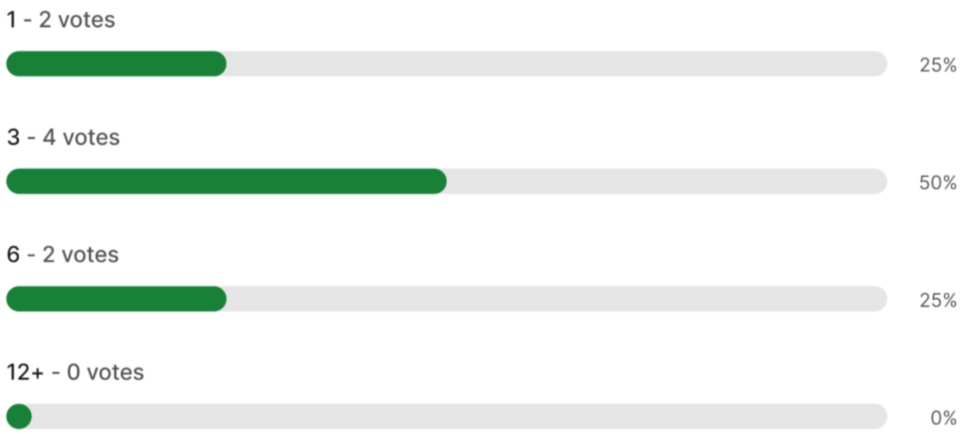

<u>20. Pancreas: Which parameters should be analyzed</u> (n = 8 votes)

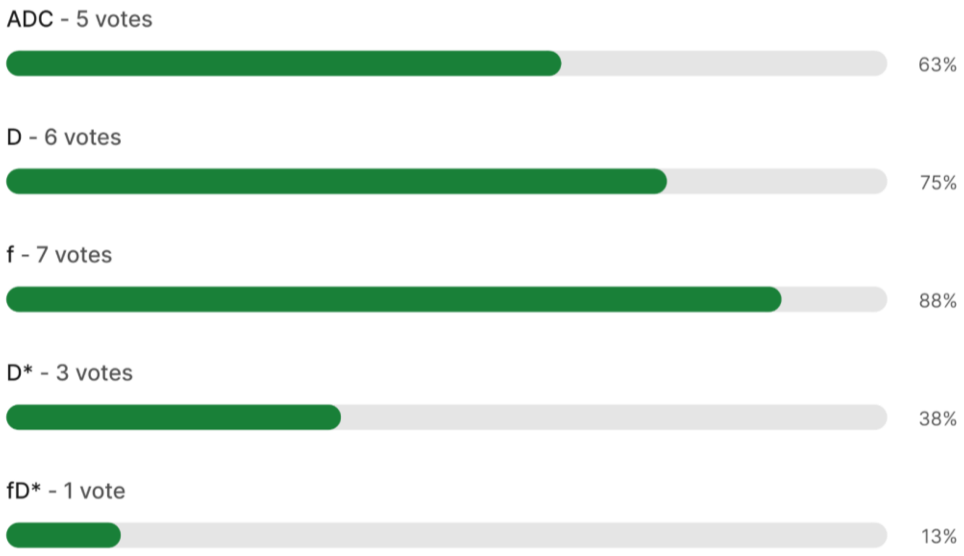

21. Pancreas: How to fit the IVIM model? (n = 8 votes)

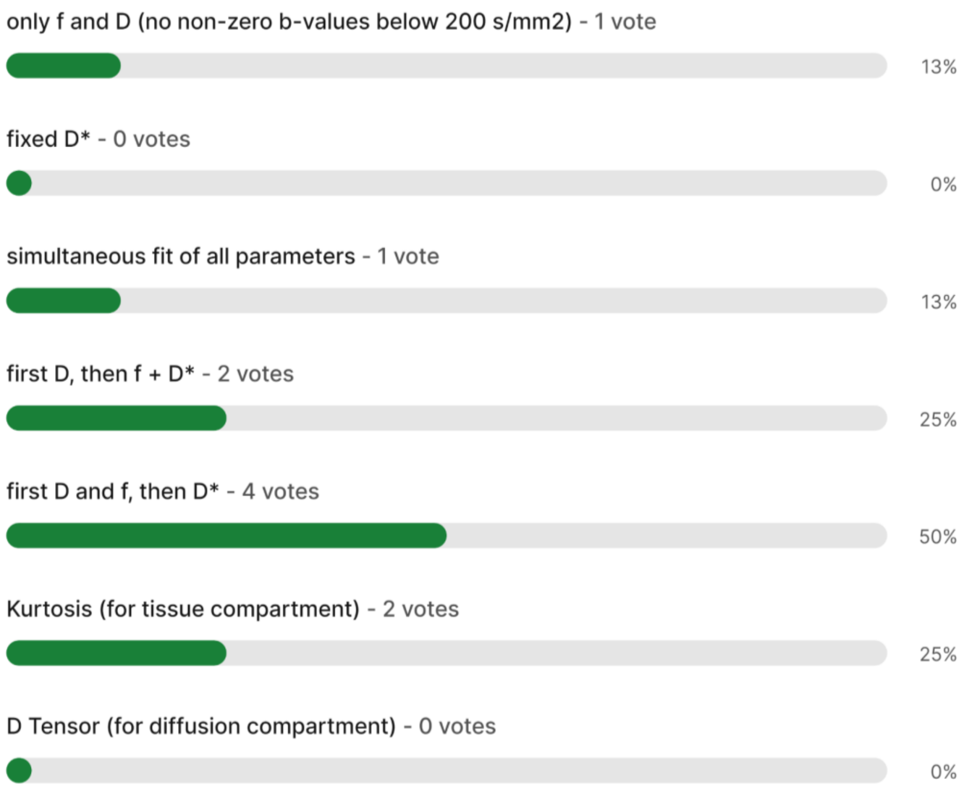

Supplement: Supplementary file 3 — Supplementary Information 3 Workshop Polls. [file JMRI-63-1782-s004.pdf]
