# Supplementary material for: Towards Clinical Translation of Intravoxel Incoherent Motion MRI: Acquisition and Analysis Consensus Recommendations
Source: J Magn Reson Imaging. 2026 Mar 19;63(6):1782–801. doi: 10.1002/jmri.70278 (PMC13175230; doi:10.1002/jmri.70278)
Supplement: Supplementary file 4 — Supplementary Information 4 Recommendations. [file JMRI-63-1782-s005.pdf]

Supplemental Information 4: Recommendations

Belonging to:
“Towards Clinical Translation of Intravoxel Incoherent Motion MRI: Acquisition and Analysis Consensus Recommendations”, *JMRI*, Sigmund et al.

Supplemental Information 4: Recommendations Table S1 Mean IVIM values ± standard deviations (coefficient of variation) over studies derived from meta-analyses and review articles for 6 organ systems. Diffusivities are given in units of 10^{-3} mm^2/s, and *f* is given in %.

**Organ**	**Review**	**Studies**	***D*** **(10^{-3} mm^2/s)**	***f*** **(%)**	***D**** **(10^{-3} mm^2/s)**
Brain	Vieni 2020 [1]	11	0.83 ± 0.07 (8.9%)	7.64 ± 3.16 (41.3%)	10.88 ± 4.78 (44.0%)
Kidney	Ljimani 2020 [2]	18	1.89 ± 0.15 (8.0%)	18.88 ± 6.23 (33.0%)	40.53 ± 19.63 (48.4%)
Liver	Li 2017 [3]	27	1.09 ± 0.17 (15.6%)	23.05 ± 8.48 (36.8%)	70.02 ± 31.01 (44.3%)
Muscle	Englund 2022 [4]	26	1.47 ± 0.28 (19.3%)	10.34 ± 5.92 (57.2%)	30.88 ± 39.18 (126.9%)
Breast (benign)	Liang 2020 [5]	12	1.43 ± 0.37 (25.8%)	7.00 ± 4.22 (60.3%)	52.33 ± 28.50 (54.5 %)
Breast (malignant)			0.97 ± 0.32 (32.9%)	11.31 ± 4.51 (39.8%)	37.76 ± 19.12 (50.6 %)
Pancreas (benign)	Zhu 2021 [6]	8	1.41 ± 0.72 (51.3%)	20.03 ± 7.99 (39.9%)	25.39 ± 13.69 (53.9%)
Pancreas (malignant)			1.40 ± 0.48 (34.3%)	12.39 ± 4.97 (40.1%)	22.16 ± 12.83 (57.9%)

References:

[1] Vieni, C., et al., Effect of intravoxel incoherent motion on diffusion parameters in normal brain. Neuroimage, 2020. 204: p. 116228.
[2] Ljimani, A., et al., Consensus-based technical recommendations for clinical translation of renal diffusion-weighted MRI. MAGMA, 2020. 33(1): p. 177-195.

[3] Li, Y.T., et al., Liver intravoxel incoherent motion (IVIM) magnetic resonance imaging: a comprehensive review of published data on normal values and applications for fibrosis and tumor evaluation. Quant Imaging Med Surg, 2017. 7(1): p. 59-78.
[4] Englund, E.K., et al., Intravoxel Incoherent Motion Magnetic Resonance Imaging in Skeletal Muscle: Review and Future Directions. J Magn Reson Imaging, 2022. 55(4): p. 988-1012.
[5] Liang, J., et al., Intravoxel Incoherent Motion Diffusion-Weighted Imaging for Quantitative Differentiation of Breast Tumors: A Meta-Analysis. Front Oncol, 2020. 10: p. 585486.
[6] Zhu, M., et al., Accuracy of quantitative diffusion-weighted imaging for differentiating benign and malignant pancreatic lesions: a systematic review and meta-analysis. European Radiology, 2021. 31(10): p. 7746-7759.

Supplemental Information 4: Recommendations Text S1

Monte Carlo Simulation Methods

IVIM signal decay data were simulated with equation 1 using average IVIM parameters from the literature (*Supplemental Material Recommendations* Table S1) and both “minimal” and “abbreviated” b-value sets (main paper Table 3). Simulations used complex data where the real channel included simulated IVIM decays combined with Gaussian additive noise and the imaginary channel included only Gaussian noise. Real and imaginary signals were combined as a root sum of squares and fit using nonlinear least squares. Signal to noise ratio (SNR) was defined as the ratio of the b = 0 s/mm² signal amplitude divided by the standard deviation of the noise.

For fitting of abbreviated b-value data, a monoexponential model was fit to decay data above the IVIM threshold value (see main paper Table 3 and accompanying text) for each respective organ context to obtain *D*. The perfusion fraction, *f*, was estimated by subtracting the y-intercept obtained from the monoexponential fit from the b = 0 s/mm² signal intensity.

For the minimal b-value data, a segmented fit was performed where a monoexponential model was first fit to the decay data above the IVIM threshold value to obtain *D* and *f* as described in the abbreviated b-value fitting scheme. Next, the biexponential model was fit to all b-value data constraining the parameters *f* and *D* that were obtained in the first step while estimating *D**.

All fits were performed using nonlinear least squares with the following bounds on parameter estimates (see equations below): α=[0.1, 3]; D=[0.1e-3, 5e-3] mm^2/s; and D*=[0.5e-2, 2] mm^2/s.

Bias and dispersion errors for each IVIM parameter were estimated for each SNR and organ context based on 1,000 independent noise realizations. Bias error is defined as the relative error 100*(estimate – true)/true and dispersion error is defined as the 100*coefficient of variation (CV = standard deviation/mean) of each respective parameter estimate over all noise realizations. Fifteen log-spaced SNR levels between 10 and 1000 were simulated. Since we illustrate SNR dependencies via the minimum SNR needed to ensure the error does not exceed thresholds of 10% or 20%, we employed a high upper limit so as to capture the full range of relevant scenarios to inform study design, so that the collected IVIM biomarkers have the highest potential utility.

All MATLAB (R2021b) scripts are freely available for replication of these results and for performing additional simulations (https://github.com/dareiterlab/MC-simulation-IVIM-errors).

Note that these simulated estimations of SNR requirements do not include effects of signal averaging or ROI averaging (which increases SNR by approximately a factor of the square root of the number of averages or voxels, respectively, though these benefit is altered at low SNR due to Rician statistics of magnitude-averaged signals), or denoising, all of which investigators are encouraged to consider if appropriate.

$$\frac{S(b)}{S_0} = f \cdot e^{-bD*} + (1-f) \cdot e^{-bD} \qquad \textbf{Equation 1}$$

**Step 1: fit using b-values above the IVIM threshold.**	**Step 2: fit using all b-values**
$\frac{S}{S_0} = \alpha \cdot e^{-bD}$	$\frac{S}{S_0} = f \cdot e^{-bD*} + (1-f) \cdot e^{-bD}$
Fit α and D. Compute: $f = 1 - \alpha$	Fit $D*$ while holding f and D fixed

Supplemental Information to “Towards Clinical Translation of Intravoxel Incoherent Motion MRI: Acquisition and Analysis Consensus Recommendations” by Sigmund et al.

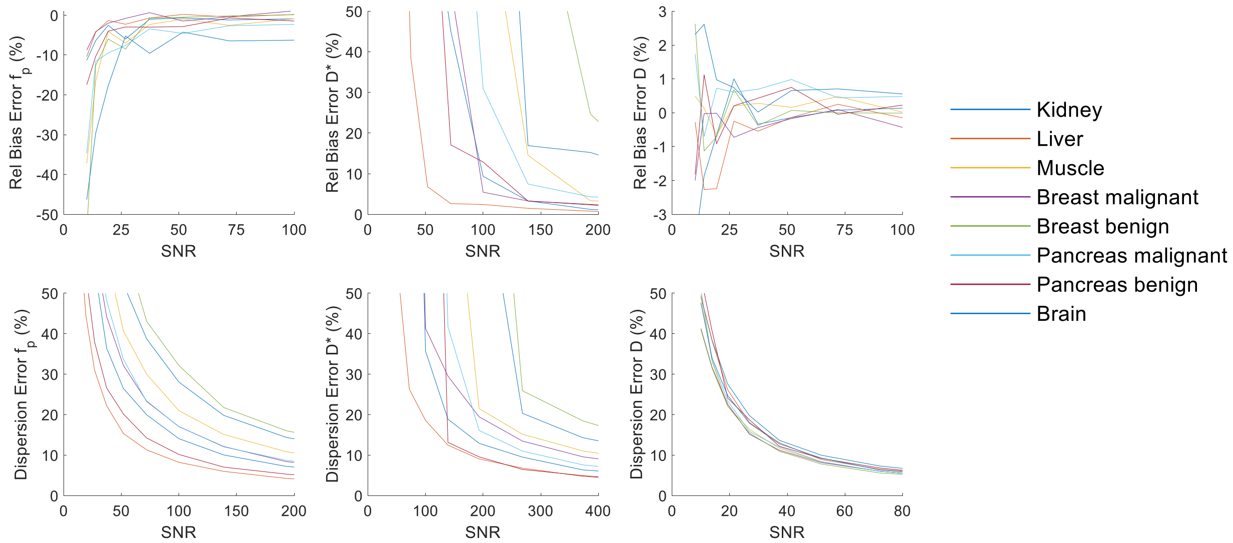

Supplemental Information 4: Recommendations Figure S1. Results from Monte Carlo simulations based on “minimal” recommended b-values showing relative bias (i.e., 100*(estimated – true)/true) and dispersion (i.e., 100*$CV_{parameter}$) errors.

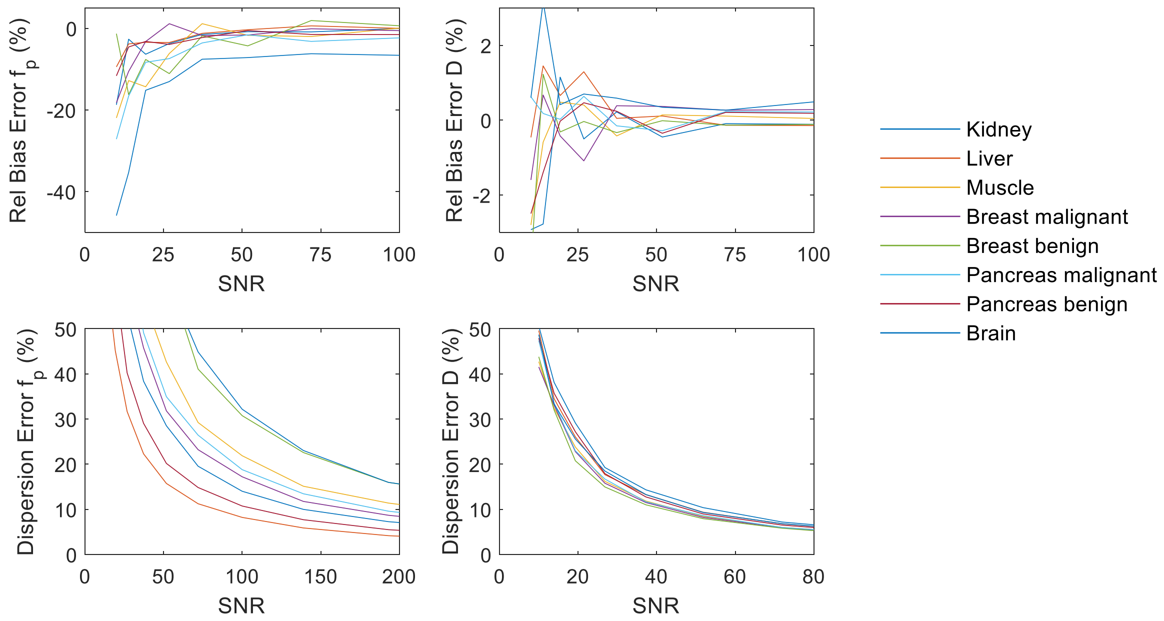

Supplemental Information 4: Recommendations Figure S2. Results from Monte Carlo simulations based on “abbreviated” recommended b-values showing relative bias (i.e., 100*(estimated – true)/true) and dispersion (i.e., 100*$CV_{parameter}$) errors.

Supplemental Information 4: Recommendations Table S2. Minimum SNR necessary for achieving IVIM parameter estimates with bias, dispersion, or total error (defined as the quadrature sum of bias and dispersion errors: Total = $(\text{Bias}^2 + \text{Disp.}^2)^{0.5}$ within 10% and 20% of true value. Results from Monte Carlo simulations based on "minimal" or "abbreviated" recommended b-values (main paper Table 3). Fifteen log-spaced SNR values were simulated between 10 and 1000 (i.e., 10, 13.9, 19.3, 26.8, 37.3, 51.8, 71.9, 100, 138.9, 193.1, 268.3, 372.8, 517.9, 719.7, 1000). Simulations based on 1,000 independent noise realizations per tissue context, per level of SNR.

Set	Metric	Error (%)	Param.	Kidney	Liver	Muscle	Breast malig nant	Breast benign	Pancreas malignant	Pancreas benign	Brain
			**f**	10	10	10	10	10	10	10	10
**Minimal**	**Bias**	**10**	**D***	100	51.8	138.9	100	268.3	138.9	138.9	268.3
			**D**	10	10	10	10	10	10	10	10
			**f**	10	10	10	10	10	10	10	10
**Minimal**	**Bias**	**20**	**D***	100	51.8	138.9	100	268.3	138.9	71.9	138.9
			**D**	10	10	10	10	10	10	10	10
			**f**	193.1	100	268.3	193.1	372.8	193.1	138.9	372.8
**Minimal**	**Disp.**	**10**	**D***	268.3	193.1	517.9	372.8	719.7	372.8	193.1	719.7
			**D**	51.8	51.8	51.8	51.8	51.8	51.8	51.8	51.8
			**f**	71.9	51.7	138.9	100	193.1	100	71.9	138.9
**Minimal**	**Disp.**	**20**	**D***	138.9	100	268.3	193.1	372.8	193.1	138.9	372.8
			**D**	26.8	26.8	26.8	26.8	26.8	26.8	26.8	26.8
			**f**	193.1	100	268.3	193.1	372.8	193.1	138.9	372.8
**Minimal**	**Total**	**10**	**D***	268.3	193.1	517.9	372.8	719.7	372.8	193.1	1000
			**D**	51.8	51.8	51.8	51.8	51.8	51.8	51.8	51.8
			**f**	100	51.7	138.9	100	193.1	100	71.9	193.1
**Minimal**	**Total**	**20**	**D***	138.9	100	268.3	193.1	372.8	193.1	138.9	372.8
			**D**	26.8	26.8	26.8	26.8	26.8	26.8	26.8	26.8
			**f**	13.9	10	26.8	19.3	10	19.3	13.9	37.3
**Abbrev.**	**Bias**	**10**	**D**	10	10	10	10	10	10	10	10
			**f**	10	10	13.9	10	10	13.9	10	19.3
**Abbrev.**	**Bias**	**20**	**D**	10	10	10	10	10	10	10	10
			**f**	193.1	100	268.3	193.1	372.8	193.1	138.9	372.8
**Abbrev.**	**Disp.**	**10**	**D**	71.9	51.8	51.8	51.8	51.8	51.8	51.8	51.8
			**f**	71.9	51.7	138.9	100	193.1	100	71.9	193.1
**Abbrev.**	**Disp.**	**20**	**D**	26.8	26.8	26.8	26.8	26.8	26.8	26.8	26.8
			**f**	138.9	100	268.3	193.1	372.8	193.1	138.9	517.9
**Abbrev.**	**Total**	**10**	**D**	71.9	51.8	51.8	51.8	51.8	51.8	51.8	51.8
			**f**	71.9	51.7	138.9	100	193.1	100	71.9	193.1
**Abbrev.**	**Total**	**20**	**D**	26.8	26.8	26.8	26.8	26.8	26.8	26.8	26.8
