# Supplementary material for: Towards Clinical Translation of Intravoxel Incoherent Motion MRI: Acquisition and Analysis Consensus Recommendations
Source: J Magn Reson Imaging. 2026 Mar 19;63(6):1782–801. doi: 10.1002/jmri.70278 (PMC13175230; doi:10.1002/jmri.70278)
Supplement: Supplementary file 5 — Supplementary Information 5 Estimating f and S0. [file JMRI-63-1782-s002.pdf]

Supplemental Information 5: Estimating *f* and *S0*

There is limited evidence on what is good practice for situations where b=0 s/mm^2 is unavailable, e.g. due to correcting the b-values for the imaging gradients contribution. Our preliminary advice is to follow the first step of the segmented fitting procedure described in the main paper (4.3 Fitting) to estimate *D.* Then, *f* and *S0* are estimated as accurately as possible (see below). Subsequently, one uses the estimated *f* and *S0* as an initial guess and uses the non-linear least squares (NLLS) algorithm with fixed D to fit *f, D**, and *S0.*

Extracting initial estimates of *f* and *S0* can be done, for example, by subtracting the estimated monoexponential fit (*D*) from the signal and fitting an NLLS algorithm, as described by Rashid et al [1, main paper ref 70].

Another option follows the segmented fitting as described in the main paper more closely. One can extrapolate the signal to b=0 s/mm^2 to find an initial guess for *f* for the NLLS fit. One way to do this is to shift all b-values to a new b-value axis, b', such that the lowest b-value (b_{min}) is moved to b=0 s/mm^2:

$$b' = b - b_{min}$$

Use a segmented fit on this axis to get D', f', D*', and S0'. The intercept with b=0 s/mm^2 is now an intercept with b'=- b_{min} on this axis. Hence, the estimated S0 is obtained by replacing b' in the IVIM equation by -b_{min}

$$S0 = S0'\left((1-f')e^{-(-b_{min})D} + f'e^{-(-b_{min})D*}\right)$$

Then, one can calculate the intercept of the mono-exponential curve (describing *D*) by only considering the diffusion aspect of the signal and extrapolating to b'=- b_{min}

$$intercept = S0'(1-f')e^{-(-b_{min})D}$$

And, hence, the estimated *f* is:

$$f = \frac{intercept}{S0} = \frac{(1-f')e^{-(-b_{min})D}}{\left((1-f')e^{-(-b_{min})D} + f'e^{-(-b_{min})D*}\right)}$$

Following the steps described in the main paper 4.3 Fitting, both *D* and *f* are subsequently fixed, and D* is estimated using NLLS.

[1] Rashid IA, Szczepankiewicz F, Gunnlaugsson A, Olsson LE, Brynolfsson P. Effect of inaccurate b-values from imaging gradients on intravoxel incoherent motion. Magn Reson Med. 2025
