# Supplementary material for: Towards Clinical Translation of Intravoxel Incoherent Motion MRI: Acquisition and Analysis Consensus Recommendations
Source: J Magn Reson Imaging. 2026 Mar 19;63(6):1782–801. doi: 10.1002/jmri.70278 (PMC13175230; doi:10.1002/jmri.70278)
Supplement: Supplementary file 6 — Supplementary Information 6 Reporting checklist. [file JMRI-63-1782-s007.pdf]

Supplemental Information 6: Reporting Checklist

Parameters always to be reported:

Acquisition settings:

☐ TE

☐ TR

☐ Diffusion gradient waveform

☐ (Types of) Fat suppression

☐ Voxel size

☐ Field of view

☐ Acceleration options (parallel imaging, half-scan/partial Fourier, compressed sensing, …)

☐ Slice gap

☐ Averages / Repetitions

☐ Receiver coils

☐ Vendor

Reconstruction settings:

☐ Details of pre-processing steps (preferably open access)

☐ Fit algorithm and settings (preferably open access)

☐ Initial guess of fit

☐ Fit constraints

Parameters to be reported if available:

Acquisition settings:

☐ Diffusion time (delay between the leading edges of balanced pulsed magnetic field gradients (Δ))

☐ Duration of pulsed magnetic gradients (δ)

☐ RF shimming

☐ B0 shimming

☐ Echo train length

☐ Echo spacing

☐ Receiver bandwidth

☐ Oversampling factors and directions

☐ Slice acquisition order

☐ Maximum gradient strength and slew rate of the MR system

Reconstruction settings:

☐ Interpolation

☐ Zero filling

☐ Filtering
