# Supplementary material for: Towards Clinical Translation of Intravoxel Incoherent Motion MRI: Acquisition and Analysis Consensus Recommendations
Source: J Magn Reson Imaging. 2026 Mar 19;63(6):1782–801. doi: 10.1002/jmri.70278 (PMC13175230; doi:10.1002/jmri.70278)
Supplement: Supplementary file 7 — Supplementary Information 7 Test–retest studies. [file JMRI-63-1782-s006.pdf]

Supplemental Information 7: Test-retest studies

In the following, a review of literature reports on IVIM phantom, test-retest, and reproducibility studies is provided, which can provide guidance for designing and executing IVIM studies.

Phantom studies

There have been a range of phantom media and geometries constructed and tested to synthesize IVIM contrast, including capillary bundles, sephadex microspheres, cellulose sponges, and sacrificial sucrose networks (1-11) (summary in Synthetic and Physiological Complexity, Ch. 25, pp. 507-523 in reference (12)).

Of these, only two IVIM phantom studies reported reproducibility data in clinical scanners. The novel breast IVIM phantom, designed by Basukala et al (11), was scanned at two sites at 3T scanners from two vendors. At each scanner, the phantom was scanned twice within one session and twice on another day. *D* showed excellent repeatability (< 3%) and reproducibility (< 5%) at the two sites. *f* and *D** exhibited good repeatability (mean of two sites 3.67% and 5.59%, respectively) and moderate reproducibility (mean of two sites 15.96% and 13.3%, respectively). The mean intersite reproducibility (%) of *f*/*D**/*D* was 50.96/13.68/5.59, respectively. Thus results showed promising IVIM reproducibility results similar to ADC, particularly for *D*. The results were reproducible within the sites, and a progressive trend toward reproducibility across sites, except for *f*, *w*hich was affected by different gradient waveforms.

Similarly, Lee et.al. (3) evaluated coefficient of variation over 5 repeated scans in their sephadex bead phantom at variable flow rates. Using segmented fitting, all CVs were below 10%, with lower values for *D* (4.4%) than for *f* (9.0%) and *D** (6.6%).

In-vivo studies

The few reproducibility studies that focused on subjects or patients are detailed as follows. Those studies focused on the liver, kidney, breast, and brain.

In 2014, Dyvorne et al (13) concluded that 4 optimized b-values can be used to estimate IVIM parameters in the **liver** with significantly shorter acquisition time (up to 75%), without substantial degradation of IVIM parameter precision and reproducibility compared to the 16 b-value acquisition used as the reference.

In 2015, Grech-Sollars et al (14) scanned **ice-water phantom** and nine **healthy volunteers** across five centers on eight scanners (four Siemens 1.5T, four Philips 3T). The mean ADC, IVIM parameters (*D* and *f*) were measured in gray matter, white matter, and specific **brain** sub-regions. ADC and *D* showed good intra-scanner and inter-scanner reproducibility, while *f* had a poorer inter-scanner coefficient of variation when scanners of different field strengths were combined, and the parameter was also affected by the scan acquisition resolution.

Similar results with poor reproducibility of *D** and *f* and good reproducibility for *D*/ADC were observed in hepatocellular carcinoma (**HCC**) and **liver** parenchyma by Kakite et al (15). These findings may have implications for trials using DWI in HCC.

Chevallier et al (16) reported that removal of motion-contaminated and/or poorly fitted image data improves IVIM parameter reproducibility for **liver** parenchyma.

Supplemental Information to “Towards Clinical Translation of Intravoxel Incoherent Motion MRI: Acquisition and Analysis Consensus Recommendations” by Sigmund et al.

In 2018, Iima M et al (17) mentioned that the data in their study suggest that a limited MRI protocol using a few b-values might be relevant in a clinical setting for the estimation of non-Gaussian diffusion MRI parameters in normal **breast** tissue and breast lesions.

Pan J et al (18) reported excellent reproducibility evaluation for ADC and *D*, good for *f*, and poor for *D** derived from IVIM was performed in **renal tumors**, normal **renal cortex**, and **medulla**. *D** has limited reliability, and scan-rescan reproducibility should be improved.

In 2021, de Boer et al (19) concluded that most MRI measures of **renal function and structure** (with the exception of *f* and perfusion as measured by DCE) were below 13%, which is comparable to standard clinical tests in nephrology.

Li et al (20) mentioned that for the **liver** all reproducibility indicators slightly favored free breathing (FB) imaging.

In 2024, Vasquez et al (21) reported that the simultaneous multislice acquisitions had significantly less variability and higher ICCs of *D*, higher SNR, less distortion, and reduced scan time compared to EPI in the **liver**.

References:

1. Karampinos DC, King KF, Sutton BP, Georgiadis JG. Intravoxel partially coherent motion technique: characterization of the anisotropy of skeletal muscle microvasculature. J Magn Reson Imaging. 2010;31(4):942-53.
2. Cho GY, Kim S, Jensen JH, Storey P, Sodickson DK, Sigmund EE. A versatile flow phantom for intravoxel incoherent motion MRI. Magn Reson Med. 2012;67(6):1710-20.
3. Lee JH, Cheong H, Lee SS, Lee CK, Sung YS, Huh JW, et al. Perfusion Assessment Using Intravoxel Incoherent Motion-Based Analysis of Diffusion-Weighted Magnetic Resonance Imaging: Validation Through Phantom Experiments. Invest Radiol. 2016;51(8):520-8.
4. Ohno N, Miyati T, Chigusa T, Usui H, Ishida S, Hiramatsu Y, et al. Technical Note: Development of a cranial phantom for assessing perfusion, diffusion, and biomechanics. Med Phys. 2017;44(5):1646-54.
5. Wetscherek A, Stieltjes B, Laun FB. Flow-compensated intravoxel incoherent motion diffusion imaging. Magn Reson Med. 2015;74(2):410-9.
6. Lorenz CH, Pickens DR, 3rd, Puffer DB, Price RR. Magnetic resonance diffusion/perfusion phantom experiments. Magn Reson Med. 1991;19(2):254-60.
7. Le Bihan D, Breton E, Lallemand D, Aubin ML, Vignaud J, Laval-Jeantet M. Separation of diffusion and perfusion in intravoxel incoherent motion MR imaging. Radiology. 1988;168(2):497-505.
8. Maki JH, MacFall JR, Johnson GA. The use of gradient flow compensation to separate diffusion and microcirculatory flow in MRI. Magn Reson Med. 1991;17(1):95-107.
9. Fujita N, Harada K, Sakurai K, Akai Y, Kozuka T. Separation of diffusion and slow flow effects by use of flow rephasing and dephasing. Magn Reson Med. 1992;24(1):109-22.
10. Ahn CB, Lee SY, Nalcioglu O, Cho ZH. The effects of random directional distributed flow in nuclear magnetic resonance imaging. Med Phys. 1987;14(1):43-8.
11. Basukala D, Mikheev A, Sevilimedu V, Gilani N, Moy L, Pinker K, et al. Multisite MRI Intravoxel Incoherent Motion Repeatability and Reproducibility across 3 T Scanners in a Breast Diffusion Phantom: A BReast Intravoxel Incoherent Motion Multisite (BRIMM) Study. J Magn Reson Imaging. 2024;59(6):2226-37.
12. Le Bihan D, Iima M, Federau C, Sigmund EE. Intravoxel Incoherent Motion (IVIM) MRI: Principles and Applications: Pan Stanford Publishing; 2019.

13. Dyvorne H, Jajamovich G, Kakite S, Kuehn B, Taouli B. Intravoxel incoherent motion diffusion imaging of the liver: optimal b-value subsampling and impact on parameter precision and reproducibility. Eur J Radiol. 2014;83(12):2109-13.
14. Grech-Sollars M, Hales PW, Miyazaki K, Raschke F, Rodriguez D, Wilson M, et al. Multi-centre reproducibility of diffusion MRI parameters for clinical sequences in the brain. NMR Biomed. 2015;28(4):468-85.
15. Kakite S, Dyvorne H, Besa C, Cooper N, Facciuto M, Donnerhack C, et al. Hepatocellular carcinoma: short-term reproducibility of apparent diffusion coefficient and intravoxel incoherent motion parameters at 3.0T. J Magn Reson Imaging. 2015;41(1):149-56.
16. Chevallier O, Zhou N, He J, Loffroy R, Wang YXJ. Removal of evidential motion-contaminated and poorly fitted image data improves IVIM diffusion MRI parameter scan-rescan reproducibility. Acta Radiol. 2018;59(10):1157-67.
17. Iima M, Kataoka M, Kanao S, Onishi N, Kawai M, Ohashi A, et al. Intravoxel Incoherent Motion and Quantitative Non-Gaussian Diffusion MR Imaging: Evaluation of the Diagnostic and Prognostic Value of Several Markers of Malignant and Benign Breast Lesions. Radiology. 2018;287(2):432-41.
18. Pan J, Zhang H, Man F, Shen Y, Wang Y, Zhong Y, et al. Measurement and scan reproducibility of parameters of intravoxel incoherent motion in renal tumor and normal renal parenchyma: a preliminary research at 3.0 T MR. Abdom Radiol (NY). 2018;43(7):1739-48.
19. de Boer A, Harteveld AA, Stemkens B, Blankestijn PJ, Bos C, Franklin SL, et al. Multiparametric Renal MRI: An Intrasubject Test-Retest Repeatability Study. J Magn Reson Imaging. 2021;53(3):859-73.
20. Li XM, Ma FZ, Quan XY, Zhang XC, Xiao BH, Wang YXJ. Repeatability and reproducibility comparisons of liver IVIM imaging with free-breathing or respiratory-triggered sequences. NMR Biomed. 2024;37(4):e5080.
21. Vasquez JA, Brown M, Woolsey M, Abdul-Ghani M, Katabathina V, Deng S, et al. Reproducibility and Repeatability of Intravoxel Incoherent Motion MRI Acquisition Methods in Liver. J Magn Reson Imaging. 2024;60(4):1691-703.
